# Whole-Body Hypothermia, Cerebral Magnetic Resonance Biomarkers, and Outcomes in Neonates With Moderate or Severe Hypoxic-Ischemic Encephalopathy Born at Tertiary Care Centers vs Other Facilities: A Nested Study Within a Randomized Clinical Trial

**DOI:** 10.1001/jamanetworkopen.2023.12152

**Published:** 2023-05-08

**Authors:** Sudhin Thayyil, Paolo Montaldo, Vaisakh Krishnan, Phoebe Ivain, Stuti Pant, Peter J. Lally, Prathik Bandiya, Naveen Benkappa, Chinnathambi N. Kamalaratnam, Rema Chandramohan, Swati Manerkar, Jayshree Mondkar, Ismat Jahan, Sadeka C. Moni, Mohammod Shahidullah, Ranmali Rodrigo, Samanmali Sumanasena, Radhika Sujatha, Constance Burgod, Reema Garegrat, Munirah Mazlan, Ismita Chettri, Sathyanathan Babu Peter, Anagha R. Joshi, Ravi Swamy, Kling Chong, Ronit R. Pressler, Paul Bassett, Seetha Shankaran

**Affiliations:** 1 Centre for Perinatal Neuroscience, Department of Brain Sciences, Imperial College London, London, United Kingdom; 2 Neonatal Unit, University of Campania Luigi Vanvitelli, Naples, Italy; 3 Neonatal Unit, Indira Gandhi Institute of Child Health, Bengaluru, India; 4 Department of Pediatrics, Madras Medical College, Chennai, India; 5 Neonatal Unit, Lokmanya Tilak Municipal Medical College, Mumbai, India; 6 Neonatal Unit, Bangabandhu Sheikh Mujib Medical University, Dhaka, Bangladesh; 7 Department of Pediatrics, University of Kelaniya, Kelaniya, Sri Lanka; 8 Neonatal Unit, Sree Avittom Thirunal Hospital, Government Medical College, Thiruvananthapuram, India; 9 Department of Radiology, Barnard Institute, Madras Medical College, Chennai, India; 10 Department of Radiology, Lokmanya Tilak Municipal Medical College, Mumbai, India; 11 Department of Neuroradiology, Great Ormond Street Hospital, London, United Kingdom; 12 Department of Neurophysiology, Great Ormond Street Hospital, London, United Kingdom; 13 Statsconsultancy Ltd, Amersham, United Kingdom; 14 Division of Neonatal-Perinatal Medicine, Wayne State University, Detroit, Michigan

## Abstract

**Importance:**

The association between place of birth and hypothermic neuroprotection after hypoxic-ischemic encephalopathy (HIE) in low- and middle-income countries (LMICs) is unknown.

**Objective:**

To ascertain the association between place of birth and the efficacy of whole-body hypothermia for protection against brain injury measured by magnetic resonance (MR) biomarkers among neonates born at a tertiary care center (inborn) or other facilities (outborn).

**Design, Setting, and Participants:**

This nested cohort study within a randomized clinical trial involved neonates at 7 tertiary neonatal intensive care units in India, Sri Lanka, and Bangladesh between August 15, 2015, and February 15, 2019. A total of 408 neonates born at or after 36 weeks’ gestation with moderate or severe HIE were randomized to receive whole-body hypothermia (reduction of rectal temperatures to between 33.0 °C and 34.0 °C; hypothermia group) for 72 hours or no whole-body hypothermia (rectal temperatures maintained between 36.0 °C and 37.0 °C; control group) within 6 hours of birth, with follow-up until September 27, 2020.

**Exposure:**

3T MR imaging, MR spectroscopy, and diffusion tensor imaging.

**Main Outcomes and Measures:**

Thalamic *N*-acetyl aspartate (NAA) mmol/kg wet weight, thalamic lactate to NAA peak area ratios, brain injury scores, and white matter fractional anisotropy at 1 to 2 weeks and death or moderate or severe disability at 18 to 22 months.

**Results:**

Among 408 neonates, the mean (SD) gestational age was 38.7 (1.3) weeks; 267 (65.4%) were male. A total of 123 neonates were inborn and 285 were outborn. Inborn neonates were smaller (mean [SD], 2.8 [0.5] kg vs 2.9 [0.4] kg; *P* = .02), more likely to have instrumental or cesarean deliveries (43.1% vs 24.7%; *P* = .01), and more likely to be intubated at birth (78.9% vs 29.1%; *P* = .001) than outborn neonates, although the rate of severe HIE was not different (23.6% vs 17.9%; *P* = .22). Magnetic resonance data from 267 neonates (80 inborn and 187 outborn) were analyzed. In the hypothermia vs control groups, the mean (SD) thalamic NAA levels were 8.04 (1.98) vs 8.31 (1.13) among inborn neonates (odds ratio [OR], −0.28; 95% CI, −1.62 to 1.07; *P* = .68) and 8.03 (1.89) vs 7.99 (1.72) among outborn neonates (OR, 0.05; 95% CI, −0.62 to 0.71; *P* = .89); the median (IQR) thalamic lactate to NAA peak area ratios were 0.13 (0.10-0.20) vs 0.12 (0.09-0.18) among inborn neonates (OR, 1.02; 95% CI, 0.96-1.08; *P* = .59) and 0.14 (0.11-0.20) vs 0.14 (0.10-0.17) among outborn neonates (OR, 1.03; 95% CI, 0.98-1.09; *P* = .18). There was no difference in brain injury scores or white matter fractional anisotropy between the hypothermia and control groups among inborn or outborn neonates. Whole-body hypothermia was not associated with reductions in death or disability, either among 123 inborn neonates (hypothermia vs control group: 34 neonates [58.6%] vs 34 [56.7%]; risk ratio, 1.03; 95% CI, 0.76-1.41), or 285 outborn neonates (hypothermia vs control group: 64 neonates [46.7%] vs 60 [43.2%]; risk ratio, 1.08; 95% CI, 0.83-1.41).

**Conclusions and Relevance:**

In this nested cohort study, whole-body hypothermia was not associated with reductions in brain injury after HIE among neonates in South Asia, irrespective of place of birth. These findings do not support the use of whole-body hypothermia for HIE among neonates in LMICs.

**Trial Registration:**

ClinicalTrials.gov Identifier: NCT02387385

## Key Points

**Question:**

Is place of birth associated with the efficacy of whole-body hypothermia in neonates with hypoxic-ischemic encephalopathy (HIE) in low- and middle-income countries?

**Findings:**

In this nested cohort study within a randomized clinical trial recruiting 408 neonates with moderate or severe HIE from 7 tertiary neonatal intensive care units in South Asia, whole-body hypothermia was not associated with reductions in brain injury measured by magnetic resonance biomarkers at age 2 weeks among neonates born at either a tertiary care center or other facilities.

**Meaning:**

These findings do not support the use of whole-body hypothermia for neonates with HIE in low- and middle-income countries.

## Teaser

This nested cohort study within a clinical trial assesses the association of whole-body hypothermia with magnetic resonance biomarkers and outcomes among neonates born at a tertiary care center vs other facilities in South Asia.

## Introduction

Brain injury associated with birth depression, otherwise known as hypoxic-ischemic encephalopathy (HIE), is the most common cause of death or lifelong neurodisability among neonates born at full term,^1^ accounting for approximately 1 million deaths every year. More than 90% of the disease burden is in low- and middle-income countries (LMICs). In high-income countries (HICs), several clinical trials^2,3,4^ have reported that selective head or whole-body hypothermia reduces brain injury, death, or neurodisability at ages 18 to 24 months. These trials have included both neonates born within (inborn) or outside (outborn) a tertiary neonatal intensive care unit (NICU) with facilities for induced hypothermia, and hypothermia was found to improve clinical outcomes irrespective of place of birth.^5^ Unlike tertiary care centers, nontertiary care centers in LMICs often have clinicians with limited neonatal expertise in addition to limited resources and a lack of dedicated neonatal transport, which may contribute to suboptimal postresuscitation care and aggravation of the brain injury before transfer to a cooling center.^6^

Magnetic resonance (MR) spectroscopic biomarkers provide an objective quantitative assessment of brain injury^7,8^ and can quantify neuroprotection using substantially smaller samples than clinical outcome measures. In this subgroup analysis of the Hypothermia for Encephalopathy in Low- and Middle-Income Countries (HELIX) trial, we examined the association of place of birth with the efficacy of whole-body hypothermia for protection against brain injury using quantitative 3T MR biomarkers.

## Methods

### Study Design and Participants

HELIX was an open-label randomized clinical trial (RCT) that recruited 408 neonates with moderate or severe HIE from 7 tertiary NICUs in India, Sri Lanka, and Bangladesh between August 15, 2015, and February 15, 2019,^9^ with follow-up data through September 27, 2020 (the trial protocol is provided in Supplement 1, and the study flowchart is available in eFigure 1 in Supplement 2). The trial was approved by the Imperial College Research Ethics Committee and the ethics committees at all participating sites (Supplement 1). Parental informed consent was obtained for all neonates. This study followed the Consolidated Standards of Reporting Trials (CONSORT) reporting guideline for RCTs.

Neonates born at or after 36 weeks’ gestation with a birth weight of 1.8 kg or more who required continued resuscitation at age 5 minutes, had a 5-minute Apgar score less than 6, or both (or had lack of cry by age 5 minutes for home birth) were recruited if there was evidence of moderate or severe HIE on structured neurological examination performed between 1 and 6 hours after birth by a certified examiner. We excluded neonates who had no heart rate at age 10 minutes despite adequate resuscitation, those with major life-threatening congenital malformations, or those with parents who were unable to attend follow-up assessments. Neonates allocated to the hypothermia group had a controlled reduction of rectal temperatures to between 33.0 °C and 34.0 °C within 6 hours of birth that was continued for 72 hours followed by rewarming at 0.5 °C per hour using an neonate cooling and rewarming system (Tecotherm Neo; Inspiration Health Care [now International Biomedical]). The control group had rectal temperatures maintained between 36.0 °C and 37.0 °C. Neonates born at the participating tertiary NICUs with facilities for whole-body hypothermia were defined as inborn, and neonates born elsewhere (including other tertiary care centers, secondary care centers, primary care facilities, or at home) were defined as outborn. The primary outcome of the HELIX trial was death or moderate or severe disability assessed between 18 and 22 months by neurodevelopmental pediatricians masked to the allocation and neuroimaging data.^9^

### Procedures

Before recruitment, all MR scanners were harmonized using phantom studies and adult volunteers, and spectroscopic data were acquired using previously validated cross-platform sequences that can be used in 3T scanners from all 3 common manufacturers.^8^ This included Magnetom Skyra 3T (Siemens Healthineers) (4 sites), Magnetom Verio 3T (Siemens Healthineers) (1 site), Philips Achieva 3T (Philips Medical Systems) (1 site), and SIGNA 3T (GE Healthcare) (1 site) MR scanners.All neonates underwent MR scans at age 2 weeks unless they died before the scan or parents declined consent.

The MR protocol (acquisition time) included 3-dimensional T1- and T2-weighted MR imaging (15 minutes) in the axial and sagittal planes, diffusion tensor imaging (DTI) (7 minutes), proton MR spectroscopic metabolite peak area ratios (PARs) (7 minutes), and metabolite absolute concentrations (13 minutes). We acquired MR spectroscopy in a single 15 × 15 × 15 mm^3^ voxel centered on the left thalamus, and we centrally postprocessed the pseudonymized raw MR data while masked to the treatment allocation and clinical outcomes.^10^ We excluded poor-quality data based on predefined criteria before data analysis to avoid any selection bias. We analyzed the MR spectroscopic data using LCModel software, version 6.3 (Stephen Provencher),^11^ and we analyzed the DTI data using functional MR imaging in the FMRIB Software Library, version 6.0 (FMRIB Analysis Group).^12^ Voxelwise statistical analysis of the fractional anisotropic (FA) data was conducted using tract-based spatial statistics. All MR images were centrally reported using a previously validated scoring system.^13^

### Statistical Analysis

The baseline demographic and clinical characteristics of inborn and outborn neonates in the hypothermia and control groups were compared using the unpaired *t* test or Mann-Whitney test for continuous variables and the χ^2^ test or Fisher exact test for categorical variables. Race and ethnicity data were not collected because the study population was all from India, Sri Lanka, and Bangladesh (all of Asian race). Binary outcomes were compared between groups using the χ^2^ test, with group differences expressed as risk ratios (RRs) with 95% CIs. Ordinal logistic regression was used for the analysis of ordinal outcomes (including MR biomarkers), with the group differences expressed as odds ratios (ORs) with corresponding 95% CIs. Continuous outcomes were compared between groups using the unpaired *t* test. Outcomes with positively skewed distribution were log transformed for the analysis. Cox regression was used for the analysis of time of death, with survival as the outcome. The significance threshold was 2-tailed *P* = .05. Data were analyzed using Stata software, version 17.0 (StataCorp LLC).

## Results

### Study Population

Among 408 neonates, the mean (SD) gestational age was 38.7 (1.3) weeks; 267 (65.4%) were male and 141 (34.6%) were female. A total of 123 neonates were inborn and 285 were outborn. Although the baseline clinical characteristics of the hypothermia and control groups were not significantly different (Table 1), there were some differences between inborn and outborn neonates. Compared with the outborn neonates, the inborn neonates were born to older mothers (mean [SD] age, 25.7 [5.0] years vs 23.7 [4.2] years; *P* = .001); more of them had meconium-stained amniotic fluid (47 of 123 neonates [38.2%] vs 63 of 269 [23.4%]; *P* < .001), reduced fetal movements (13 of 114 neonates [11.4%] vs 11 of 221 [5.0%]; *P* < .001), and fetal heart rate decelerations (16 of 116 neonates [13.8%] vs 7 of 208 [3.4%]; *P* < .001); and more of them had instrumental or cesarean deliveries (53 of 123 neonates [43.1%] vs 70 of 283 [24.7%]; *P* = .01). The inborn neonates also had lower birth weight (mean [SD], 2.8 [0.5] kg vs 2.9 [0.4] kg; *P* = .02) and more often required intubation at birth (97 of 123 neonates [78.9%] vs 81 of 278 [29.1%]; *P* = .001) than the outborn neonates. The inborn neonates were also admitted to the NICU earlier (median [IQR], 30 [15-50] minutes vs 180 [123-240] minutes; *P* = .001) and had lower rates of seizures at randomization (75 of 122 neonates [61.5%] vs 224 of 285 [78.6%]; *P* = .001) than outborn neonates (eTable 1 in Supplement 2). However, the rate of severe HIE was not different between inborn and outborn groups (29 neonates [23.6%] vs 51 [17.9%]; *P* = .22).

**Table 1.** Baseline Participant Characteristics

Characteristic	Participants, No./total No. (%)		
	Hypothermia group	Control group	*P* value
**Inborn neonates (n = 123)**			
Total participants, No.	62	61	NA
Maternal characteristics and prenatal conditions			
Age, mean (SD), y^a^	26.1 (5.0)	25.4 (5.2)	.45
Booked pregnancies^b^	62/62 (100)	60/61 (98.4)	.50
Primigravida	37/62 (59.7)	30/61 (49.2)	.24
Diabetes	0	1/61 (1.6)	.50
Pregnancy-induced hypertension	5/62 (8.1)	3/61 (4.9)	.47
Thyroid disorders	0	0	>.99
Complications of delivery^c^	20/62 (32.3)	23/60 (38.3)	.48
Maternal pyrexia	2/61 (3.3)	3/60 (5.0)	.68
Rupture of membranes >24 h	0	0	>.99
Meconium-stained amniotic fluid	28/62 (45.2)	19/61 (31.1)	.11
Reduced fetal movements	10/60 (16.7)	3/54 (5.6)	.06
Fetal heart rate decelerations	8/58 (13.8)	8/58 (13.8)	>.99
Funisitis	12/61 (19.7)	9/60 (15.0)	.50
Perinatal sentinel events^c^			
Cord prolapse	1/62 (1.6)	3/61 (4.9)	.37
Cord around neck	1/62 (1.6)	1/61 (1.6)	>.99
Prolonged second stage	1/62 (1.6)	6/61 (9.8)	.06
Obstructed labor	1/62 (1.6)	1/61 (1.6)	>.99
Shoulder dystocia	0	0	>.99
Antepartum hemorrhage	3/62 (4.8)	2/61 (3.3)	.66
Mode of delivery			
Instrumental	8/62 (12.9)	10/61 (16.4)	.62
Prelabor cesarean	1/62 (1.6)	2/61 (3.3)	
In-labor cesarean	19/62 (30.6)	13/61 (21.3)	
Spontaneous vaginal	34/62 (54.8)	36/61 (59.0)	
Condition at birth			
Cord blood pH at delivery, mean (SD)^d^	6.91 (0.16)	6.93 (0.21)	.73
Apgar score at 5 min, median (IQR)^e^	5 (3-5)	4 (4-5)	.68
Apgar score at 10 min, median (IQR)^f^	6 (4-7)	6 (5-7)	.84
Endotracheal ventilation at birth	48/62 (77.4)	49/61 (80.3)	.69
Neonate characteristics and conditions			
Birth weight, mean (SD), g^g^	2735 (447)	2896 (464)	.05
Birth weight <2 SDs	16/62 (25.8)	11/61 (18.0)	.30
Head circumference, mean (SD), cm^h^	33.9 (1.6)	34.3 (1.9)	.31
Head circumference <2 SDs	4/62 (6.5)	2/60 (3.3)	.68
Gestational age of neonate, mean (SD), wk^g^	39.0 (1.4)	38.9 (1.6)	.91
Age at admission to the NICU, median (IQR), min^g^	31 (15-46)	33 (18-50)	.85
Sex			
Male	39/62 (62.9)	41/61 (67.2)	.62
Female	23/62 (37.1)	20/61 (32.8)	
Moderate encephalopathy	44/62 (71.0)	50/61 (82.0)	.15
Severe encephalopathy	18/62 (29.0)	11/61 (18.0)	
Clinical seizures at admission	39/62 (62.9)	36/61 (59.0)	.66
**Outborn neonates (n = 285)** ^i^			
Total participants, No.	140	145	NA
Maternal characteristics and prenatal conditions			
Age, mean (SD), y^j^	23.8 (4.2)	23.8 (4.3)	.94
Booked pregnancies^b^	134/138 (97.1)	130/144 (90.3)	.03
Primigravida	81/139 (58.3)	87/145 (60.0)	.77
Diabetes	1/140 (0.7)	0	.49
Pregnancy-induced hypertension	4/140 (2.9)	1/145 (0.7)	.16
Thyroid disorders	0	1/145 (0.7)	>.99
Complications of delivery^c^	18/130 (13.8)	17/137 (12.4)	.73
Maternal pyrexia	1/128 (0.8)	2/139 (1.4)	>.99
Rupture of membranes >24 h	1/126 (0.8)	3/136 (2.2)	.35
Meconium-stained amniotic fluid	32/130 (24.6)	31/139 (22.3)	.65
Reduced fetal movements	6/109 (5.5)	5/112 (4.5)	.72
Fetal heart rate decelerations	6/106 (5.7)	1/102 (1.0)	.12
Funisitis	25/120 (20.8)	20/125 (16.0)	.33
Perinatal sentinel events^c^			
Cord prolapse	3/140 (2.1)	3/145 (2.1)	.96
Cord around neck	1/140 (0.7)	5/145 (3.4)	.21
Prolonged second stage	1/140 (0.7)	3/145 (2.1)	.62
Obstructed labor	1/140 (0.7)	2/145 (1.4)	>.99
Shoulder dystocia	1/140 (0.7)	0	.49
Antepartum hemorrhage^k^	3/140 (2.1)	0	.07
Mode of delivery			
Instrumental	13/139 (9.4)	9/144 (6.3)	.29
Prelabor cesarean	0	0	
In-labor cesarean	27/139 (19.4)	21/144 (14.6)	
Spontaneous vaginal	99/139 (71.2)	114/144 (79.2)	
Condition at birth			
Cord blood pH at delivery, mean (SD)^l^	7.02 (0.41)	7.06 (0.21)	.79
Apgar score at 5 min, median (IQR)^m^	5 (4-6)	5 (4-6)	.09
Apgar score at 10 min, median (IQR)^n^	5 (4-7)	6 (4-7)	.39
Endotracheal ventilation at birth	41/138 (29.7)	40/140 (28.6)	.84
Neonate characteristics and conditions			
Birth weight, mean (SD), g	2892 (444)	2956 (452)	.23
Birth weight <2 SDs	20/140 (14.3)	16/145 (11.0)	.41
Head circumference, mean (SD), cm^o^	34.3 (1.4)	34.3 (1.4)	.59
Head circumference <2 SDs	4/139 (2.9)	4/145 (2.8)	>.99
Gestational age of neonate, mean (SD), wk^p^	38.8 (1.2)	39.0 (1.1)	.18
Age at admission to the NICU, median (IQR), min	180 (120-240)	189 (139-255)	.16
Sex			
Male	93/140 (66.4)	94/145 (64.8)	.78
Female	47/140 (33.6)	51/145 (35.2)	
Moderate encephalopathy	117/140 (83.6)	117/145 (80.7)	.53
Severe encephalopathy	23/140 (16.4)	28/145 (19.3)	
Clinical seizures at admission	110/140 (78.6)	114/145 (78.6)	.99

Abbreviations: NA, not applicable; NICU, neonatal intensive care unit.

^a^ Among 60 participants in the hypothermia group and 57 in the control group.

^b^ Booked pregnancies had antenatal follow-up visits at the recruiting hospital or any other health-care facility.

^c^ Not mutually exclusive.

^d^ Among 16 participants in the hypothermia group and 17 in the control group.

^e^ Among 61 participants in the hypothermia group and 61 in the control group.

^f^ Among 52 participants in the hypothermia group and 46 in the control group.

^g^ Among 62 participants in the hypothermia group and 61 in the control group.

^h^ Among 62 participants in the hypothermia group and 60 in the control group.

^i^ Born at other tertiary medical college hospitals (65 neonates), secondary district hospitals (124 neonates), primary care centers (44 neonates), private hospitals (40 neonates), unnamed hospitals (2 neonates), or home (10 neonates) and transferred to the cooling center within 6 hours of birth.

^j^ Among 139 participants in the hypothermia group and 139 in the control group.

^k^ Three participants had abruption.

^l^ Among 6 participants in the hypothermia group and 7 in the control group.

^m^ Among 76 participants in the hypothermia group and 74 in the control group.

^n^ Among 13 participants in the hypothermia group and 21 in the control group.

^o^ Among 139 participants in the hypothermia group and 145 in the control group.

^p^ Among 140 participants in the hypothermia group and 144 in the control group.

**Figure 1.**
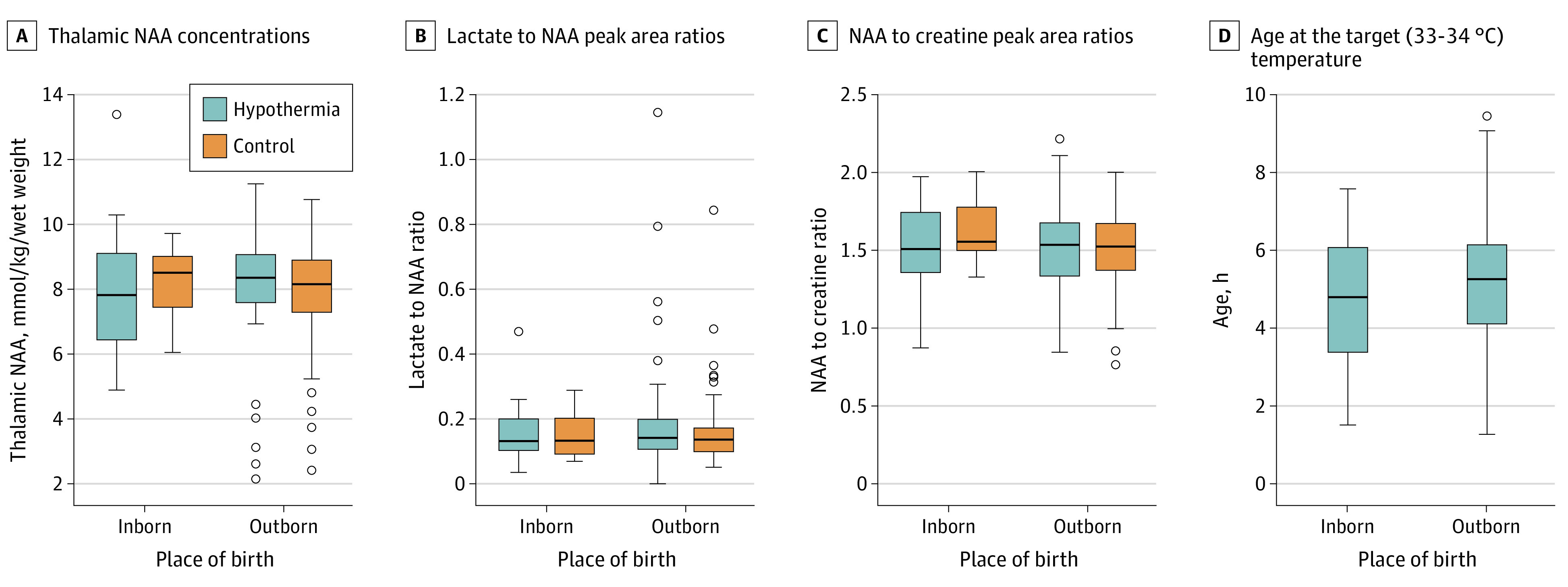
Association of Whole-Body Hypothermia With Magnetic Resonance Spectroscopic Biomarkers and Time to Target Temperature Among Inborn vs Outborn Neonates Horizontal lines across bars represent medians, circles represent outliers, and whiskers represent ranges. NAA indicates *N*-acetyl aspartate.

Temperature profiles are provided in Figure 1 and eFigure 2 in Supplement 2. Among 202 neonates, the target rectal temperature of 34 °C or lower was achieved by 6 hours in 144 neonates (71.3%), between 6 and 7 hours in 39 (19.3%), between 7 and 8 hours in 12 (5.9%), between 8 and 9 hours in 4 (2.0%), and between 9 and 10 hours in 2 (1.0%). Among the 62 inborn neonates in the hypothermia group, the target rectal temperature of 34 °C or lower was achieved by 6 hours in 46 neonates (74.2%), between 6 and 7 hours in 12 (19.4%), and between 7 and 8 hours in 4 (6.5%). Among the 140 outborn neonates in the hypothermia group, the target rectal temperature of 34 °C or lower was achieved by 6 hours in 98 neonates (70.0%), between 6 and 7 hours in 27 (19.3%), between 7 and 8 hours in 8 (5.7%), between 8 and 9 hours in 4 (2.9%), and between 9 and 10 hours in 2 (1.4%). One outborn neonate in the hypothermia group did not receive cooling.

### MR Biomarker Analysis Based on Place of Birth

Of 408 neonates recruited, 114 (27.9%) died before MR imaging, and 10 parents declined consent. The remaining 284 neonates received MR imaging; of those, data from 267 (94.0%) were analyzed. Neonates who did not receive MR imaging (n = 124) had more severe encephalopathy (50 neonates [40.3%] vs 30 [10.6%]), a higher mortality rate (122 neonates [98.4%] vs 25 [8.8%]), and a higher rate of death or disability (122 neonates [98.4%] vs 70 [24.6%]) compared with those who received MR imaging (*P* < .001 for all comparisons) (eTable 2 in Supplement 2).

Of the 123 inborn neonates, 40 died and 1 was discharged against medical advice before the scan. The remaining 82 neonates (66.7%) received MR imaging; of those, data from 80 neonates (37 in the hypothermia group and 43 in the control group) were available for analysis. The scans were performed at a median (IQR) age of 12.0 (9.7-16.2) days in the hypothermia group and 12.0 (10.0-13.0) days in the control group.

Among inborn neonates, the mean (SD) absolute levels of thalamic *N*-acetyl aspartate (NAA) were 8.04 (1.98) mmol/kg wet weight in the hypothermia group and 8.31 (1.13) mmol/kg wet weight in the control group (OR, −0.28; 95% CI, −1.62 to 1.07; *P* = .68), and the median (IQR) thalamic lactate to NAA PARs were 0.13 (0.10-0.20) in the hypothermia group and 0.12 (0.09-0.18) in the control group (OR, 1.02; 95% CI, 0.96-1.08; *P* = .59). There was no difference in white matter FA at any brain region between inborn neonates in the hypothermia and control groups (eFigure 3 in Supplement 2). The brain injury scores based on conventional T1- and T2-weighted images in the basal ganglia, white matter, or cerebral cortex did not reveal any differences between inborn neonates in the hypothermia and control groups (Table 2).

**Table 2.** Association of Whole-Body Hypothermia With Conventional MR Biomarkers and MR Spectroscopic Biomarkers

Outcome	Category	Participants, No. (%)		OR or MD (95% CI)^a^	*P* value
		Hypothermia group	Control group		
**Inborn neonates (n = 76)**					
Total participants, No.		37	39		
Conventional MR imaging					
Basal ganglia and thalamic injury	0	27 (73.0)	30 (76.9)	1.02 (0.37 to 2.86)	.97
	1	4 (10.8)	0		
	2	3 (8.1)	2 (5.1)		
	3	3 (8.1)	7 (17.9)		
Posterior limb of internal capsule	Normal	30 (81.1)	30 (76.9)	0.74 (0.25 to 2.25)	.60
	Equivocal	2 (5.4)	1 (2.6)		
	Abnormal	5 (13.5)	8 (20.5)		
White matter injury	0	7 (18.9)	3 (7.7)	0.49 (0.20 to 1.21)	.12
	1	4 (10.8)	5 (12.8)		
	2	22 (59.5)	22 (56.4)		
	3	4 (10.8)	9 (23.1)		
Cortical injury	0	24 (64.9)	27 (69.2)	1.02 (0.40 to 2.61)	.97
	1	8 (21.6)	3 (7.7)		
	2	1 (2.7)	2 (5.1)		
	3	4 (10.8)	7 (17.9)		
MR spectroscopy					
Thalamic NAA, mean (SD), mmol/kg wet weight		8.04 (1.98)	8.31 (1.13)	−0.28 (−1.62 to 1.07)	.68
NAA to choline PAR, mean (SD)		0.86 (0.11)	0.92 (0.12)	−0.06 (−0.15 to 0.03)	.16
NAA to creatinine PAR, mean (SD)		1.54 (0.28)	1.65 (0.21)	−0.11 (−0.30 to 0.09)	.26
Thalamic lactate to NAA PAR, median (IQR)		0.13 (0.10 to 0.20)	0.12 (0.09 to 0.18)	1.02 (0.96 to 1.08)	.59
**Outborn neonates (n = 173)**					
Total participants, No.		79	94		
Conventional MR imaging					
Basal ganglia and thalamic injury	0	63 (79.7)	72 (76.6)	0.91 (0.44 to 1.87)	.80
	1	3 (3.8)	10 (10.6)		
	2	7 (8.9)	8 (8.5)		
	3	6 (7.6)	4 (4.3)		
Posterior limb of internal capsule	Normal	65 (82.3)	76 (80.9)	0.92 (0.43 to 1.99)	.84
	Equivocal	3 (3.8)	5 (5.3)		
	Abnormal	11 (13.9)	13 (13.8)		
White matter injury	0	15 (19.0)	27 (28.7)	1.26 (0.73 to 2.17)	.41
	1	23 (29.1)	20 (21.3)		
	2	33 (41.8)	39 (41.5)		
	3	8 (10.1)	8 (8.5)		
Cortical injury	0	62 (78.5)	64 (68.1)	0.62 (0.31 to 1.23)	.17
	1	10 (12.7)	20 (21.3)		
	2	2 (2.5)	7 (7.4)		
	3	5 (6.3)	3 (3.2)		
MR spectroscopy					
Thalamic NAA, mean (SD), mmol/kg wet weight		8.03 (1.89)	7.99 (1.72)	0.05 (−0.62 to 0.71)	.89
NAA to choline PAR, mean (SD)		0.83 (0.21)	0.84 (0.16)	−0.01 (−0.07 to 0.06)	.85
NAA to creatinine PAR, mean (SD)		1.51 (0.30)	1.50 (0.27)	0.02 (−0.08 to 0.11)	.74
Thalamic lactate to NAA PAR, median (IQR)		0.14 (0.11 to 0.20)	0.14 (0.10 to 0.17)	1.03 (0.98 to 1.09)	.18

Abbreviations: MD, mean difference; MR, magnetic resonance; NAA, *N*-acetyl aspartate; OR, odds ratio; PAR, peak area ratio.

^a^ Odds ratios (calculated as the odds of being in next highest outcome category for the hypothermia group relative to the odds of being in next highest outcome category for the control group) were reported for conventional MR imaging. MDs (calculated as cooling group values minus control group values) were reported for MR spectroscopy.

Of the 285 outborn neonates, 74 died, 7 were discharged against medical advice before MR imaging, and 2 parents declined consent. The remaining 202 neonates (70.9%) received MR imaging; of those, data from 187 neonates (85 in the hypothermia group and 102 in the control group) were available for analysis. The MR imaging was performed at a median (IQR) age of 15.5 (12.2-24.0) days in the hypothermia group and 14.0 (11.0-19.0) days in the control group.

Among outborn neonates, the mean (SD) absolute levels of thalamic NAA were 8.03 (1.89) mmol/kg wet weight in the hypothermia group and 7.99 (1.72) in the control group (OR, 0.05; 95% CI, −0.62 to 0.71; *P* = .89), and the median (IQR) thalamic lactate to NAA PARs were 0.14 (0.11-0.20) in the hypothermia group and 0.14 (0.10-0.17) in the control group (OR, 1.03; 95% CI, 0.98-1.09; *P* = .18). There was no difference in white matter FA at any brain region between outborn neonates in the hypothermia and control groups (eFigure 3 in Supplement 2). The brain injury scores based on conventional T1- and T2-weighted images in the basal ganglia, white matter, or cerebral cortex did not reveal any differences between the hypothermia and control groups.

### Clinical Outcome Analysis Based on Place of Birth

Clinical outcome analysis included all recruited neonates, irrespective of the MR data. Among the 123 inborn neonates in the hypothermia (n = 62) vs control (n = 61) groups, the rates of gastric bleeding (17 neonates [27.4%] vs 7 [11.5%]), pulmonary hemorrhage (13 neonates [21.0%] vs 4 [6.6%]), coagulopathy (24 neonates [38.7%] vs 13 [21.3.%]), severe thrombocytopenia (10 neonates [16.1%] vs 3 [4.9%]), persistent metabolic acidosis (9 neonates [14.5%] vs 4 [6.6%]), and persistent pulmonary hypertension (11 neonates [17.7%] vs 7 [11.5%]) during neonatal hospitalization and mortality at discharge (24 neonates [38.7%] vs 19 [31.1%]) revealed higher occurrence in the hypothermia group, although these differences were not statistically significant. At 22 months, no statistically significant differences were noted in mortality (25 of 59 neonates [42.4%] vs 20 of 61 [32.8%]; *P* = .28), survival without neurodisability (16 of 33 neonates [48.5%] vs 21 of 40 [52.5%]; *P* = .73), or the composite of death or moderate or severe disability (34 of 58 neonates [58.6%] vs 34 of 60 [56.7%]; RR, 1.03; 95% CI, 0.76-1.41; *P* = .83) among inborn neonates in the hypothermia vs control groups (Table 3).

**Table 3.** Short- and Long-term Outcomes After Whole-Body Hypothermia Among Inborn vs Outborn Neonates

Outcome	Participants, No./total No. (%)		
	Hypothermia group	Control group	RR (95% CI)
**Inborn neonates (n = 123)**			
Total participants, No.	62	61	
Outcomes at hospital discharge			
Gastric bleeding	17/62 (27.4)	7/61 (11.5)	2.39 (1.07-5.35)
Pulmonary hemorrhage	13/62 (21.0)	4/61 (6.6)	3.20 (1.10-9.26)
Major hemorrhage	1/62 (1.6)	1/61 (1.6)	0.98 (0.06-15.30)
Extracranial bleeding	3/62 (4.8)	7/61 (11.5)	0.42 (0.11-1.56)
Prolonged coagulation	24/62 (38.7)	13/61 (21.3)	1.82 (1.02-3.22)
Severe thrombocytopenia	10/62 (16.1)	3/61 (4.9)	3.28 (0.95-11.30)
Persistent metabolic acidosis	9/62 (14.5)	4/61 (6.6)	2.21 (0.72-6.81)
Persistent hypotension	16/62 (25.8)	8/61 (13.1)	1.97 (0.91-4.26)
Persistent pulmonary hypertension	11/62 (17.7)	7/61 (11.5)	1.55 (0.64-3.75)
Culture-positive early-onset sepsis	8/62 (12.9)	4/61 (6.6)	1.97 (0.62-6.20)
Culture-positive late-onset sepsis	2/62 (3.2)	7/61 (11.5)	0.28 (0.06-1.30)
Necrotizing enterocolitis	0	0	NA
Cardiac arrythmia	1/62 (1.6)	0	NA^a^
Kidney failure	7/62 (11.3)	6/61 (9.8)	1.15 (0.41-3.22)
Pneumonia	7/62 (11.3)	9/61 (14.8)	0.76 (0.30-1.92)
Inotropic support in first 4 d	48/62 (77.4)	38/61 (62.3)	1.24 (0.98-1.58)
>1 Inotrope	26/62 (41.9)	18/61 (29.5)	1.42 (0.87-2.31)
Anticonvulsant treatment in first 4 d	47/62 (75.8)	51/61 (83.6)	0.91 (0.76-1.08)
>1 Anticonvulsant	11/62 (17.7)	15/61 (24.6)	0.72 (0.36-1.44)
Death by time of discharge	24/62 (38.7)	19/61 (31.1)	1.24 (0.76-2.02)
Duration of hospitalization, median (IQR), d^b^	14.6 (11.8-21.0)	14.8 (11.4-18.4)	1.04 (0.86-1.27)
Outcomes at age 18-22 mo			
Unavailable for follow-up	3/62 (4.8)	0	NA^a^
Microcephaly	17/33 (51.5)	15/40 (37.5)	1.37 (0.82-2.31)
Survival without disability	16/33 (48.5)	21/40 (52.5)	0.92 (0.58-1.46)
Bayley cognitive composite score <85	10/32 (31.3)	14/39 (35.9)	0.87 (0.45-1.70)
Bayley motor composite score <85	9/32 (28.1)	13/39 (33.3)	0.84 (0.41-1.72)
Bayley language composite score <85	13/32 (40.6)	16/39 (41.0)	0.99 (0.56-1.74)
Cerebral palsy	8/33 (24.2)	13/40 (32.5)	0.75 (0.35-1.58)
Death	25/59 (42.4)	20/61 (32.8)	1.29 (0.81-2.06)
Death or moderate or severe disability (ITT)^c^^,^^d^	34/58 (58.6)	34/60 (56.7)	1.03 (0.76-1.41)
Death or moderate or severe disability (per protocol)^c^^,^^d^	34/58 (58.6)	34/60 (56.7)	1.03 (0.76-1.41)
**Outborn neonates (n = 285)**			
Total participants, No.	140	145	
Outcomes at hospital discharge			
Gastric bleeding	45/140 (32.1)	27/145 (18.6)	1.73 (1.14-2.62)
Pulmonary hemorrhage	29/140 (20.7)	24/145 (16.6)	1.25 (0.77-2.04)
Major hemorrhage	1/140 (0.7)	3/145 (2.1)	0.35 (0.04-3.28)
Extracranial bleeding	4/140 (2.9)	4/145 (2.8)	1.04 (0.26-4.06)
Prolonged coagulation	55/140 (39.3)	39/145 (26.9)	1.46 (1.04-2.05)
Severe thrombocytopenia	23/140 (16.4)	12/145 (8.3)	1.99 (1.03-3.83)
Persistent metabolic acidosis	37/140 (26.4)	20/145 (13.8)	1.92 (1.17-3.13)
Persistent hypotension	29/140 (20.7)	17/145 (11.7)	1.77 (1.02-3.07)
Persistent pulmonary hypertension	13/140 (9.3)	9/145 (6.2)	1.50 (0.66-3.39)
Culture-positive early-onset sepsis	4/140 (2.9)	6/145 (4.1)	0.69 (0.20-2.39)
Culture-positive late-onset sepsis	12/140 (8.6)	4/145 (2.8)	3.11 (1.03-9.40)
Necrotizing enterocolitis	5/140 (3.6)	1/145 (0.7)	5.18 (0.61-43.80)
Cardiac arrythmia	4/140 (2.9)	0	NA^a^
Kidney failure	15/140 (10.7)	10/145 (6.9)	1.55 (0.72-3.34)
Pneumonia	19/140 (13.6)	16/145 (11.0)	1.23 (0.65-2.29)
Inotropic support in first 4 d	113/140 (80.7)	88/145 (60.7)	1.33 (1.14-1.55)
>1 Inotrope	78/140 (55.7)	52/145 (35.9)	1.55 (1.19-2.02)
Anticonvulsant treatment in first 4 d	127/140 (90.7)	132/145 (91.0)	1.00 (0.93-1.07)
>1 Anticonvulsant	21/140 (15.0)	31/145 (21.4)	0.70 (0.42-1.16)
Death by time of discharge	48/140 (34.3)	30/145 (20.7)	1.66 (1.12-2.45)
Duration of hospitalization, median (IQR), d^e^	17.0 (13.1-23.3)	13.7 (10.3-18.8)	1.24 (1.07-1.44)
Outcomes at age 18-22 mo			
Unavailable for follow-up	1/140 (0.7)	5/145 (3.4)	0.21 (0.02-1.75)
Microcephaly	16/77 (20.8)	22/95 (23.2)	0.90 (0.51-1.59)
Survival without disability	31/78 (39.7)	26/96 (27.1)	1.48 (0.96-2.25)
Bayley cognitive composite score <85	26/76 (34.2)	32/94 (34.0)	1.00 (0.66-1.53)
Bayley motor composite score <85	8/76 (10.5)	20/94 (21.3)	0.49 (0.23-1.06)
Bayley language composite score <85	34/76 (44.7)	58/94 (61.7)	0.73 (0.54-0.98)
Cerebral palsy	4/78 (5.1)	15/96 (15.6)	0.33 (0.11-0.95)
Death	59/139 (42.4)	43/140 (30.7)	1.38 (1.01-1.89)
Death or moderate or severe disability (ITT)^c^^,^^f^	64/137 (46.7)	60/139 (43.2)	1.08 (0.83-1.41)
Death or moderate or severe disability (per protocol)^c^^,^^g^	64/136 (47.1)	60/139 (43.2)	1.09 (0.84-1.42)
Death or moderate or severe disability (ITT)^h^^,^^i^	61/133 (45.9)	55/133 (41.4)	1.11 (0.84-1.46)
Death or moderate or severe disability (per protocol)^h^^,^^j^	61/132 (46.2)	55/133 (41.4)	1.12 (0.85-1.47)

Abbreviations: ITT, intention to treat; NA, not applicable; RR, risk ratio.

^a^ Unable to calculate RRs due to no occurrence of outcome in 1 group.

^b^ Among 38 participants in the hypothermia group and 41 in the control group.

^c^ Severe disability was defined as any 1 of the following: a cognitive composite score of less than 70 on the Bayley Scales of Infant and Toddler Development, Third Edition (Bayley-III)^14^; a gross motor function classification system level of 3 to 5; a profound hearing impairment requiring hearing aids or a cochlear implant; or blindness. Moderate disability was defined as a cognitive composite score of 70 to 84 on the Bayley-III and 1 or more of the following: a gross motor function classification system level of 2, a hearing impairment with no amplification, or a persistent seizure disorder.

^d^ *P* = .83.

^e^ Among 92 participants in the hypothermia group and 115 in the control group.

^f^ *P* = .55.

^g^ *P* = .52.

^h^ Excluding neonates who were born at home.

^i^ *P* = .46.

^j^ *P* = .43.

**Figure 2.**
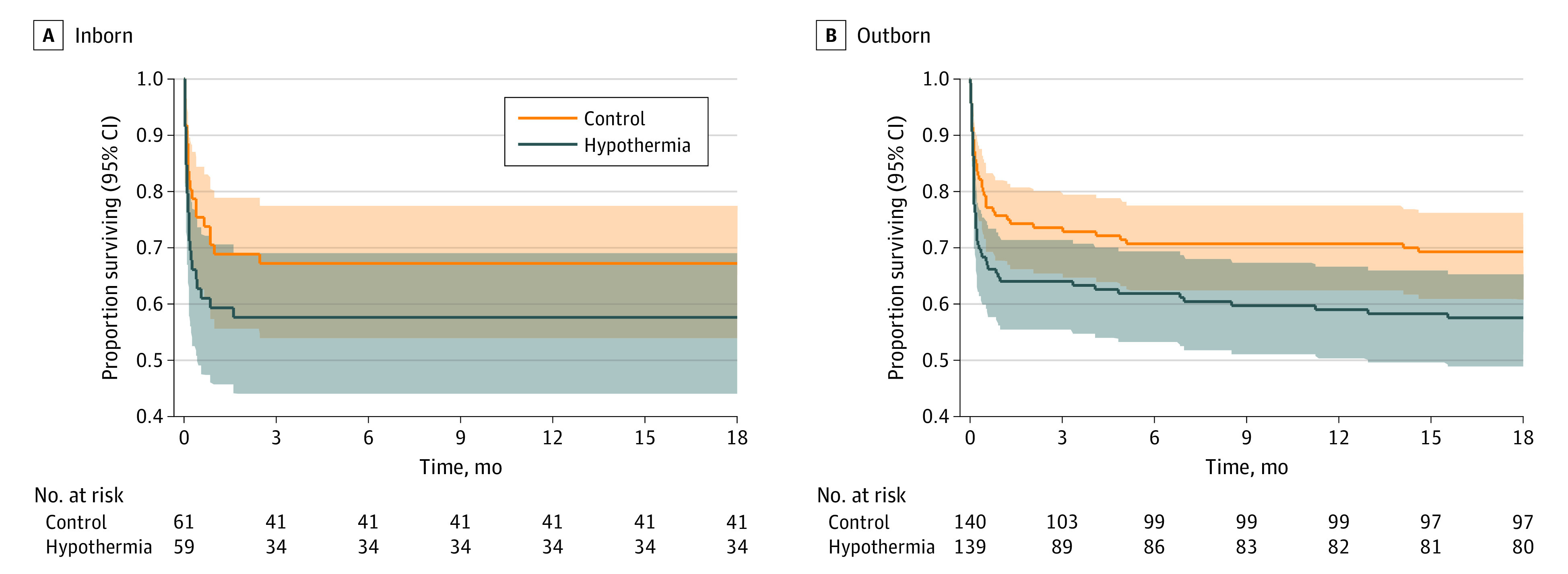
Kaplan-Meier Survival Curves for Inborn and Outborn Neonates

Among the 285 outborn neonates in the hypothermia (n = 140) vs control (n = 145) groups, the rates of gastric bleeding (45 neonates [32.1%] vs 27 [18.6%]; *P* = .01), coagulopathy (55 neonates [39.3%] vs 39 [26.9%]; *P* = .03), severe thrombocytopenia (23 neonates [16.4%] vs 12 [8.3%]; *P* = .04), persistent metabolic acidosis (37 neonates [26.4%] vs 20 [13.8%]; *P* = .01), and inotropic use (113 neonates [80.7%] vs 88 [60.7%]; *P* = .001) during neonatal hospitalization mortality at hospital discharge (48 neonates [34.3%] vs 30 [20.7%]; *P* = .01) and at 22 months (59 of 139 neonates [42.4%] vs 43 of 140 [30.7%]; *P* = .04) were significantly higher in the hypothermia group (Table 3 and Figure 2). Conversely, neonates in the hypothermia group had better rates of survival without neurodisability than those in the control group (31 of 78 neonates [39.7%] vs 26 of 96 [27.1%]; *P* = .08), although this difference was not statistically significant. The composite outcome of death or moderate or severe disability was not significantly different in the hypothermia vs control groups (64 of 137 neonates [46.7%] vs 60 of 139 [43.2%]; RR, 1.08; 95% CI, 0.83-1.41; *P* = .55). The results were similar when 10 neonates who were born at home were excluded from the analysis (Table 3).

Whole-body hypothermia was associated with increased mortality (RR, 1.35; 95% CI, 1.04-1.76; number needed to harm, 9) and reduced cerebral palsy (RR, 0.53; 95% CI, 0.28-0.98; number needed to treat, 10) at 18 to 22 months among all 408 neonates recruited to the HELIX trial. Among inborn neonates, the relative risk of mortality was 29% higher (RR, 1.29; 95% CI, 0.81-2.06; *P* = .28) and cerebral palsy was 25% lower (RR, 0.75; 95% CI, 0.35-1.58; *P* = .44) in the hypothermia group than the control group, although these differences were not statistically significant. Among outborn neonates, the relative risk of mortality was 38% higher (RR, 1.38; 95% CI, 1.01-1.89; *P* = .01) and cerebral palsy was 67% lower (RR, 0.33; 95% CI, 0.11-0.95; *P* = .05) in the hypothermia group than the control group.

## Discussion

This nested cohort study involved a subgroup analysis of data from the HELIX trial, which was, to our knowledge, the largest clinical trial of hypothermia reported to date and the first to use quantitative MR biomarkers in a multicountry setting using harmonized cross-platform sequences. Findings revealed that whole-body hypothermia was not associated with reductions in brain injury measured by thalamic NAA levels, thalamic lactate to NAA or NAA to creatinine PARs, tract-based spatial statistics of whole-brain white matter FA, or conventional MR imaging injury scores, irrespective of place of birth. The direction of treatment effect (or lack of treatment effect) measured using thalamic NAA levels, thalamic lactate to NAA PARs, and NAA to creatinine PARs was associated with the clinical outcomes. Whole-body hypothermia was not associated with reductions in death or disability at 18 to 22 months among both inborn and outborn neonates. Because the HELIX trial was adequately powered to assess the clinical outcomes, these data provide the first validation, to our knowledge, of MR spectroscopic biomarkers as a surrogate end point in HIE neuroprotection trials.

Although earlier clinical trials of whole-body hypothermia have reported MR imaging data obtained as part of standard clinical care,^13,15,16^ none used MR spectroscopy or DTI. While MR imaging scoring systems do estimate later outcomes,^17^ they are subjective and user dependent. The Total Body Hypothermia Plus Xenon (TOBY-Xe) trial^18^ acquired MR spectroscopic biomarkers from 78 neonates with HIE at 3 UK sites using Philips Achieva 3T (Philips Medical Systems) MR scanners. The trial found no change in thalamic lactate to NAA PARs with inhaled xenon. The TOBY-Xe trial was not powered to examine clinical outcomes nor did it perform NAA analysis.^18^

The first Eunice Kennedy Shriver National Institute of Child Health and Human Development (NICHD) Neonatal Research Network (NRN) hypothermia trial^3^ included 45% outborn neonates, the Infant Cooling Evaluation (ICE) trial^19^ included 61% outborn neonates, and the more recent High-Dose Erythropoietin for Asphyxia and Encephalopathy (HEAL) trial^20^ included 83% outborn neonates. In the NICHD NRN trial,^5^ neonates born outside an NRN cooling center were more unwell, required longer resuscitation at birth, and more often had severe encephalopathy than inborn neonates. In contrast, the outborn neonates recruited to the HELIX trial were less unwell than the inborn neonates, and many did not require endotracheal intubation at birth. One possible explanation is that outborn neonates requiring extensive resuscitation died soon after birth or were too unwell to be transferred to a tertiary cooling center within 6 hours and hence were not captured in the trial.

An inverse association was seen between mortality and cerebral palsy at 18 to 22 months. For every 10 neonates treated with whole-body hypothermia, at least 1 additional neonate died and 1 case of cerebral palsy was averted. Therefore, it is important that the primary outcome of any neuroprotective intervention for HIE, particularly in NICUs in LMICs, remains the composite of death or moderate or severe disability, and reduction in cerebral palsy should always be considered in the context of shifts in mortality within the same trial and not in isolation.

Although many observational studies from LMICs primarily recruiting neonates with mild brain injury^21,22^ or normal amplitude–integrated electroencephalography^23^ have reported short-term benefits, no inferences about the safety or efficacy of hypothermia can be made because these studies did not include randomized control groups.^21,23,24^ While more than 15 small single-center pilot RCTs have reported short-term benefits of whole-body hypothermia among neonates in LMICs,^25^ the HELIX trial is, to our knowledge, the only well-designed and rigorously conducted multicenter trial to report neurodevelopmental outcomes. Small single-center studies overestimate treatment effects; hence, the inclusion of pilot RCTs in meta-analyses may result in inaccurate estimates.^26^ Moreover, many pilot RCTs are difficult to interpret. For example, 6 trials^27,28,29,30,31,32^ with different event rates but overlapping recruitment periods were reported from the same hospital, 1 trial^33^ reported outcomes at 8 years within a year of completing neonatal recruitment, and another trial^34^ reported a 68% reduction in adverse outcomes using ice cubes on the neonate’s head.

It is unlikely that the lack of hypothermic neuroprotection seen in the HELIX trial^35^ can be explained by (1) delay in achieving target temperature, (2) use of simplified inclusion criteria for neonates born at home (ie, absence of cry by 5 minutes), (3) coexistent infection, or (4) lack of 1:1 nursing care. First, of all the major induced hypothermia trials, age at randomization (4.3 hours) was shortest in the HELIX trial (compared with age 4.8 hours in the Selective Head Cooling With Mild Systemic Hypothermia After Neonatal Encephalopathy [CoolCap] multicenter RCT,^2^ age 4.3 hours in the NICHD NRN trial,^3^ and age 4.7 hours in the Total Body Hypothermia [TOBY] trial^4^), and the cooling device was the most efficient. Thus, 72% of the neonates reached the target temperature (≤34 °C) by 6 hours in the HELIX trial compared with 53% in the NICHD NRN trial.^3^ In the TOBY trial,^4^ target temperatures were achieved much later because 68% of neonates were randomized after 4 hours, although randomization age (<4 hours vs 4-6 hours) had no effect on neuroprotection.

Second, the HELIX trial results did not change when the 10 neonates born at home were excluded. While the inclusion criteria of the HELIX trial (which required resuscitation at birth) were less stringent than those of the NICHD NRN trial^3^ (which required acidosis and resuscitation at birth), they were more stringent than other trials in HICs that required only acidosis or resuscitation at birth and not necessarily both.^2,4^ The NICHD NRN trial^3^ and the HELIX trial were the only clinical trials to perform standardized neurological assessments. Third, the occurrence of coexistent infection and funisitis in the HELIX trial was similar to previous trials conducted in HICs.^2,3,4^ Furthermore, observational studies^36,37^ in LMICs using both polymerase chain reaction testing and blood cultures have reported coexistent infection in fewer than 10% of the neonates with encephalopathy^37^; these findings are again similar to studies in HICs.^2,3,4,36^ Hence, preclinical models of combined infection and ischemia may not represent HIE scenarios in LMICs. Fourth, the HELIX trial was conducted in accredited tertiary NICUs managed by dedicated neonatologists, mostly with 1:2 to 1:3 nursing care ratios. All previous pilot trials in LMICs had neonate to nurse ratios of 1:4^38^ or worse.^24^ Any intervention that requires 1:1 nursing care and is unsafe outside a tertiary NICU setting has no generalizability in LMICs. For example, the highest burden of HIE in LMICs occurs in secondary (district-level) care neonatal units with neonate to nurse ratios of 1:15 to 1:30.^39^

One possible explanation for the lack of hypothermic neuroprotection is difference in the nature and timing of intrapartum hypoxia. In preclinical models, hypothermia was associated with the prevention of secondary energy failure only when the hypoxic injury was single, acute, and occurred in a previously healthy animal^40^; this scenario closely mimicked acute intrapartum sentinel events among individuals in HICs. Because we did not have detailed intrapartum monitoring data, no definite conclusions about the timing of fetal brain injury can be made. Nevertheless, a constellation of factors comprising infrequent major intrapartum sentinel events, low birth weight, coagulopathy, early-onset clinical seizures, and cerebral white matter injury suggests that the unborn neonates may have already been compromised (eg, due to suboptimal nutrition and other socioeconomic factors). Hence, brain injury might occur from intermittent cerebral hypoxia during active labor, especially if labor is medically augmented^41^—“vulnerable baby in the womb.” Preclinical models of growth-restricted animals and intermittent umbilical cord occlusion^42^ may represent this scenario. If confirmed by subsequent mechanistic studies, this hypothesis may lead to a paradigm shift in our understanding of HIE in LMICs and aid in the development of appropriate neuroprotective therapies.

### Limitations

This study has several limitations. As with all previous studies,^13,15,16^ we were unable to obtain MR imaging in neonates who died soon after birth. The mortality rate among neonates who had MR imaging was 8.8%, while those who did not have MR imaging was 98.4%; therefore, deep brain nuclei and brainstem injury might have been underreported. Furthermore, the HELIX trial recruited only populations in LMICs with low income and high HIE burden and did not recruit those receiving care from private hospitals. Private-sector hospitals primarily cater to the middle and upper socioeconomic classes, have small maternity units with fewer than 2000 births per year, have substantially more neonates who are born by elective cesarean than vaginal delivery, and thus have very few inborn neonates with HIE.^43^ Nevertheless, interventions in HICs may not directly translate to those settings; for example, the survival of premature neonates is substantially lower and the occurrence of neonatal sepsis is 19 times higher (1.7% vs 32.0%) in even well-resourced private-sector tertiary NICUs in India than public-sector NICUs in the UK.^44^

## Conclusions

In this nested cohort study within the HELIX trial, whole-body hypothermia was not associated with reductions in brain injury as measured by quantitative MR biomarkers, irrespective of place of birth, among neonates in South Asian NICUs. Quantitative MR spectroscopic biomarkers can be used as surrogate outcome measures for future clinical trials and for further comparison between populations in HICs and LMICs. Policy makers should be aware that continued use of whole-body hypothermia may harm the population with the highest disease burden and worsen inequalities in LMICs.

## Notes

Supplement 1.
HELIX Trial Protocol

Supplement 2. **eTable 1.** Baseline Characteristics of Inborn and Outborn Neonates **eTable 2.** Clinical Characteristics of Neonates With and Without MR Imaging (MRI) **eFigure 1.** Flowchart **eFigure 2.** Mean (SD) Rectal Temperature of Inborn (Top Panel) and Outborn (Bottom Panel) Neonates **eFigure 3.** Treatment Effects of Whole-Body Hypothermia on Whole-Brain White Matter Fractional Anisotropy Quantified by Tract-Based Spatial Statistics Among Inborn (A) and Outborn (B) Neonates

Supplement 3.
Data Sharing Statement
