# Supplementary material for: Whole-Body Hypothermia, Cerebral Magnetic Resonance Biomarkers, and Outcomes in Neonates With Moderate or Severe Hypoxic-Ischemic Encephalopathy Born at Tertiary Care Centers vs Other Facilities: A Nested Study Within a Randomized Clinical Trial
Source: JAMA Netw Open. 2023 May 8;6(5):e2312152. doi: 10.1001/jamanetworkopen.2023.12152 (PMC10167567; doi:10.1001/jamanetworkopen.2023.12152)
Supplement: Supplement 1. — HELIX Trial Protocol [file jamanetwopen-e2312152-s001.pdf]

Protocol

This trial protocol has been provided by the authors to give readers additional information about their work.

Protocol for: *Thayyil S, Pant S, Montaldo P, et al. Hypothermia for moderate or severe neonatal encephalopathy in low and middle-income countries (HELIX) trial*

Hypothermia for moderate or severe neonatal encephalopathy in low and middle-income countries

<u>Study Protocol, Analysis Plans and the Independent Data Monitoring Committee Charter</u>

This supplement contains the following items:

1. Original version of the study protocol (Version 1.1)…………………………………3 to 41
2. Peer review of the study protocol ……………………………………………….……….42 to 48
3. Final version of the study protocol (Version 3.1)…………………………….………49 to 61
4. Summary of the changes in the protocol ……………………………………………..62
5. Original version of the statistical analysis plan (Version 1.2)………………………63 to 74
6. Final version of the statistical analysis plan (Version 1.3)…………………………..75 to 84
7. Summary of the changes to the statistical analysis plan………………………….……85
8. Independent Data Monitoring Committee Charter (Version 1). …………………….86 to 99
9. Summary of changes to the Independent Data Monitoring Committee Charter ...100

Clinical Trial Protocol

Trial Title:	**Hypothermia for Encephalopathy in Low and Middle-Income Countries (HELIX) trial**
Protocol Number:	
Clinical trials.gov number:	NCT02387385
Protocol Version:	HELIX Protocol Version 1.1

Chief Investigator: Dr Sudhin Thayyil
Weston Reader and Hon Consultant Neonatologist
Director, Centre for Perinatal Neuroscience
Department of Paediatrics
Imperial College London
Du Cane Road
London W12 0HS
Telephone: +44 203 313 8515

Trial Sponsor: Imperial College London, London

Type of study: Non-CTIMP (Controlled Trial of Investigational Medical Product), Un-blinded Randomised controlled trial

CONTENTS

1 Protocol signatures 5
2 Abbreviations 6
3 Trial synopsis 7
4 Introduction 8
4.1 Background 8
5 Trial design 13
5.1 Trial objectives 13
5.2 Trial outcome measures 14
Primary outcome measure 14
Secondary outcome measures 14
6 Selection and withdrawal of subjects 14
6.1 Inclusion criteria 14
6.2 Exclusion criteria 15
6.3 Screening evaluation and neurological examination 15
6.4 Treatment assignment and randomisation 16
6.5 Method of blinding 16
6.6 Cooling therapy 16
6.7 Criteria for stopping cooling therapy 17
7 Supportive care and monitoring 17
7.1 Baseline assessments and data collection 17
7.2 Exploratory sub studies 18
8 Adverse events 19
9. Statistical methods 20
10. Trial organisation 21
a. Independent Data Monitoring Committee (IDMC) 21
b. Trial Steering Committee 21
c. Project Management Group 22
11. Ethical and regulatory considerations 22
a. Consent 22
b. Ethical committee review 22
c. Declaration of Helsinki and Good Clinical Practice 23
d. Data protection and patient confidentiality 23
e. GCP Training 23
12. Sponsorship, Financial and Insurance 23
13. Monitoring, Audit & Inspection 23
14. Protocol Compliance and Breaches of GCP 23
15. Publications policy 24
16. Appendices 24

1 Protocol signatures

Chief Investigator

I give my approval for the attached protocol entitled “Hypothermia for Encephalopathy in Low and Middle-Income Countries (HELIX) trial” Version 1.1 dated 19.3.2015.

Name: Dr Sudhin Thayyil

Signature: ____________________________________

Date: 19.3.2015___________________

Site Principal Investigator statement of compliance

I have read the attached protocol entitled “Hypothermia for Encephalopathy in Low and Middle-Income Countries (HELIX) trial” Version 1.1 dated 19.3.2015, and agree to abide by all provisions set forth therein.

I agree to comply with the conditions and principles of Good Clinical Practice as outlined in the European Clinical Trials Directives 2001/20/EC and the GCP Directive 2005/28/EC, and will ensure all local (Indian) regulatory approvals are obtained prior to the case recruitment.

I agree to ensure that the confidential information contained in this document will not be used for any other purpose other than the evaluation or conduct of the clinical investigation without the prior written consent of the Sponsor.

Name:

Signature: ____

Date: ____________________________

2 Abbreviations

CPN	Centre for Perinatal Neuroscience
CRF	Case Report Form
CTIMP	Controlled trial of an Investigational Medicinal Product
DSUR	Development Safety Update Report
GCP	Good Clinical Practice
ICL	Imperial College London
IDMC	Independent Data Monitoring Committee
LMIC	Low and middle-income countries
MHRA	Medicines and Healthcare products Regulatory Agency
MMC	Madras Medical College
NICHD	National Institute of Child Health and Human Development
NIMP	Non Investigational Medicinal Product
R&D	Research and Development
RA	Regulatory Agency
REC	Research Ethics Committee
SAE	Serious Adverse Event
SUSAR	Suspected Unexpected Serious Adverse Reaction
TMG	Trial Management Group
TSC	Trial Steering Committee

3 Trial synopsis

Title of clinical trial	Hypothermia for Encephalopathy in Low and Middle-Income Countries (HELIX) trial
Sponsor name	Imperial College London
Clinical trials number	
Medical condition or disease under investigation	Neonatal Encephalopathy
Primary objective	To examine whether whole body cooling to 33.5^{0}C initiated within 6 hours of birth and continued for 72 hours reduces death or neurodisability at 18 months after neonatal encephalopathy in low and middle-income countries.
Trial Design	Un blinded, pragmatic randomised controlled trial
Trial Outcome Measures	Death or moderate or severe neurodisability
Sample Size	408
Summary of eligibility criteria	Inclusion Criteria<br>1. Age < 6 hours, Birth-weight >1.8 kg, Gestation $\geq$36 weeks<br>2. Need for continued resuscitation at 5 minutes after birth and/or 5 minute Apgar score <6 (in babies born at hospital) or lack of cry by 5 minutes of age (for babies born at home)<br>3. Evidence of moderate or severe encephalopathy on clinical examination within 6 hours of age.<br><br>Exclusion Criteria<br>1. Absent heart rate at 10 minutes of age despite adequate resuscitation.<br>2. Major life threatening congenital malformation.<br>3. Migrant family or parents unable/unlikely to come back for follow up at 18 months.<br>4. Lack of parental consent.
Intervention	Whole body cooling to 33^{0}C to 34^{0}C
Maximum duration of treatment of a subject	72 hours
Procedures: Screening & enrolment	Detailed neurological examination as per the NICHD encephalopathy criteria within 6 hours of birth
Treatment period	Whole body cooling for 72 hours
End of Trial	Neurological outcome assessment at 18 months
Criteria for withdrawal of patients	Withdrawal of parent or physician consent

4 Introduction

4.1 Background

Every year, approximately one million babies die from 'neonatal encephalopathy' in low and middle-income countries (LMIC) – a condition arising from unexpected lack of cerebral blood flow and oxygen supply to the fetal brain at the time of birth– a quarter of these deaths occur in India[1]

Approximately a third of infants with moderate or severe encephalopathy will die during the newborn period, and up to three quarters of survivors develop long-term neurodisability[2,3]. Until recently there was no effective treatment for this condition, and the management was limited to supportive care.

4.2 Cooling therapy in high-income countries

A number of high quality cooling trials have been conducted in high-income countries in the past decade[4-6]. The meta-analyses of these trials have convincingly demonstrated that selective head or whole body cooling along with optimal tertiary intensive care reduce mortality (risk ratio (RR) 0.8; 95% confidence interval (CI) 0.7 to 0.9; p=0.005), and improve survival with normal neurological outcome (RR 1.5; 95% CI 1.2, 1.9; p<0.001) after neonatal encephalopathy in these settings[7,8]. The protective effect of cooling persists into later childhood[9]. Whole body cooling is now widely used as a standard therapy for encephalopathy in the UK and other high-income countries.

4.3 Adverse effects of cooling therapy

When cooling therapy is offered under optimal tertiary intensive care, the only significant adverse effect reported is transient thrombocytopenia and an increased requirement of platelet transfusions (risk difference 0.06; 95% CI 0.01 to 0.1). Subcutaneous fat necrosis occurred in approximately 1% of babies[7]. A summary of the adverse effects reported from major high-income country cooling trials is given below.

Figure 1. Adverse outcomes reported in cooling trials conducted in high-income countries.

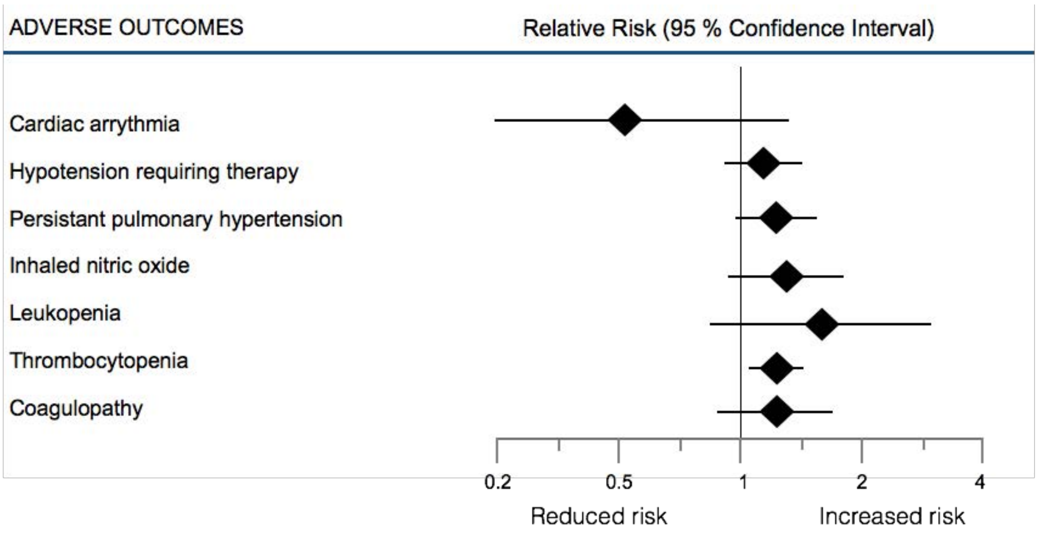

Recently published short-term outcome data from the optimising cooling trial suggest that deeper cooling (to 32^{0}C) or prolonged cooling (for 120 hours) do not increase neuroprotection[10]. On the contrary, deeper cooling to 32^{0}C increased the adverse outcomes of cooling therapy including worsening of pulmonary hypertension requiring nitric oxide therapy and extracorporeal membrane oxygenator support, bradyarrythmia and bleeding. Thus adherence to the target

therapeutic range and duration i.e. core body temperature of 33.5^{0}C (33^{0}C to 34^{0}C) for 72 hours is vital for optimal neuroprotection with minimal adverse events[10].

4.4 Rationale for the proposed trial

Although the burden of neonatal encephalopathy is far higher in low and middle-income countries, the safety and efficacy data on cooling therapy from high income cooling trials cannot be extrapolated to these settings[11,12].

Firstly, all high-income country clinical trials to date have provided cooling therapy along side optimal tertiary neonatal intensive care and cardiorespiratory support. Such tertiary care includes 1:1 expert nursing care, continuous clinical monitoring of vital physiological parameters, close attention to acid base and electrolyte balance, optimal ventilatory and inotropic support, parenteral nutrition, nitric oxide and cerebral function monitoring. These centres also have access to extra-corporeal membrane oxygenation (ECMO) facilities for infants with persistent pulmonary hypertension and meconium aspiration that may be adversely affected by cooling.

The safety and efficacy of cooling therapy without optimal tertiary neonatal intensive care is unknown. Even the best resourced public sector tertiary neonatal units in India and other low and middle-income countries do not have the facilities and expertise that are comparable to the neonatal units where the high-income cooling trials were originally performed. The dangers of extrapolating the safety and efficacy data from high income country intensive care units to low and middle-income countries is well known, and has been recently re-emphasised by the increased mortality seen after fluid boluses in children with septic shock in Africa (FEAST trial)[13].

The HELIX trial will examine the safety of cooling therapy in under resourced public sector neonatal units in India, who do not have the above-mentioned facilities for providing optimal tertiary intensive care, alongside cooling therapy.

Secondly, there are significant population differences in babies who suffer from encephalopathy in high-income countries and those in low and middle-income countries with a higher incidence of perinatal infection and meconium aspiration. Antenatal care is often poor, and intra uterine growth restriction and delayed hospital admission in obstructed labour are extremely common. Thus the brain injury may be more chronic and already established, such that the window period for cooling may be already lost by the time baby is born.

Two recent NICHD hypothermia workshops (2011 and 2013)[14] involving experts in therapeutic hypothermia have recommended that rigorous evaluation of cooling therapy should be urgently conducted in LMIC, to ensure that the benefits of one of the most important discoveries in neonatal medicine are not lost to the population that needs it most. Without such rigorous evaluation there may be a creeping introduction of cooling therapy, which is constantly sabotaged by residual safety concerns, and it will never be widely used in India and other LMIC.

<u>Cooling studies from low and middle-income countries</u>

A number of small randomised controlled trials have been reported from low and middle-income countries. Individual studies were small and of poor quality. The largest of these trials reported from China had excluded babies at risk of perinatal sepsis and had substantial methodological concerns[15]. Two studies reported increased mortality with cooling[16,17]. Meta-analysis of all these trials showed a trend towards reduced mortality, however this was not statistically significant (RR 0.74; 95% CI 0.4 to 1.3) (Figure 2)[18]. More importantly, the confidence intervals were wide and therefore significant benefits or harm cannot be excluded. There were no data on long term neurological follow up after cooling therapy.

Figure 2. Neonatal mortality following therapeutic hypothermia in pilot randomised controlled trials in low and middle-income countries

	Cooled		Control		Risk Ratio	
Study or Subgroup	Events	Total	Events	Total	M-H, Random, 95% CI	Year
Akisu 2003	0	11	2	10	0.18 [0.01, 3.41]	2003
Lin 2006	2	32	2	30	0.94 [0.14, 6.24]	2006
Bhatt 2006	3	20	5	15	0.45 [0.13, 1.59]	2006
Robertson 2009	7	21	1	15	5.00 [0.69, 36.50]	2009
Zhou 2010	20	138	27	118	0.63 [0.38, 1.07]	2010
Thayyil 2012	4	17	2	16	1.88 [0.40, 8.90]	2012
Bharadwaj 2012	3	62	6	62	0.50 [0.13, 1.91]	2012
**Total (95% CI)**		**301**		**266**	**0.74 [0.44, 1.25]**	
Total events	39		45			

Heterogeneity: $Tau^2 = 0.09$; $Chi^2 = 7.14$, df = 6 (P = 0.31); $I^2 = 16\%$
Test for overall effect: Z = 1.13 (P = 0.26)

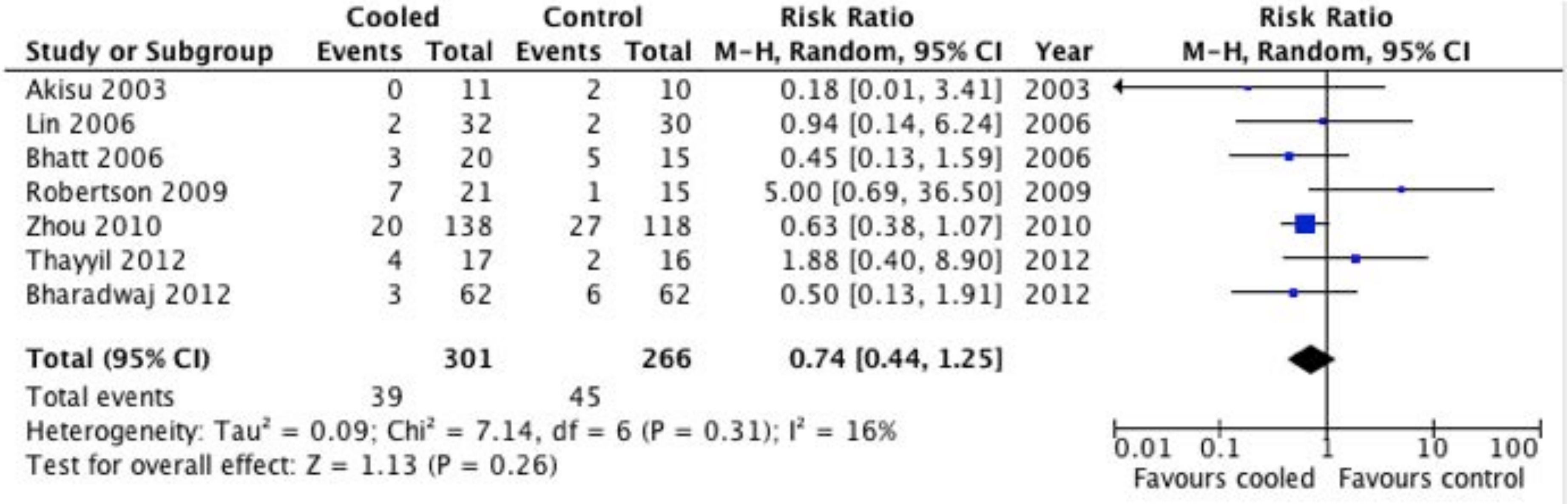

This meta-analysis concluded that further data from robust randomised controlled trials in low and middle-income countries are required before it is considered as a standard therapy in these settings.

Recently, a number of small observational studies on cooling have reported very low mortality (<5%) in cooled babies in low and middle-income countries, which is likely to be due to inclusion of babies with perinatal asphyxia without encephalopathy or with mild encephalopathy, rather the therapeutic effect of cooling (Table 1). This is a very worrying trend as it might be unnecessarily exposing babies who would otherwise have a normal outcome, to the possible adverse effects of cooling. The extremely low neonatal mortality in studies claiming to have recruited only infants with moderate or severe neonatal encephalopathy may be due to inadequate standardisation of the neurological examination, and poor research governance. Use of blood gas criteria appears to have little effect in selecting high-risk infants in these studies (manuscript in preparation)

Thus the existing evidence on safety and efficacy of cooling therapy in low and middle-income countries is extremely poor, and significant

Study	No of deaths/total babies in standard care arm, if applicable (%)	No of deaths/total babies cooled (%)	Cooling device	Criteria used to define encephalopathy	Grade of encephalopathy: Mild/moderate/severe	Blood gas criteria used	N
**Group A: Neonatal mortality ≤5%**							
Akisu (2003)	2/10 (20%)	0/11 **(0%)**	Water cooling caps	Modified Sarnat Staging	0/12/6	Yes	21
Magalhães (2013)	NA (case series)	0/35 **(0%)**	Ice Packs	Clinical examination	Moderate to severe	Yes	35
Thomas (2011)	NA (case series)	1/20 **(5%)**	Frozen gel packs	Modified Sarnat criteria	0/16/4	Yes	20
Bharadwaj (2012)	6/62 (10%)	3/62 **(5%)**	Frozen gel packs	Modified Sarnat Staging	0/109/15	Yes	124
Joy (2013)	4/58 (7%)	2/58 **(2%)**	Frozen gel packs	Modified Sarnat criteria	0/100/16	Yes	116
LI (2009)	3/44 (7%)	1/38 **(3%)**	Not described	Sarnat and Sarnat staging	Moderate to severe	Yes	82
**Group B: Neonatal mortality 5% to 20%**							
Gane (2014)	8/50(14%)	4/53 **(7%)**	Frozen gel packs	Unclear	0/89/31	Yes	120
Lin (2006)	2/30 (6%)	2/32 **(6%)**	Water cooling caps	Modified Sarnat Staging	14/31/17	Yes	62
See (2010)	NA	3/17 **(12%)**	Ambient temp	Sarnat and Sarnat staging	0/15/2	No	17
Horn (2009)	NA	1/10 **(10%)**	Servo controlled fans	Sarnat and Sarnat staging	0/6/4	Yes	10
Bhatt (2006)	5/15 (33%)	3/20 **(15%)**	Unclear	Unclear	Unclear	Yes	35
**Group C: Neonatal mortality >20%**							
Horn (2011)	NA	3/14 **(21%)**	Frozen gel packs	Sarnat and Sarnat staging	Moderate to severe	No	14
Thayyil (2013)	2/16 (12.5%)	4/17 **(24%)**	Phase changing material	Thompson score >5	18/11/4	No	33
Zhou (2010)	46/118 (39%)	31/138 **(22%)**	Water cooling caps	Modified Sarnat Staging	39/82/73	Yes	256
Robertson (2008)	1/15 (7%)	7/21 **(33%)**	Water bottles	Thompson score >5	9/20/7	No	36

NA = Not applicable

benefits or harm with cooling therapy cannot be excluded, emphasising the need for large high quality randomised controlled trials in these settings[12].

Table 1. Variation in neonatal mortality reported in cooling studies (case series and pilot randomised controlled trials) from low and middle-income countries.

4.5 Preparatory work for the HELIX trial
In preparation for the HELIX trial, we conducted a feasibility study of cooling therapy using a servo controlled cooling device (Tecotherm) in 2013/14, at Madras Medical College (MMC), Chennai. The data from 58 cooled babies with neonatal encephalopathy and 112 contemporary 'un matched' encephalopathic babies who had usual care (normothermia) admitted to the neonatal unit over a 15 month period are given below (Table 2).

Table 2. Population characteristics and short term adverse outcomes after neonatal encephalopathy at Madras Medical College, Chennai.

	Cooled (n=58)	Usual care (n=112)
Mean birth weight (g)	2948 (319)	2860 (353)
Mean gestational age (weeks)	39.2 (0.9)	39.2 (0.9)
Severity of encephalopathy at < 6 hour of age		
Moderate	37 (64%)	64 (57%)
Severe	21 (36%)	48 (43%)
Age at start of cooling (hours)	4.7 (2.1)	NA
Temperature at start of cooling (^{0}C)	36.1 (1.1)	NA
Temperature during cooling (^{0}C)	33.5 (0.1)	NA
Induction time (hours)	1.2 (0.6)	NA
Effective cooling time (%)	98.8% (3.2%)	NA
Re-warming rate (^{0}C/hour)	0.4 (0.1)	NA
Overall neonatal mortality	17 (29%)	60 (54%)
Mortality in moderate encephalopathy	4/37 (11%)	14/64 (22%)
Mortality in severe encephalopathy	13/21 (62%)	38/48 (79%)
Seizures	55 (95%)	108 (95%)
Invasive ventilation	26 (52%)	44 (39%)
Hypotension requiring inotropic therapy	47 (94%)	98 (88%)
Thrombocytopenia	35 (60%)	78 (70%)
Gastric bleeding	31 (53%)	36 (32%)
Persistent metabolic acidosis	17 (34%)	32 (27%)
Shivering	21 (42%)	0
Subcutaneous fat necrosis	1 (2%)	0

Although the neonatal mortality in the cooled babies appears to be higher than the high-income country cooling trials, it was lower than the contemporary encephalopathic population at this hospital. High incidence of gastric bleeds and persistent metabolic acidosis were seen in both groups. The cases and controls were not matched and it is possible that the control babies were more unwell, and therefore had higher mortality (29% versus 54%; Table 1); nevertheless, these data are reassuring and support further evaluation of cooling therapy in a rigorous randomised controlled trial (manuscript in preparation).

4.6. Generalisability of the HELIX trial results

A paradox of private and public sector health care exists in India and other low and middle-income countries. Private hospitals are often very well equipped and have good tertiary intensive care facilities, but are not affordable to the low income populations. Moreover, these hospitals are relatively smaller (annual delivery rates of less than 2000), and have a low encephalopathy burden. Public sector hospitals on the other hand, tend to be much larger (20, 000 to 30,000 deliveries per year) and offer free health care to the low income population.

These hospitals have a huge encephalopathy burden (both inborn and out-born babies), but lack resources and good neonatal intensive care facilities. Thus, significant health benefits will occur only if the neuroprotective therapies are usable and indeed effective in public sector hospitals in the low and middle-income countries, rather than in private health care.

The HELIX trial is carefully designed to be generalizable to all under-resourced neonatal units with sub optimal neonatal intensive in India and other low and middle-income countries, that bear a very high neonatal encephalopathy burden. However, the neonatal units need to have a good quality basic neonatal care, including facilities of neonatal resuscitation, administration of intravenous fluids, drugs and basic respiratory support, but not optimal cardio respiratory or 1:1 nursing care facilities.

The HELIX trial will be conducted in the real life situation of under resourced public sector neonatal units lacking optimal tertiary intensive care in India. Exclusively clinical criteria will be used for case identification and recruitment, and no laboratory parameters, neuroimaging or cerebral function monitoring will be required for eligibility. The entire cooling therapy will be provided by the existing clinical teams, and the research team will only be involved in accurate and high quality data collection, so that the trial results are reflective of the routine clinical scenario.

Clearly, the population co-morbidities and resources in Indian neonatal units are different to African and other low-income country neonatal units. If the HELIX trial results suggest that cooling is safe and effective in Indian neonatal units, the next stage would be to evaluate cooling therapy in under resourced neonatal units in Africa, and other low-income countries.

5 Trial design

This is a two arm un-blinded pragmatic randomised controlled trial of whole body cooling versus standard care, after neonatal encephalopathy in low and middle-income countries. We plan to randomise 408 babies in this trial, for which we anticipate approximately 1200 babies will have to be screened for eligibility.

The treatment duration (cooling therapy) is 72 hours, however the temperature of all recruited babies will be monitored during the first week after birth. Any temperature rise over >37.5^{0}C will be active treated, both in the cooling and usual care arms, as fever increases the brain injury and adverse outcomes after neonatal encephalopathy. The neurological outcomes will be assessed between 18 to 22 months of age. The trial duration will be 4 years, consisting of a 4 week start up period, 24 month recruitment period, a 18 month follow-up period, and 5 months for data analysis and write up.

5.1 Trial objectives

Primary objective

To examine whether whole body cooling to 33.5^{0}C initiated within 6 hours of birth and continued for 72 hours reduces death or neurodisability at 18 months after neonatal encephalopathy in low and middle-income countries.

Secondary objective

- To examine if whole body cooling reduces neonatal mortality (30 days) and mortality at 18 to 22 months after neonatal encephalopathy.
- To examine if whole body cooling reduces moderate or severe neurodisability at 18 to 22 months in survivors after neonatal encephalopathy.

5.2 Trial outcome measures

Primary outcome measure

Death or moderate or severe neurodisability at 18 to 22 months, disability being defined as any of the following – Bayley scales of infant development (Version III) composite cognitive and motor score <2SD[19](<70); gross motor function classification system (GMFCS) level > I[20]; impaired vision despite correction; hearing impairment requiring amplification to understand commands.

Secondary outcome measures

Short term (before discharge from hospital):

- Mortality from any cause
- Major intracranial haemorrhage
- Gastric bleeds (fresh blood > 5 ml from nasogastric tube)
- Persistent hypotension (mean blood pressure < 40 mm of Hg requiring inotropic support)
- Pulmonary haemorrhage (Copious bloody secretions with clinical deterioration requiring change(s) in ventilatory management)
- Persistent pulmonary hypertension (Severe hypoxemia disproportionate to the severity of lung disease with a significant pre-and post ductal saturation difference on pulse oximetry)
- Prolonged blood coagulation time requiring blood products.
- Culture proven early onset sepsis (isolation of a pathogenic organism from blood or cerebrospinal fluid along with clinical evidence of sepsis and elevation of C-reactive protein)
- Necrotising enterocolitis (defined as abdominal distension, increased gastric aspirates and/or blood in stools together with abdominal X-ray showing bowel oedema, pneumatosis or pneumoperitoneum, i.e. Bell's staging 2 or 3)
- Cardiac arrhythmia
- Severe thrombocytopenia
- Persistent metabolic acidosis
- Renal failure
- Pneumonia
- Subcutaneous fat necrosis
- Neurological examination at discharge.
- Duration of hospitalisation

Long term (18 to 22 months):

- Mortality
- Severe neurodevelopmental disability (any of: (i) Bayley III composite cognitive and motor score <3SD[19] (ii) GMFCS levels III,IV,V, blindness; or profound hearing loss (inability to understand commands despite amplification)
- Microcephaly (head circumference more than 2 standard deviations below the mean)

6 Selection and withdrawal of subjects

6.1 Inclusion criteria

All three criteria below should be met

1. Age ≤ 6 hours, Birth-weight ≥1.8kg, Gestation ≥36 weeks based on available information regarding last menstrual period or ultrasound)

2. Need for continued resuscitation at 5 minutes of age and/or 5 minute Apgar score <6 (for babies born at hospital) or lack of cry by 5 minutes of age (for babies born at home)
3. Evidence of moderate or severe encephalopathy at < 6 hours of age on a structured clinical examination (Table 3).

6.2 Exclusion criteria

- Absent heart rate at 10 minute of age despite adequate resuscitation.
- Major life threatening congenital malformation.
- Migrant family or parents unable/unlikely to come back for follow up at 18 month.
- Lack of parental consent.

6.3 Screening evaluation and neurological examination

All infants admitted to the neonatal unit with perinatal asphyxia will be screened for eligibility. Out-born babies meeting the inclusion criteria will be eligible for recruitment, irrespective of the temperature at admission to the neonatal unit. Potentially eligible cases will have a detailed neurological examination by the principal investigator at the site or the designated research fellow who is trained and accredited in NICHD neurological examination. Briefly, this scoring system consists of 6 categories. The highest count in each level is scored.

The level of encephalopathy will be assigned based on which level of signs (moderate or severe) predominates among the 6 categories. If moderate and severe signs are equally distributed, the designation is then based on the highest level in Category 1: The level of consciousness. If consciousness level is also equal, then encephalopathy score is allocated based on the highest score for tone.

Infants who have seizures will be moderate or severe neonatal encephalopathy depending on the neurologic exam. Seizures with normal or mild encephalopathy or moderate encephalopathy on neurologic exam will be “Moderate encephalopathy”. Seizures with severe encephalopathy will be “Severe encephalopathy”

Table 3. Clinical examination for encephalopathy

CATEGORIES (TOTAL 6)	SIGNS OF NEONATAL ENCEPHALOPATHY (NE) IN EACH CATEGORY **(Circle the most appropriate level)**			
	NORMAL	MILD NE	MODERATE NE	SEVERE NE
**1. Level of consciousness**				
	Alert, Responsive to external stimuli (state dependent, eg. post feeds)	Hyper-alert, has a stare, jitteriness, high-pitched cry, exaggerated responds to minimal stimuli, inconsolable	Lethargic	Stupor/coma
**2. Spontaneous activity**				
	Changes position when awake	Normal or Decreased	Decreased activity	No activity
**3. Posture**				
	Predominantly flexed when quiet	Mild flexion of distal joints (fingers, wrist usually)	Moderate flexion of distal joint, Complete extension	Decerebrate
**4. Tone**				
	Strong flexor tone in all extremities + strong flexor hip tone	Normal or Slightly increased peripheral tone	Hypotonia (focal or general) or Hypertonia	Flaccid<br>Rigid
**5. Primitive reflexes** (Circle only the highest level in each sign; The maximum score is only one in any one category)				
Suck	Strong, easily illicit	Weak, poor	Weak but has a bite	Absent
Moro	Complete	Partial response, Low threshold to illicit	Incomplete	Absent
**6. Autonomic system** (Circle only the highest level in each sign; The maximum score is only one in any one category)				
Pupils	In dark: 2.5-4.5 mm.<br>In light: 1.5-2.5 mm.	Mydriasis	Constricted	Deviation/ dilated/ non-reactive to light
Heart rate	100-160 bpm	Tachycardia (HR > 160)	Bradycardia (HR < 100)	Variable HR
Respiration	Regular respirations	Hyperventilation (RR > 60/min)	Periodic breathing	Apnea or requires ventilator
**TOTAL SCORE**				
* Seizure	None	None	Yes / No	Yes / No

Infant who has seizure will be Moderate or Severe NE depending on the neurologic exam. Seizure with normal or mild NE or moderate NE on neurologic exam will be "Moderate NE". Seizure with severe NE will be "Severe NE".

The level of encephalopathy will be assigned based on which level of signs (moderate or severe) predominates among the 6 categories. If moderate and severe signs are equally distributed, the designation is then based on the highest level in Category #1: The level of consciousness.

If the level of consciousness is equal, then allocate the NE stage based on the tone.

6.4 Treatment assignment and randomisation

As soon as parental consent has been obtained for an eligible infant, the recruiting clinician will obtain treatment assignment, which will be either to "usual care with cooling" or "usual care only", using a web based database with a central telephone randomisation back up (Sealed envelope; https://www.sealedenvelope.com). Minimisation will be used to ensure balance between the groups with respect to the severity of encephalopathy and centre.

6.5 Method of blinding

The intervention (cooling therapy) will not be blinded. However, the neurological outcome evaluation at 18 months will be undertaken by assessors masked to the treatment allocation.

6.6 Cooling therapy

Therapeutic hypothermia will be administered using any approved servo controlled cooling device (for example Tecotherm) that is already in clinical use in the UK and in India, at the discretion of the local investigators. Briefly this would consist of attaching the mattress to the

servo controlled device, refilling coolant, keeping the baby on the mattress, placing a rectal probe, switching the machine on and selecting the appropriate program. The cooling device will maintain the rectal temperature of the baby within 33^0C to 34^0C degrees, and will alarm when temperatures are out of range (for example – displacement of the rectal probe). The clinical team will record hourly rectal temperature in the clinical data collection form. After 72 hours of cooling, the baby will be automatically re-warmed at 0.5^0C per hour by the cooling machine.

6.7 Criteria for stopping cooling therapy

- Refractory hypotension (mean blood pressure < 25 mm hg) despite optimal inotropic and volume support.
- Life threatening/massive haemorrhage.
- Parental or clinician request to stop cooling therapy.

7 Supportive care and monitoring

General management of babies will be standardised at the participating centres, and it will not be permissible to therapies like steroids, magnesium or mannitol, or other experimental therapies in recruited infants.

Hourly vital signs (respiratory rate, saturations, heart rate, non invasive blood pressure, rectal temperature) will be recorded in the HELIX Case report form, in all infants. Additional monitoring will be dictated by the clinical condition and by the local guidelines.

Infants may also receive intravenous fluids, antibiotics, ventilatory support, inotropes, blood products, sedation, muscle relaxants, and anti-convulsants as per the local clinical protocol. Ventilation is not mandatory for providing cooling therapy, and in fact most infants in the HELIX feasibility study, and in other cooling trials from low and middle-income countries were not ventilated. Ventilated infants and infants with excessive shivering may also receive sedation (fentanyl, morphine or chloral hydrate) as per the local clinical protocol.

Detailed neurological examination (using a standard proforma in the case report form) will be performed within six hours of birth, and at discharge from hospital.

7.1 Baseline assessments and data collection

The following data points are to be recorded in the case report form.

1. Maternal (antenatal) and delivery details including resuscitation details
2. Time and date of birth, time of randomisation and start of cooling
3. Birth weight, gestation and gender
4. Hourly rectal temperature profile in all infants for the first 90 hours.
5. NICHD neurological examination within 6 hours and at the time of discharge
6. Full blood count (including platelets, CRP and differential white cell count) within six hours after birth, and between day 4 and day 7.
7. Blood culture (0.5 ml) within 6 hours of birth, and between day 4 and 7.
8. Biochemical series (including blood gas, sugar, urea, creatinine, electrolytes, and coagulation profile)..
9. Cranial US examination (within 72 hours) to examine for major intracranial bleeds.

Each case report form will be scanned and emailed to the HELIX trial manager at Imperial College London for quality checks within 48 hours of completion. The signed off case report forms will be entered into the trial database.

7.2 Exploratory sub studies

Detailed screening for perinatal infection
It is likely that perinatal infection may have a significant effect on the safety and therapeutic efficacy of cooling therapy. Although facilities for such screening are not available at the recruiting public sector hospitals, the samples for these will be collected and analysed in collaboration with a private sector hospital in Bangalore, under the supervision of infection disease experts at Imperial College London.

This will include:

- Blood (1 ml) for targeted polymerase chain reaction (PCR) for common bacterial pathogens and gene expression studies, collected within six hours and between 4 to 7 days after birth.
- A small section of the umbilical cord (fetal end) for histopathological examination for funisitis. A section from the placenta will be also collected whenever feasible.

The tissues will be stored (-20^{0}C) at the recruiting centres in the first instance, and then analysed in batches in an Indian laboratory, at a later stage. The results of this will be fed back to the local principal investigator, who will inform parents about the same.

Magnetic resonance imaging and spectroscopy
The treatment effects of cooling therapy on brain injury can be accurately assessed and quantified using magnetic resonance imaging (MRI) and spectroscopy (MRS) in neonatal encephalopathy. Although facilities for these may not be available at the recruiting public sector hospitals, a public – private sector partnership in India has been set up to perform MR imaging. All recruited infants (surviving beyond 1 week) will have an MR scan at an adjacent private 3 Tesla MRI centre between 7 to 14 days of age.

MR imaging is routinely performed in all babies with neonatal encephalopathy in the UK and other high-income countries, to understand the extent of brain injury and to prognosticate long term outcomes. Increasingly MR scans are performed in larger neonatal centres in India as well. The highest prognostic accuracy is obtained when the imaging is performed between 7 to 14 days in these babies, and the MR images are more difficult to interpret after first month. There is extensive safety data, on use of sedation, in particular chloral hydrate, for neonatal MR imaging. Unlike CT scan or X rays, MR imaging do not involve any radiation. Strict standard operating procedures will be followed in performing the MR scan, which includes screening and removal of all ferromagnetic objects on the baby (for example metallic buttons on clothes, ECG electrodes, standard pulse oximeters), and continuously monitoring of saturation and heart rate using an MR compatible pulse oximeter by an doctor or nurse trained in neonatal resuscitation).

The MR images will be reported by a local radiologist (at the MRI centre) in all babies, and will be fed back to the parents in real time by the clinicians in charge of the baby.

The MR sequences will be optimised and harmonised prior to recruitment of babies by Imperial College London MR physicists using the Magnetic Resonance Biomarkers in Neonatal Encephalopathy (MARBLE) study protocol. (http://public.ukcrn.org.uk/search/StudyDetail.aspx?StudyID=11520).

The anonymised MR images and spectroscopy data will be encrypted and transferred to the Centre for Perinatal Neuroscience by Imperial College London file transfer protocols for analysis and storage.

7.3. Follow up and neurological assessments

A dedicated HELIX research nurse at each recruiting centre will contact the parents every 4 to 6 months by telephone and will record the following information.

1. General health status of the baby
2. Any change in home address or telephone number

Neurodevelopmental outcome scores will be assessed at 18 months of age (all babies) using the Bayley Scales of Infant Development (BSID-III) and Gross Motor Function Classification System,[21,22] masked to the treatment allocation, by a trained and certified examiner at the recruiting centre. All neurodevelopmental outcome assessments will be video recorded and stored for governance purposes. Anthropometric data (growth and head circumference) will be also collected.

The examiner will feed back the results of the neurodevelopmental outcome tests to the parents, immediately after the assessment. A copy of this report will be provided to the site principal investigator (Head of Neonatology at the recruiting center), for any clinical management.

Severe disability will be defined as any one of the following: Bayley III cognitive composite score <70, Gross Motor Function Classification System level 3-5, hearing impairment requiring hearing aids/cochlear implant, or blindness.

Moderate disability will be defined as cognitive composite score 70-84 and one or more of the following: Gross Motor Function Classification System level II, hearing impairment with no amplification/cochlear implant, or a persistent seizure disorder.

8 Adverse events

All known adverse events relating to neonatal encephalopathy and cooling therapy are described in the parent information leaflet and will be part of obtaining the informed research consent, prior to the start of cooling therapy.

The following clinical events occur due to the underlying disease (neonatal encephalopathy). Cooling trials from high-income countries have shown that cooling therapy reduces/does not increase the incidence of many of these clinical events in encephalopathic babies. The only safety effect that is noted to be increased is thrombocytopenia..

1. Death during neonatal period or during infancy
2. Brain injury on magnetic resonance imaging
3. Adverse neurodevelopmental outcome at 18 months and at child hood
4. Persistent pulmonary hypertension
5. Metabolic imbalances
6. Cardiac arrhythmia
7. Renal failure
8. Coagulopathy
9. Gastric bleeds

Cooling therapy may increase the risk of the following adverse events noted in the previous randomised controlled trials.

1. Thrombocytopenia and increased need for platelet transfusions
2. Subcutaneous fat necrosis

All adverse events are expected to occur within the cooling period (first 72 hours) or within 72 hours of re-warming. Adverse reactions occurring subsequently (after 1 week of life), except sub cutaneous fat necrosis, will not be considered as intervention related. Sub cutaneous fat

necrosis may occur several weeks after the therapy.

If an UNEXPECTED serious adverse event occurs (i.e. an event not mentioned in the above list), it should be reported to the HELIX trial manager at the Centre for Perinatal Neuroscience (CPN) at Imperial College London within 24 hours, using one of the Serious Adverse Event Report forms. The HELIX trial manager will ensure that the HELIX Independent Data Monitoring Committee and the Research Ethics Committee are informed accordingly

Serious adverse events that may be due to hypothermia are:

- Cardiac arrhythmia.
- Life threatening bleeds.
- Major venous thrombosis not related to an infusion line.

9. Statistical methods

Prior to the first interim analysis of un-blinded data, a detailed statistical analysis plan will be developed for approval by the Independent Data Monitoring Committee and the Trial Steering Committees (see section 10.1 regarding handling of the interim analysis results). The primary analysis will be a comparison of the infants assigned to usual care plus whole body cooling with those infants assigned to usual care at randomisation (i.e. intention-to-treat analysis population), regardless of deviation from the protocol or whether they received the allocated intervention. Demographic factors, clinical characteristics and outcomes will be summarised with counts (percentages) for categorical variables, means (standard deviation [SD]) for normally distributed continuous variables, or medians (inter-quartile [IQR] or entire range) for non-normally continuous variables.

In order to establish both the magnitude and direction of the effects of whole body cooling intervention, comparative statistical analysis will entail calculating the risk ratio (RR) plus 95% confidence interval (CI) for the primary outcome. The chi-square test will be used to determine statistical significance, with a 5% significance level used.

Secondary outcomes will be evaluated using a 1% level of statistical significance, with 99% CIs reported, in order to take account of the number of outcomes analysed. The Chi-square test or Fisher's exact test will be used to analyse categorical outcomes with risk ratios reported with 99% CIs. The unpaired t-test will be used to analyse normally distributed continuous outcomes, with the mean difference (plus 99% CI) reported. Non-normally distributed continuous outcomes will be transformed to normality, or alternatively analysed using the Mann-Whitney test. If the latter approach is used, the median difference (plus 99% CI) between groups will be reported.

Logistic regression will be used to perform an adjusted analysis for the primary outcome to investigate the impact of stratification/known prognostic factors including the stage of neonatal encephalopathy.

Analysis of secondary outcomes will be clearly delineated from the primary analysis in any statistical reports produced. Results will be reported according to the CONSORT statement.

The sample size is based on being able to detect a clinically significant 30% relative risk reduction in death or moderate/severe disability from 50% in the usual care arm to 35% in the intervention (cooled) arm. Using a two-sided 5% significance level and an 80% power, 183 babies per arm are required. Assuming a loss to follow-up rate of around 10%, this comparison requires 204 babies per group, 408 babies in total, to be recruited. If in case, the adverse outcomes (death and moderate/severe disability) are higher (~65%) in the usual care arm, then this sample size would provide 94% power to detect a 30% relative risk reduction with cooling.

10. Trial organisation

a. Independent Data Monitoring Committee (IDMC)

An independent Data Monitoring Committee (IDMC) will review the study's progress. The IDMC will be independent of the trial organisers. Interim analyses will be supplied, in strict confidence, to the IDMC, as frequently as its Chair requests by the HELIX trial statistician. Meetings of the committee will be arranged periodically, as considered appropriate by the Chair.

The IDMC will inform the Trial Steering Committee (TSC) if in their view

- There is proof beyond reasonable doubt that the data indicate that any part of the protocol under investigation is either clearly indicated or contra-indicated, either for all infants or for a particular subgroup of trial participants
- It is evident that no clear outcome will be obtained.
- Safety signal

Appropriate criteria for proof beyond reasonable doubt cannot be specified precisely. A difference of at least 3 standard deviations in the interim analysis of a major endpoint may be needed to justify halting, or modifying, such a study prematurely. Unless modification or cessation of the study is recommended by the IDMC, the TSC, investigators, collaborators and administrative staff (except those who supply the confidential information) will remain ignorant of the results of the interim analysis. Collaborators and all others associated with the study, may write to the IDMC via the HELIX Co-ordinating Centre, to draw attention to any concern they may have about the possibility of harm arising from the treatment under study, or any other relevant matters.

The membership of IDMC is given below

- Professor Abbot Laptook (Chair, Professor for Neonatology, Brown University, USA)
- Professor Shabbar Jaffar (Faculty of Epidemiology and Population Health, London School of Hygiene and Tropical Medicine, London, UK)
- Professor Niranjan Thomas (Professor of Neonatology, Christian Medical College, Vellore, India)

The HELIX trial statistician will provide the trial data for IDMC meetings and interim analysis as requested by the IDMC chair.

b. Trial Steering Committee

The Trial Steering Committee (TSC) will provide overall supervision of the study of the Sponsor. Its terms of reference are:

1. To monitor and supervise the progress of the HELIX trial towards its interim and overall objectives.
2. To review at regular intervals relevant information from other sources (e.g. related studies).
3. To consider the recommendations of the Independent Data Monitoring Committee.

The TSC will meet (in person or by teleconference) at least once a year. The membership is given below

- Chair – Professor Sidharth Ramji (Medical Superintendent, Maulana Azad Medical College, New Delhi, India)
- Dr Jethro Herberg – Consultant in Paediatric Infectious Diseases, Imperial College London
- Dr Dominic Wilkinson – Consultant Neonatology and Director of Bioethics, John Radicliffe Infirmary, Oxford

- Dr Paul Basset (HELIX Trial statistician, Stats Consultancy, London)
- Professor Seetha Shankaran (Director of Neonatal Perinatal Medicine, Wayne State University, Michigan)
- Dr Sudhin Thayyil (Chief Investigator)

c. Project Management Group

The Project Management Group (PMG) will oversee all aspects of the day-to-day running of the study, and will consist of the investigators and the HELIX trial staff, based at the HELIX Co-ordinating Centre in India, and the Centre for Perinatal Neuroscience, Imperial College London. PMG will hold a monthly teleconference of all HELIX investigators for the entire duration of the trial to discuss the data quality and recruitment.

The responsibilities of the PMG include:

- Appointment and training of the local research staff for the HELIX trial
- Case recruitment at participating centres
- Distribution and supply of data collection forms and other appropriate documentation for the trial
- Data collection and management
- Organisation of the follow-up
- Data entry and cleaning
- Data analysis
- Collection of adverse event data

11. Ethical and regulatory considerations

a. Consent

A parent information leaflet will be given to parents to consider participating in the study.
The attending physician will meet with parents during the intervention period to ensure that they understand the study procedures and continue to consent to participate in the study.
Written informed consent will be obtained from parents after a full verbal and written explanation of the study. The consenting processes will be video recorded as per the current Indian clinical trial regulations. The digital video recordings will be securely stored in a central server.

Approval for the study will be obtained from Imperial College London and the Local Research Ethics Committee of each participating hospital, before the first case is recruited. Each participant's right to refuse or withdraw from the study without giving reasons will be respected at all times. A withdrawal form will be filled in and authorisation for use of the previously collected data will be obtained.

The site principal investigator will retain the original of each patients signed informed consent form. Should a patient require a verbal translation of the trial documentation by a locally approved interpreter/translator, it is the responsibility of the individual investigator to use locally approved translators.

The parent information leaflet and consent form will be translated into the local languages (Kannada, Hindi, Tamil, Telengu). All sections of the approved documents must appear in the translation.

b. Ethical committee review

All original trial documentation and any subsequent amendments will be approved by the Sponsor and by the relevant ethical bodies, prior to their implementation. All correspondence with the REC will be retained in the Trial Master File/Investigator Site File. Annual reports will be

submitted to the REC in accordance with Imperial College London requirements. It is the Chief Investigator's responsibility to produce the annual reports as required.

Each participating site in India will have local research ethics and other regulatory approvals as per the local regulations.

c. Declaration of Helsinki and Good Clinical Practice

The trial will be performed in accordance with the declaration of Helsinki, the conditions and principles of Good Clinical Practice, the protocol and applicable local regulatory requirements and laws.

d. Data protection and patient confidentiality

All investigators and trial site staff involved in this trial must comply with the requirements of the Data Protection Act 1998 and Trust Policy with regards to the collection, storage, processing and disclosure of personal information and will uphold the Act's core principles. The site investigators at each site will ensure that only linked anonymised data is received by the HELIX trial team.

Hard copies of case report forms, and consent forms will be stored inside a locked cupboard in a designated HELIX research office at each recruiting centre, under the supervision of the site principal investigator. Only the HELIX research nurse, research doctor, data entry clerk and principal investigator at each site will have access to the hard copies. Linked anonymised electronic data will be stored in a GCP complaint secure UK based server, with daily back up. The HELIX Trial statistician, manager, chief investigator, co-chief investigator and Independent data safety monitoring committee will have access to the electronic trial data. Principal investigators from each site will have access to the data from their site.

e. GCP Training

All trial staff must hold evidence of appropriate GCP training or undergo GCP training prior to undertaking any responsibilities on this trial. This training should be updated every 2 years or in accordance with the Sponsor's policy.

12. Sponsorship, Financial and Insurance

The trial is sponsored by Imperial College London, and funded by the Gates foundation, Imperial Biomedical Research Centre, and Imperial College London. Imperial College London will arrange insurance for negligent harm caused as a result of protocol design and for non-negligent harm arising through participation in the clinical trial.

13. Monitoring, Audit & Inspection

The site principal investigator must make all trial documentation and related records available for the monitoring by the study team and by the Sponsor. All patient data will be handled and treated confidentially.

The study team's monitoring frequency will be determined by an initial risk assessment performed prior to the start of the trial. A detailed monitoring plan will be generated detailing the frequency and scope of the monitoring for the trial. Throughout the course of the trial, the risk assessment will be reviewed and the monitoring frequency adjusted as necessary.

14. Protocol Compliance and Breaches of GCP

Prospective, planned deviations or waivers to the protocol are not allowed under the UK regulations on Clinical Trials and must not be used. For example, it is not acceptable to enrol a subject if they do not meet one or more eligibility criteria (example babies with mild encephalopathy or no encephalopathy) or restrictions specified in the trial protocol.

Protocol deviations, non-compliances, or breaches are departures from the approved protocol. They can happen at any time, but are not planned. They must be adequately documented on the relevant forms and reported to the Chief Investigator and Sponsor immediately. Deviations from the protocol which are found to occur constantly again and again will not be accepted and will require immediate action and could potentially be classified as a serious breach. Any potential/suspected serious breaches of GCP must be reported immediately to the Sponsor without any delay.

15. Publications policy

Ownership of the data arising from this trial resides with the trial team. On completion of the trial the data will be analysed and tabulated and a Final Study Report prepared. Consort Guidelines and checklist are reviewed prior to generating any publications for the trial to ensure they meet the standards required for submission to high quality peer reviewed journals etc. http://www.consort-statement.org/

A copy of the study results will be also given to the parents of all recruited babies, if they wish to. This will be recorded at the time of recruitment, and again during follow up.

16. Appendices

Appendix 1 - Authorisation of participating sites and responsibility of the site PI

1. Documentation to be provided to the sponsor before recruitment begins

- Copy of the local research ethics and other local regulatory approvals.
- Back translated patient information leaflets and consent forms.
- International Conference on Harmonisation Good Clinical Practice (ICH-GCP) online certification for the local trial team.

2. Procedure for opening a new recruiting site

- Site visit and risk assessment by the Imperial College London team.
- A dedicated HELIX research nurse appointed and trained in HELIX protocol.
- ICH-GCP training of the local research teams by the Imperial College team.
- Dedicated desktop computer and printer/scanner for scanning and e-mailing of case report forms to Imperial College London.
- Internet and secure phone connections identified for randomisation.
- Staff training on cooling and data collection.
- Video recorder for recording the consent.
- Adequate safe storage of the consent forms and case report forms.
- Harmonisation and optimisation of 3T MRI scanner at the private MR facilities adjacent to the recruiting centres.
- Standard operating procedures (SOP) and delegation logs.

3. Legal responsibilities of the site principal investigator

The detailed responsibilities are listed in the Participating Site Agreement. Key responsibilities are given below.

- Attendance at the trial initiation meetings/teleconferences.
- Ensure ICH-GCP certification of local researchers.
- Training of new members of the local trial team in the protocol and its procedures
- Ensure that the investigator folder and consent forms are accurately maintained and stored.
- Dissemination of important safety or trial related information to all stakeholders within their site.
- Safety reporting within the timelines.
- Ensuring the accuracy of data entry into the case report forms
- Facilitate the trial monitoring by the HELIX trial manager.

Imperial College London
Centre for Perinatal Neuroscience

Hypothermia for Encephalopathy in Low and Middle Income Countries (HELIX) Trial

STUDY ENROLLMENT PATHWAY

Baby admitted to the Neonatal Unit with suspected HIE

Yes

Is the equipment available for both cooling and non-cooling groups?

No → Patient cannot enroll the study if equipment is being used by other baby (both cooling machine and non-cooling rectal probe must be available)

Yes

Does the baby meet ALL of the inclusion criteria and NONE of the exclusion criteria (see Screening Tool on reverse page)

Yes

Call your Local Helix Trial Consultant to perform NICHD Neurological Examination and obtaining informed consent from Parents.

Yes

According to the NICHD criteria, does this baby have moderate or severe HIE?

Yes

Have the Parents consented for the enrollment of the baby in this trial?

Original CF+PIL filed in medical notes + copies for parents + copies for research folder, + scan saved in local computer

Yes

Once the parents have consented for the baby to take part in the study, you can now randomise the patient (see Randomisation Help Page) and start completing the Case Report Form (CRF).

For any queries, please email helix.study@imperial.ac.uk or call +4420 3313 2473 or +919840653244

Helix_Enrollment&Screening_V2.0_12/08/2015

	STUDY PERIOD												
	**Enrolment**	**Allocation**	**Post-allocation**										**Close-out**
**TIMEPOINT****	**$-t_1$**	**0**	**Day 1**	**Day 2**	**Day 3**	**Day 4**	**Day 5**	**Day 7-14**	**Hospital discharge**	**3 m**	**6 m**	**12 m**	**18m to 22 m**
**ENROLMENT**													
**Antenatal history**	X												
**Birth history**	X												
**Postnatal history**	X												
**Neurological examination**	X												
**Eligibility screen**	X												
**Video recording of the consenting process**	X												
**Written informed consent**	X												
**Randomisation**		X											
**INTERVENTION (Age < 6 hours at start of intervention)**													
**Whole body cooling**			⟷	⟷	⟷								
**Usual care**			X	X	X								
**ASSESSMENTS**													
**Rectal temperature (1h)**		X	X	X	X	X	X						
**Blood gas analysis**	X		X	X	X								
**Full blood count**	X					X							
**Renal and liver function**	X												
**Coagulation screen**	X												
**Infection screen**	X					X							
**qPCR**	X												
**Transcriptomics**	X												
**Cranial ultrasound**			X										
**Neurological examination**			X	X	X	X			X				
**MRI and MR spectroscopy**								X					
**Telephone follow-up**										X	X	X	
**Neurodevelopmental assessment**													X

Protocol for MRI scanning of babies on the HELIX Trial

1. All MR scans need to be performed between 4 to 14 days after birth. In exceptional cases where the baby is not clinically stable by this time, the MR scan may be delayed up to 3 to 4 weeks. Please discuss this with the HELIX Chief Investigator on a case by case basis.
2. Local research team should make necessary arrangement to book MRI slot and provide appropriate transport if travelling to another hospital. Patients should be escorted by one doctor and one nurse to and from the hospital where MRI will be performed.
3. The Nurse and doctor accompanying the patient should carry a resuscitation transport bag should the baby need
4. Parents should be informed that we will be expecting to find abnormalities on the brain MR in most babies, prior to the MR scan and explained that the team will discuss the results with them, once the scan has been reported.
5. All infants must have an MRI safety check done **before** the MRI scan. Please arrange this with the radiology team. A list of typical objects which can be unsafe for MRI is given below.
6. A neonatal doctor or an experienced neonatal nurse (trained in newborn life support) should assess the baby to make sure baby if fit for sedation. The baby must not have any respiratory distress (i.e. respiratory rate > 60 min or recession of the intercostal or subcostal areas), and saturations must be over 92% in air before giving sedation for the MR scan. The scan should be postponed if the baby has respiratory distress.
7. Remove any clothes with metal buttons/poppers or any shiny/silver thread and replace them by metal-free clothing. Ensure the baby has had a nappy change before the MR scan.
8. Ear protection must be applied to all infants undergoing MRI, in order to reduce noise exposure.
9. Apply the MR compatible monitor probes (oxygen saturation to fingers/toes of the baby and temperature probe to the chest/axilla of the baby). Use a thin linen or sheet to wrap around the cables and prevent contact with the infant's skin and aim to keep all cables straight.
10. Give a breast/NG/bottle feed approximately 60 minutes before the MR scan.
11. Give sedation: Oral / NGT / PR Chloral Hydrate, maximum dose 50mg/kg, only one dose should be given. This should be given at least 30 minutes before the MRI scan and ideally 30 min after the feed. If using PR Chloral, make sure the suppository is not expelled by bowel action.
12. Monitor the baby in a quiet dark place for 30 minutes before the MR scan, so that baby can settle asleep and avoid disturbing as much as possible.
13. Just before the MR scan place the baby on MedVac® mattress.
14. Gently transfer the baby into the MR room and place inside the head coil of the scanner.
15. A neonatal doctor or an experienced neonatal nurse (trained in newborn life support) should monitor the oxygen saturation, heart rate and temperature of the baby continuously during the MR scan and enter this into the monitoring form provided. This form should be filed in the patient's notes after the scan. Essential equipment for neonatal resuscitation including an ambu bag, different sized masks, intubation and suction equipment, emergency neonatal drugs, should be available in the MR suite. In the event of an emergency, local MR team should place a crash call according to local policy to ensure the fastest possible response. There should be facilities to summon immediate help from the hospital resuscitation team in case of a respiratory or cardiac arrest in the MR room,
16. All babies given sedation must be observed for a minimum of two hours after the MR scan. Prior to discharge home, the baby must be awake, have taken a good feed, and must have had an examination by the doctor or neonatal nurse.
17. The MR scan should be reported by the local radiologist, and the report fed back to the parents by the local site investigator/clinical team, and documented in the medical notes.

Ferromagnetic checks before MRI Scan.

All subjects MUST have a metal check performed **before** entering the MRI scanner. The table below is not an exhaustive list of all possible items but include common items you will need to check for safety. Please refer to local policy to check and confirm all items to be MR compatible.

Any object is assumed to NOT be compatible with the MRI scanner until proven otherwise. To be considered safe for scanning the exact specifications of any internal metallic object (PDA clip, intra-ventricular reservoir, etc) must be known and discussed with the radiographer before a child enters the scanner.

**Typical objects that need to be checked for MR safety**
Vascular Lines:<br>Arterial lines<br>Umbilical lines<br>Long lines
Fixation devices for lines, e.g. splints
PDA clips
Surgical implants/any history of surgical procedures
Scalp needles
Electronic name tags
Name tags with metal closures
Clothes with metal poppers
Ward pulse oximetry
Ward ECG leads
Ward temperature probes
Religious artefacts
Endotracheal tube holders
**Other important checks**
Baby had cares done (dry nappy)
Hearing protection applied

Applying Ear Protection before the MRI Scan

The MRI scan typically generates noise levels above 100dB. By applying the following protection, we expect to reduce the noise levels substantially. Ear protection must be applied to all infants undergoing MRI.

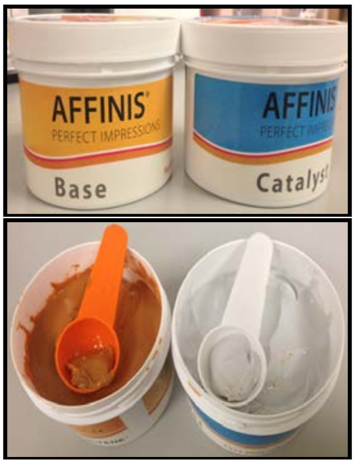

1. Mix equal parts of both components of the dental putty until you form an homogenised paste

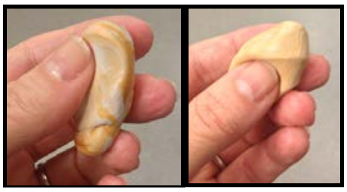

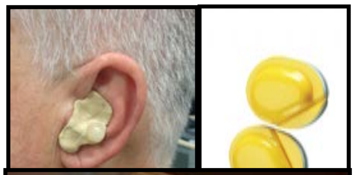

2. Apply the putty to cover the ear while soft (putty will start to harden).

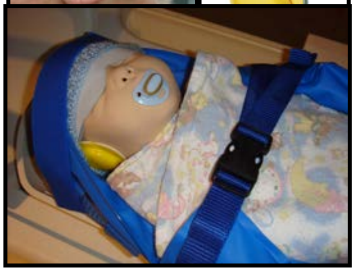

3. Apply ear muffs. Support with hat and/or velcro strap. Phototherapy eyeshades can fit both purposes of helping secure the earmuffs as well as helping baby to close the eyes and sleep. Baby is now ready to go into the MedVac®

4. After the scan, the earmuffs and putty can be easily removed without leaving any residue.

Applying MedVac® Infant immobiliser

MedVac® is an air-tight vacuum-chambered splint that provides effective immobilisation during the scan. The mattress is wrapped around the baby using velcro straps and the air inside the mattress is evacuated, so that it forms a firm mould around the baby.

- MedVac® can be set up outside the MR room, after a thorough metal check and appropriate positioning of the baby.
- All other checks, including ear protection etc. should be completed before placing the infant on the mattress.

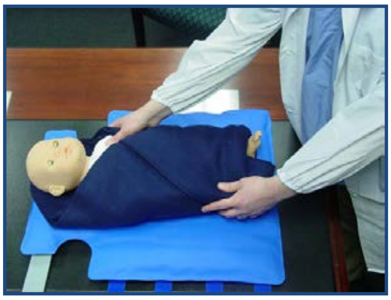

1. Wrap the baby in a thin sheet within the MedVac®. Leave any extremity used for saturation monitoring uncovered, enabling easy access to this.

2. Make sure the valve on the mattress is tightly closed. Place the infant on the mattress. Use the straps provided to loosely wrap it around the infant’s body (and head if needed).

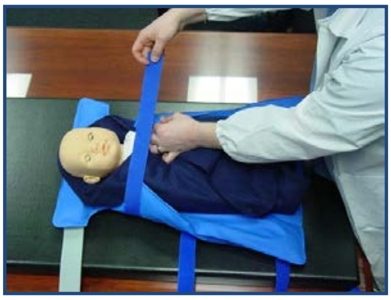

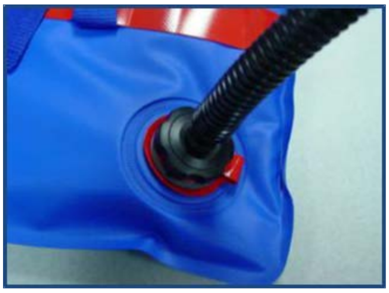

3. Evacuate the air within the mattress through the valve using either the wall mounted suction or hand pump provided.

4. Detach the hose and the baby is ready to be taken into the scanner room.

5. To re-inflate, simply open the valve.

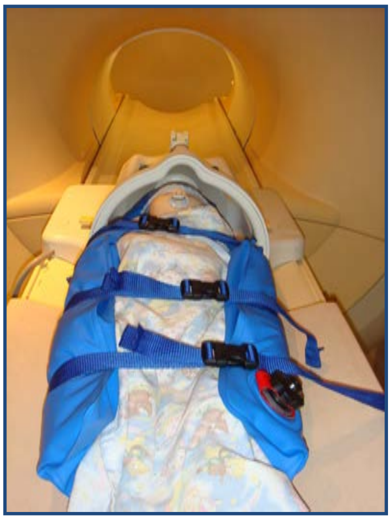

6. With the infant in the head coil, foam padding should be placed between the head and MedVac® for additional hearing protection.

For further instructions, please contact the Chief Investigator or visit the MedVac® manufacturer’s website at: http://cfimedical.com/medvac/

Monitoring Form (for all babies undergoing MRI as part of Helix Trial)

Patient Name	Date of Scan: ___/___/_____	Sedation Details:
DOB	Start Time:___:___	Drug used:______________________
Hospital Number	End Time:___/___	Dose:____________

**Location**	Please circle as appropriate: **D** Departing Neonatal Unit – **M** In MRI – **L** Leaving MRI – **A** Arrival in Neonatal Unit																			
	DMLA	DMLA	DMLA	DMLA	DMLA	DMLA	DMLA	DMLA	DMLA	DMLA	DMLA	DMLA	DMLA	DMLA	DMLA	DMLA	DMLA	DMLA	DMLA	DMLA
**Time**																				
**Axila Temp.**																				
**HR**																				
**$SatO_2$**																				
**RR/Vent.**																				
**Any change**																				
**Initials**																				

Notes:

Imperial College London

Centre for Perinatal Neuroscience

Hypothermia for Encephalopathy in Low and Middle Income Countries (HELIX) Trial

1. 3 plane survey
2. 3D T1 MPRAGE (Acquired Sag put reformatted in 3 planes)
3. Axial T2 TSE
4. 3 plane survey
5. PRESS MRS (2 sequence for visualising lactate)
6. STEAM MRS (13 sequences for NAA concentration)
7. 3 plane survey (exact same geometry as step 5)
8. DTI (32 directions, b750)

ALL OF THESE ARE TO BE USED WITHIN THE IEC NORMAL MODE OF OPERATION FOR SAR AND dB/dt.

3D T1 MPRAGE (Acquired sagittal and reformatted in 3 planes)

Matrix size = 256x256
In plane resolution = 0.82x0.82mm (FOV of 210x210mm)
Slice thickness = 1mm (50% overlap)
Slices = 240
TR/TE = 17/5ms
Inversion time = 1400ms
FA = 13o
NSA = 1
Parallel Imaging Reduction Factor = 1

Axial T2 TSE (Axial):

Matrix size = 288x288
In plane resolution = 0.5x0.5mm
Slice thickness = 3mm (10% slice spacing)
Slices = 35
TR/TE = 8800/130ms
Echo train length = 11
FA = 90°
NSA = 2
Parallel Imaging Reduction Factor = 1

Diffusion Tensor Imaging (Axial SE-EPI Diffusion)

Matrix size = 128x128
In plane resolution = 2x2mm
Slice thickness = 2mm (no slice gap)
Slices = 50
TR/TE = min/min
b value 1 = 0, 750 s/mm^2
NSA = 1
Directional resolution = High (32 directions)
Parallel Imaging Reduction Factor = 2

MRS (PRESS, 5mins & STEAM, 10mins)

15x15x15mm^3 voxel in the left thalamus.

For the SAGITTAL scans align the slices along the midline of the brain

For the AXIAL scans align the slices parallel to the line joining the splenium and genu of the corpus callosum

For the CORONAL scans align the slices with the brain stem

MR SPECTROSCOPY (MRS) PLANNING

Plan the voxel in the ***left thalamus*** using the axial T2 and sagittal T1 side by side. Avoid ventricular CSF as much as possible, and keep the voxel over the thalamus.

DO NOT CHANGE THE VOXEL SIZE OR MOVE IT BETWEEN SCANS, JUST PLACE IT FOR THE FIRST SCAN AND KEEP IT IN THE EXACT SAME PLACE FOR ALL OF STEPS 6 & 7

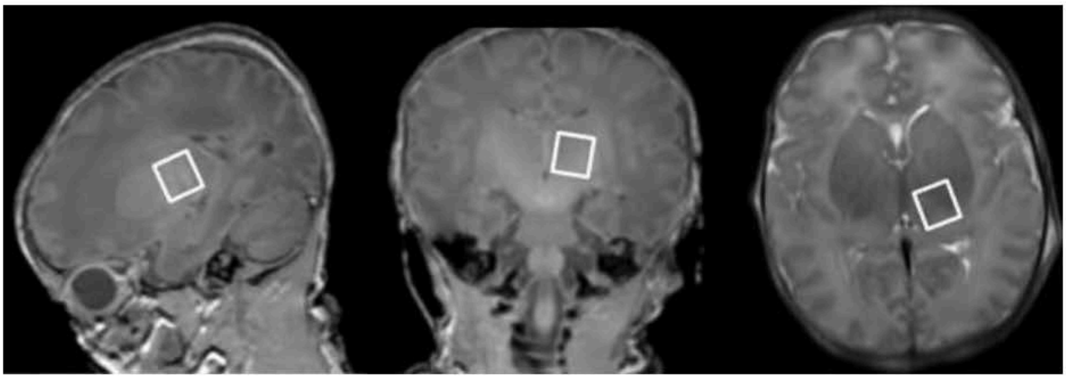

STEP 6 – PRESS MRS for visualising lactate (5min+shimming)

No.	Seq.	Water Suppress.	TR (ms)	TE (ms)	Phase cycling	Dynamics	Prep. Scans	Vector Size	PlanScan metabolite
**1**	PRESS	ON	2288	288	8	16	3	2048	NAA
**2**	PRESS	OFF	2288	288	8	1	1	2048	H_2O

STEP 7 – STEAM MRS for assessing NAA concentration (10 min)

No.	Seq.	Water Suppress.	TR (ms)	TM (ms)	TE (ms)	Phase cycling	Dynamics	Prep. Scans	Vector Size	PlanScan metabolite
**1**	STEAM	ON	1500	20	20	4	24	1	2048	NAA
**2**	STEAM	OFF	1500	20	20	4	1	1	2048	H_2O
**3**	STEAM	ON	5000	20	20	4	20	1	2048	NAA
**4**	STEAM	OFF	5000	20	20	4	1	1	2048	H_2O
**5**	STEAM	OFF	9030	20	20	1	1	1	2048	H_2O
**6**	STEAM	OFF	9040	20	40	1	1	1	2048	H_2O
**7**	STEAM	OFF	9060	20	80	1	1	1	2048	H_2O
**8**	STEAM	OFF	9090	20	140	1	1	1	2048	H_2O
**9**	STEAM	OFF	9130	20	220	1	1	1	2048	H_2O
**10**	STEAM	OFF	9170	20	300	1	1	1	2048	H_2O
**11**	STEAM	OFF	9270	20	500	1	1	1	2048	H_2O
**12**	STEAM	OFF	9420	20	800	1	1	1	2048	H_2O
**13**	STEAM	OFF	9770	20	1500	1	1	1	2048	H_2O

SAVING ANONYMISED MRS DATA

Inserting the hard drive into the scanner tower USB port, go to the Patient Administration window and select all of the datasets collected for that study. Now click on 'Disk Files'.

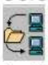

In the 'Export to Files' window, navigate to the hard drive by selecting the ... key, create a new folder, and rename this using the assigned patient code.

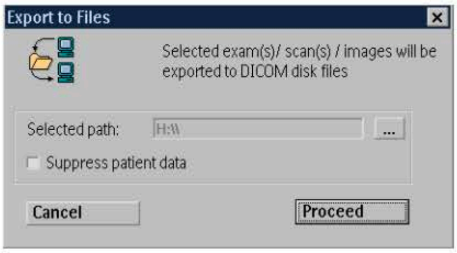

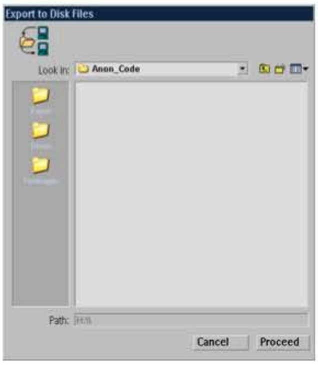

Click 'Proceed' to choose this folder as the destination for the series data. On returning back to the 'Export to Files' window, ***you must ensure that you tick the 'Suppress patient data' option.***

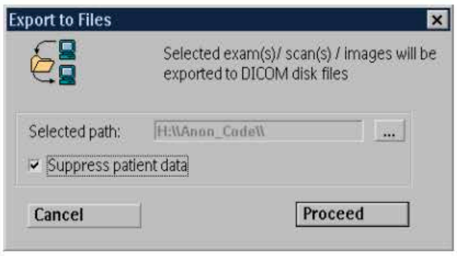

Only click proceed once you have checked that the destination folder is correct and that the patient data will be suppressed.

Once the data is on the external drive, please follow the instructions in the following section to send the data to the CPN Helix team at Imperial College London.

DATA TRANSFER – FOR ANONYMISED AND ENCRYPTED DATA ONLY

PART ONE

Once anonymised at the scanner, the subject data should only be identified by their study number. The following are some simple steps using a Windows computer to transfer the data to Imperial.

- Find the folder containing the anonymised imaging data. Right click on the folder and select 'Add to archive…' using WinRAR or 7zip.

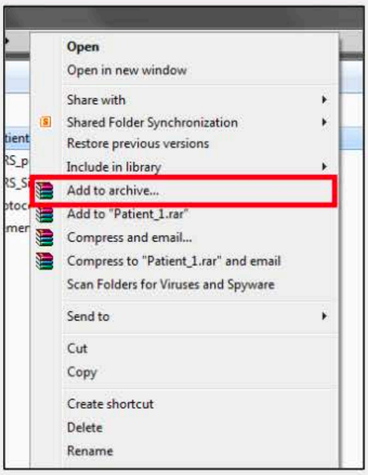

For computers with WinRAR

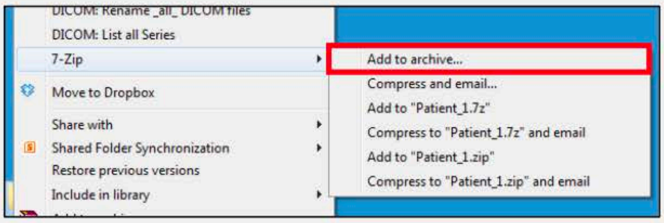

For computers with 7zip

- Type in a password that is easy for you to remember as shown below. **YOU WILL NEED TO SEND THIS PASSWORD TO ME LATER SO PLEASE DON'T FORGET IT!!** Once the password is set, click OK to close each window and create the file.

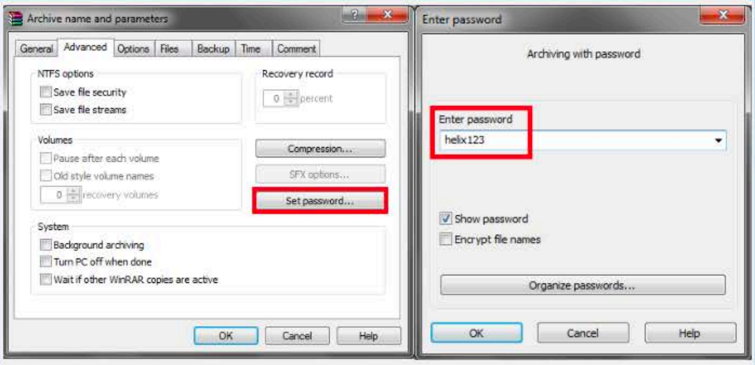

For computers with WinRAR

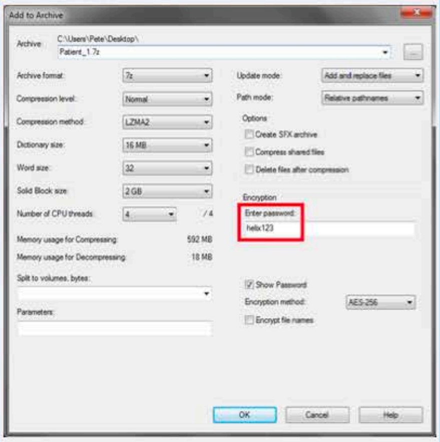

For computers with 7zip

Imperial College London

Centre for Perinatal Neuroscience

Hypothermia for Encephalopathy in Low and Middle Income Countries (HELIX) Trial

DATA TRANSFER – FOR ANONYMISED AND ENCRYPTED DATA ONLY

PART TWO

- Once the zip file is created, you need to send it to us at Imperial College London. Go to the Imperial College FileExchange site at https://icseclzt.cc.ic.ac.uk/. Select **Drop-off** and follow the instructions for people **without a request code**.

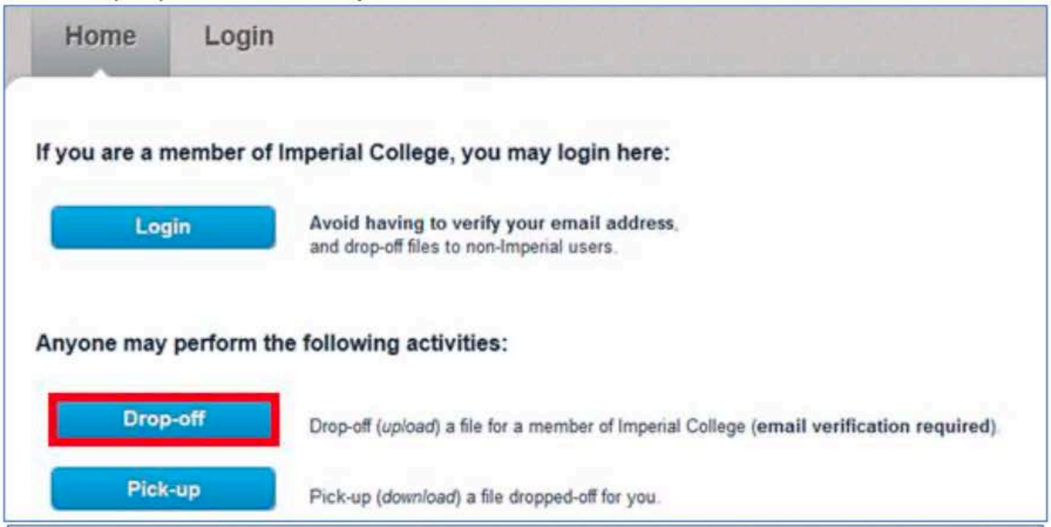

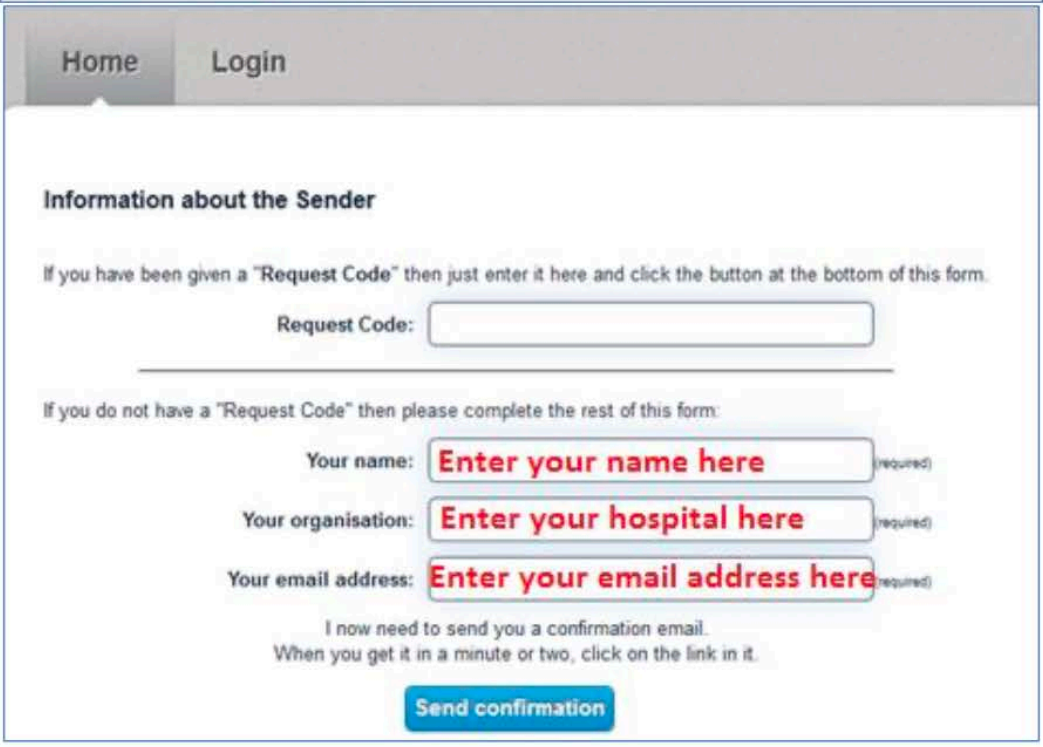

DATA TRANSFER – FOR ANONYMISED AND ENCRYPTED DATA ONLY

PART THREE

- In the **'To:'** box, click on the green icon to bring up the **'Add Recipients'** dialogue box as shown. Fill in my details *(Peter Lally, p.lally@imperial.ac.uk)*, select **'Add Recipient'** and then close the popup window. Browse for the .zip file you created earlier and select it, and then select **'Drop-off Files'**. I will be notified as soon as they are sent over. **Please email the encryption password to me separately or I won't be able to open the files!**

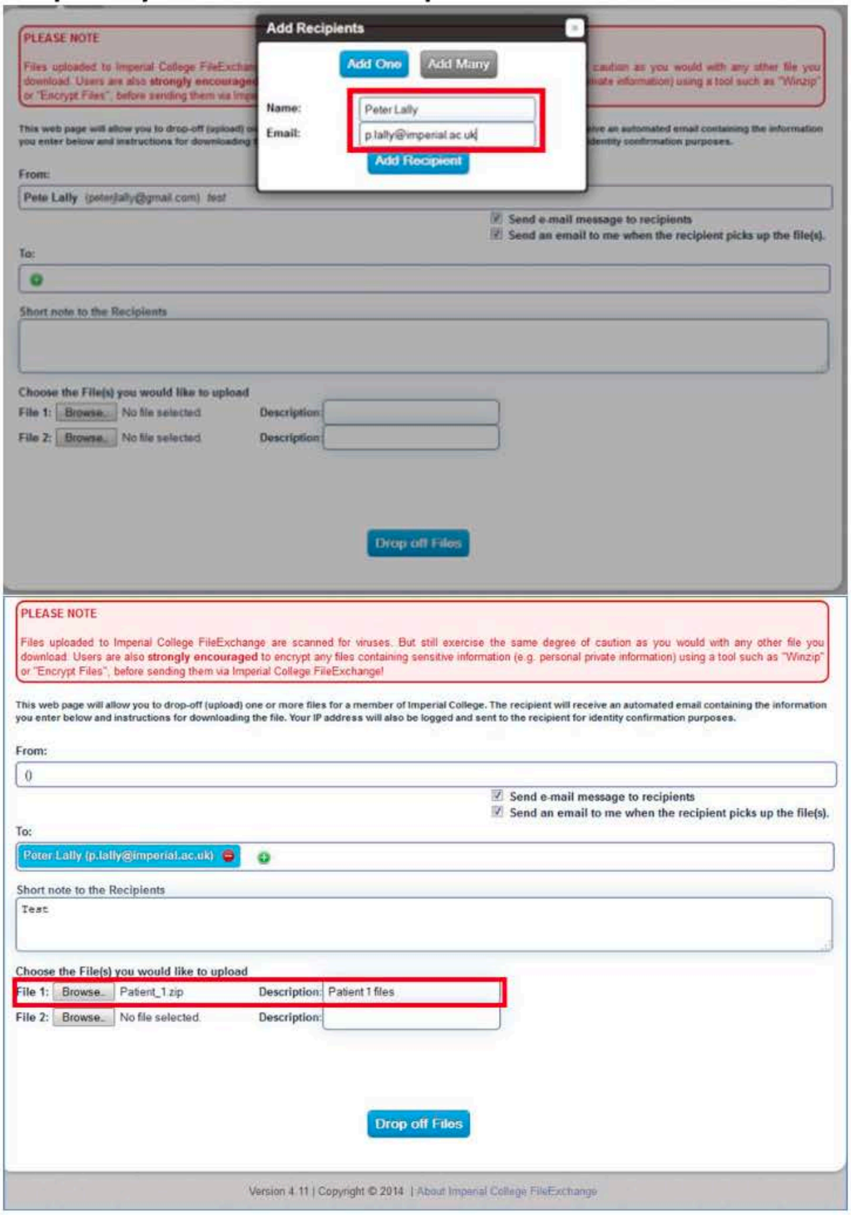

References

1. Lawn JE CS, Zupan J, for the Lancet Neonatal Survival Steering Team 4 million neonatal deaths: When? Where? Why? Lancet 2005;365:891-900.

2. Pierrat V, Haouari N, Liska A, Thomas D, Subtil D, Truffert P. Prevalence, causes, and outcome at 2 years of age of newborn encephalopathy: population based study. Arch Dis Child Fetal Neonatal Ed 2005;90:F257-61.

3. Robertson CM, Perlman M. Follow-up of the term infant after hypoxic-ischemic encephalopathy. Paediatr Child Health 2006;11:278-82.

4. Azzopardi DV, Strohm B, Edwards AD, et al. Moderate hypothermia to treat perinatal asphyxial encephalopathy. N Engl J Med 2009;361:1349-58.

5. Shankaran S, Laptook AR, Ehrenkranz RA, et al. Whole-body hypothermia for neonates with hypoxic-ischemic encephalopathy. N Engl J Med 2005;353:1574-84.

6. Gluckman PD, Wyatt JS, Azzopardi D, et al. Selective head cooling with mild systemic hypothermia after neonatal encephalopathy: multicentre randomised trial. Lancet 2005;365:663-70.

7. Jacobs SE, Berg M, Hunt R, Tarnow-Mordi WO, Inder TE, Davis PG. Cooling for newborns with hypoxic ischaemic encephalopathy. Cochrane Database Syst Rev 2013;1:CD003311.

8. Edwards AD, Brocklehurst P, Gunn AJ, et al. Neurological outcomes at 18 months of age after moderate hypothermia for perinatal hypoxic ischaemic encephalopathy: synthesis and meta-analysis of trial data. BMJ 2010;340:c363.

9. Shankaran S, Pappas A, McDonald SA, et al. Childhood outcomes after hypothermia for neonatal encephalopathy. N Engl J Med 2012;366:2085-92.

10. Shankaran S, Laptook AR, Pappas A, et al. Effect of depth and duration of cooling on deaths in the NICU among neonates with hypoxic ischemic encephalopathy: a randomized clinical trial. JAMA 2014;312:2629-39.

11. Shankaran S. Current status of hypothermia for hypoxemic ischemia of the newborn. Indian J Pediatr 2014;81:578-84.

12. Montaldo P, Pauliah SS, Lally PJ, Olson L, Thayyil S. Cooling in a low-resource environment: Lost in translation. Semin Fetal Neonatal Med 2014.

13. Maitland K, Kiguli S, Opoka RO, et al. Mortality after fluid bolus in African children with severe infection. N Engl J Med 2011;364:2483-95.

14. Higgins RD, Raju T, Edwards AD, et al. Hypothermia and other treatment options for neonatal encephalopathy: an executive summary of the Eunice Kennedy Shriver NICHD workshop. J Pediatr 2011;159:851-8 e1.

15. Zhou WH, Cheng GQ, Shao XM, et al. Selective head cooling with mild systemic hypothermia after neonatal hypoxic-ischemic encephalopathy: a multicenter randomized controlled trial in china. J Pediatr 2010;157:367-72.

16. Thayyil S, Shankaran S, Wade A, et al. Whole-body cooling in neonatal encephalopathy using phase changing material. Arch Dis Child Fetal Neonatal Ed 2013;98:F280-1.

17. Robertson NJ, Nakakeeto M, Hagmann C, et al. Therapeutic hypothermia for birth asphyxia in low-resource settings: a pilot randomised controlled trial. Lancet 2008;372(9641):801-3.

18. Pauliah SS, Shankaran S, Wade A, Cady EB, Thayyil S. Therapeutic hypothermia for neonatal encephalopathy in low- and middle-income countries: a systematic review and meta-analysis. PLoS One 2013;8:e58834.

19. Jary S, Whitelaw A, Walloe L, Thoresen M. Comparison of Bayley-2 and Bayley-3 scores at 18 months in term infants following neonatal encephalopathy and therapeutic hypothermia. Dev Med Child Neurol 2013.

20. Gorter JW, Ketelaar M, Rosenbaum P, Helders PJ, R P. Use of the GMFCS in infants with CP: the need for reclassification at age 2 years or older. Dev Med Child Neurol 2009;51:46-52.

21. Russell DJ, Avery LM, Rosenbaum PL, Raina PS, Walter SD, Palisano RJ. Improved scaling of the gross motor function measure for children with cerebral palsy: evidence of reliability and validity. Physical therapy 2000;80:873-85.

22. Russell DJ, Avery LM, Walter SD, et al. Development and validation of item sets to improve efficiency of administration of the 66-item Gross Motor Function Measure in children with cerebral palsy. Dev Med Child Neurol 2010;52:e48-54.

Peer review of the protocol for publication (Thayyil et al Hypothermia for encephalopathy in low and middle-income countries (HELIX): study protocol for a randomised controlled trial. Trials (2017) 18:432

Professor Martin Keszler
Associate Editor, Trials
Professor of Pediatrics
Brown University, USA

15.8.2017

Dear Professor Keszler

TRLS-D-17-00235: Hypothermia for Encephalopathy in Low and Middle-Income Countries (HELIX) trial

Please see our responses to the reviews comments below

Reviewer report:
Comment #1: Although I am not entirely familiar with resources typically available in the 3 South Asian countries involved, I am struck that all participating sites were selected because of their ability (among other things) to provide 'tertiary neonatal intensive care including cardiorespiratory support and monitoring'. While the selection of public facilities with NICU capability was intended to limit the inequities that inclusion of private sites with much more generous facilities, staff and resources might produce, I still question jst how generalizable the results of this study will be to other 'low income countries' that may have many fewer resources than the sites included in this study. I believe this should receive more emphasis.

We agree with the reviewer that the HELIX trials results will be generalizable only to middle-income country neonatal units with adequate facilities for neonatal intensive care. We believe that the safety and efficacy of cooling should be examined in these settings first, before cooling trials are conducted in low resource settings without adequate neonatal care facilities, for example in Sub Saharan Africa. If the HELIX trials suggest cooling improves outcomes, the next logical step would be a pragmatic cooling trial in Sub Saharan African settings, and in remote Indian villages.

The following paragraph is added to the discussion. Please see page 15, penultimate paragraph.

“The HELIX trial results will not generalizable to settings that lack good neonatal care, as in sub-Saharan Africa. If the HELIX trial demonstrates the safety and efficacy of cooling in middle-income country settings with reasonable intensive care facilities, the next logical step would be to conduct a large pragmatic trial of cooling in sub-Saharan Africa, and in rural Indian states, where neonatal intensive care facilities are not available”

2. How comparable is 'usual care' among the participating sites?
Differences in the usual care between the centres are minimal and primarily in the admission policies i.e. some centres admit only inborn babies and others only in out born babies. The effect of the place of delivery will be used as a confounder in exploratory analysis using regression models.

We have now included the following sentence in the discussion. Please see page 15, paragraph 4

"The participating centres have similar guidelines for usual care of encephalopathic babies, although some units admit only in-born babies, and others admit only out-born babies."

3. The importance of this study in assessing the role of perinatal infection on outcome is noted several times in the text, but very little/no information about how the data re: cultures, PCR and transcriptomics will be used in analyzing the findings are provided.

A detailed description of PCR and transcriptomic analysis is beyond the scope of the current HELIX protocol. We have now included a summary of the proposed infection workup and have included appropriate references for the readers to access the detailed protocols. Please see page 8 last two paragraphs and references 26 and 27

"Therefore, we will perform advanced molecular, histological and transcriptomic evaluation, in addition to standard automated blood cultures (Bactec), to identify any co-existent perinatal infection (Table 2)."

We will use Multiplex Real Time PCR (qPCR) on blood to identify babies with neonatal sepsis, using a panel of primers for both specific detection of common pathogens and generic detection. This will include Enterobacteriacae, Fusobacteria spps., Staphylococcus aureus, coagulase negative Staphylococci, and Streptococcus spps including Group A and B Streptococcus, Pneumococcus, and Peptostreptococcus spps), qPCR for quantitation of total 16s rDNA26. Our preliminary data suggests the vast majority of pathogens are gram negative, and unlike in high-income countries, group B streptococci is extremely rare in these settings.

Following RNA extraction from the whole blood collected in an RNA stabilising solution, we will perform Next Generation Sequencing (paired end) and alignment. The sequenced data will be examined for the established signatures of bacterial infections recently reported from Imperial College London27.

4. Can I assume that data re: maternal and neonatal antibiotic therapy that occurred before obtaining cultures, PCR and transcriptomics will be collected and used to help interpret the significance of these tests?

Blood samples for culture, PCR and transcriptomic will be collected prior to anti-biotics whenever possible. However, if the anti-biotics have been administered prior to blood sampling, this would be taken into consideration. We have now included this under the methods. Please see page 8, second paragraph

"Whenever possible, bloods will be collected before giving antibiotics. Babies who had antibiotics prior to the blood collection (for example babies referred from other hospitals) will be noted separately."

5. I do not know the practices in these 3 countries re: withdrawal of life support, but suggest that infants who die as a result of support withdrawal should be classified accordingly to potentially illuminate findings re: death as an outcome.

Withdrawal of life support is not legally permitted in any of the three countries. We have now included this under the discussion. Please see page 15, fourth paragraph.

"Unlike in high-income countries, withdrawal of life support is not legally permitted in the participating countries"

6. It is implied, but not specifically stated, in the text that infants with clinical seizures that occur in a setting consistent with perinatal asphyxia may also qualify for inclusion, even if encephalopathy is only mild. If this is so, this should be added to the inclusion criteria.

Mild encephalopathic babies with seizure will be classified as moderate encephalopathy, and this is already a part of the NICHD examination criteria. As the diagnosis of mild encephalopathy is more complicated, it is essential that the structured NICHD neurological examination need to be performed in every case to decide the stage of encephalopathy. We have now included this in the foot note of the NICHD examination table as well. Please see table 1 footnote.

“Infant who has seizure will be Moderate or Severe NE depending on the neurologic exam. Seizure with normal or mild NE or moderate NE on neurologic exam will be “Moderate NE”. Seizure with severe NE will be “Severe NE”.

7. Is it likely that infants may have received analgesics, sedatives or paralytics before the qualifying neurologic exam can be performed? If so, are such infants excluded, or is some accommodation made to account for the possible confounding effect of such therapy on the neurologic exam?

It is possible that some babies might have received analgesics/sedatives, and this will be recorded in the case record forms. But babies are extremely unlikely to have had muscle relaxants. Such babies will not be excluded as this is the real-life scenario, and HELIX is a pragmatic trial. A similar approach was used in the NICHD NRN cooling trial (Shankaran et al., NEJM 2005). We have now included the following sentence in the methods. Please see page 6, second paragraph.

“Sedatives or analgesics given prior to the neurological examination will be recorded in the case report form.”

8. Gestational age 36 weeks gestation is based on LMP or ultrasound examination. In the absence of an early ultrasound, how accurate is LMP likely to be in this population?

We agree that first trimester ultrasound may not be always available. However, given that the HELIX is a pragmatic trial in LMIC, and that there are not simple and accurate surrogate markers of gestational age we have decided to use only LMP, in addition to birthweight.

9. How will potential confounding events that occur after discharge from the NICU be handled? Given potential problems with nutrition, later infection, trauma and multiple other factors in low and middle countries that may impact outcomes among NICU survivors, this may be an important consideration.

These data will be collected. However, we do not expect these confounding factors are likely to be different in the intervention and control arms. We have now included the following sentence in the methods. Please see page 10, paragraph six

“We will also collect information of various morbidities and medical support required during infancy, and obtain detailed anthropometry to assess the nutritional status during the 18-month follow-up visit.”

10. How common is exclusion due to 'migrant family or parents unable/unlikely to come back for 18 month follow-up'? Might this result in significantly skewed population and outcomes?

Less than 5% is expected to be excluded due to difficulties in follow up, hence unlikely to skew the population. We have now included the following sentence in the exclusion criteria. Please see page 4, penultimate paragraph.

“Migrant family or parents unable/unlikely to come back for follow-up at 18 months (expected to be less than 5% of eligible population)”

11. Definitions of moderate and severe NDI at different places in the text are not concordant, e.g., moderate/severe at the bottom of page 4 versus that for severe near the bottom of page 5 versus those on page 9. In some places, ongoing seizure disorder qualifies as severe NDI and in others as moderate. In some places, 'blindness' qualifies, while in others 'impaired vision despite correction' qualifies. The definitions should be uniform throughput the manuscript.

We thank the reviewer for highlighting this error. We have now corrected this in the protocol (please see page 4 and page 5, last paragraph), and made the definitions of moderate and disability explicit and consistent. An amendment to the protocol will be submitted to the Ethics committee for the same.

“Severe disability was defined as any of the following: a Bayley III cognitive score of less than 70, a GMFCS level of 3 to 5, blindness, or profound hearing loss (inability to understand commands despite amplification).

Moderate disability was defined as a Bayley III cognitive score of 70 to 84 and either a GMFCS level of 2, seizure disorder, or a hearing deficit requiring amplification to understand commands”

12. In determining severe versus moderate NE, how is an assignment made if the infant has an equal number of moderate and severe abnormalities on the exam?

The assignment will be then based on the level of consciousness. We have now included the following sentences in the foot note of table 1.

The level of encephalopathy will be assigned based on which level of signs (moderate or severe) predominates among the 6 categories. If moderate and severe signs are equally distributed, the designation is then based on the highest level in Category #1: The level of consciousness. If the level of consciousness is equal, then designation of the NE stage based on the tone (Category # 4)

13. Is cooling stopped for an infant who may require surgery for some reason during the cooling period?

Cooling therapy will be discontinued in such cases. However, such scenario is unlikely, as babies with life threatening congenital malformations will not be recruited to the HELIX trial. We have now made this explicit in the exclusion criteria. Please see page 7, paragraph 4.

“Parent or clinician request to stop cooling therapy, for example if the baby requires any surgical procedure during first three days.”

14. What 'common' bacterial pathogens can be detected by the PCR and transcriptomic assays employed? Are there any pathogens important in the part of the world where the study is being conducted that might be missed, esp. among the gram-negatives?

We agree with the reviewer that gram negative are far more common pathogens in these settings than group B streptococci. Our PCR panel will cover for all common gram negative pathogens. Please see page 8, penultimate paragraph.

We will use Multiplex Real Time PCR (qPCR) on blood to identify babies with neonatal sepsis, using a panel of primers for both specific detection of common pathogens and generic detection. This will include Enterobacteriacae, Fusobacteria spps., Staphylococcus aureus, coagulase negative Staphylococci, and Streptococcus spps including Group A and B Streptococcus, Pneumococcus, and Peptostreptococcus spps), qPCR for quantitation of total 16s rDNA26. Our preliminary data suggests the vast majority of pathogens are gram negative, and unlike in high-income countries, group B streptococci is extremely rare in these settings.

15. Are follow-up phone calls a practical way to keep track of parents in this study population?

Mobiles phones are widely used in this population, and hence is an easy way to keep in contact with the family in these settings. We are also collecting the addresses and telephone numbers of extended family and grandparents in case of change of address or mobile number of parents. In addition, we will be using the existing community nurse equivalents (ASHA), to trace families.

16. Page 10, Adverse events: Given that death and adverse neurodevelopmental outcome at 18 months are among the main endpoints of the study, I am bit wary of stating that they are not 'intervention related'. This seems to imply that if cooling should be found to be associated with reduced death or NDI that it would not be attributed to cooling (the intervention).

We agree, and have now removed this sentence

17. While 'intention to treat' is definitely the primary approach to take for analysis of a pragmatic trial, it may still be useful to conduct a per protocol analysis as well, to help identify problem areas that may be useful to address going forward.

We agree, and both intention to treat and per protocol analysis will be performed. This is specified in the statistical analysis plan.

18. Page 12, bottom 2 lines: Are participating sites in Sri Lanka and Bangladesh not also expected to have local research ethics and other regulatory approvals as per local regulations?

We do have local research ethics approvals from Sri Lanka and Bangladesh, and this is now included in table 4, and in methods. Please see page 13, first paragraph

“Imperial College Research Ethics Committee (Central Ethics Committee for the HELIX trial) has approved the trial (ICREC Reference 15IC2564; 20 April 2015). The research ethics committee at approvals at the following recruiting centres have already been obtained – (Calicut Medical College, Kerala, India (IRC/2015/Protocol/57; 12 June 2015); Indira Gandhi Institute of Child Health, Bangalore, India (IEC01062015; 1 June 2015), Lokmanya Tilak Municipal Medical College staff and research society (IEC/30/15; 17 June 2015), Madras Medical College Institutional Ethics Committee (IEC08072015; 7 July 2015); Maulana Azad Medical College Institutional Ethics Committee (IEC/MAMC/52/1/2016; 30 March 2016). Bangabandhu Sheikh Mujib Medical University Institutional Review Board (BSMMU/2016/6885; 29.6.2016) and Ethics Review Committee, University of Kelaniya (P/109/03/2017;3.4.2017)”

Associate Editor comment:

This is a well-designed and important RCT that will inform the care for a large number of infants at high risk of poor outcome in under-resourced countries. I believe that the clarifications requested by the Reviewer will not only improve the current manuscript, but also help focus the data analysis and publication process in the long run.
MK

We thank the Associate Editor for the encouragement, and believe we have now provided all the clarifications requested by the reviewer.

We hope these revisions are satisfactory

Dr Sudhin Thayyil
Imperial College London

Published trial protocol

Thayyil *et al. Trials* (2017) 18:432
DOI 10.1186/s13063-017-2165-3

Trials

STUDY PROTOCOL Open Access

Hypothermia for encephalopathy in low and middle-income countries (HELIX): study protocol for a randomised controlled trial

Sudhin Thayyil[1*], Vania Oliveira[1†], Peter J. Lally[1†], Ravi Swamy[1], Paul Bassett[2], Mani Chandrasekaran[1], Jayashree Mondkar[3], Sundaram Mangalabharathi[4], Naveen Benkappa[5], Arasar Seeralar[6], Mohammod Shahidullah[7], Paolo Montaldo[1], Jethro Herberg[8], Swati Manerkar[3], Kumutha Kumaraswami[4], Chinnathambi Kamalaratnam[4], Vinayagam Prakash[6], Rema Chandramohan[4], Prathik Bandya[5], Mohammod Abdul Mannan[7], Ranmali Rodrigo[9], Mohandas Nair[10], Siddarth Ramji[11], Seetha Shankaran[12] and for the HELIX Trial group

Abstract

Background: Therapeutic hypothermia reduces death and disability after moderate or severe neonatal encephalopathy in high-income countries and is used as standard therapy in these settings. However, the safety and efficacy of cooling therapy in low- and middle-income countries (LMICs), where 99% of the disease burden occurs, remains unclear. We will examine whether whole body cooling reduces death or neurodisability at 18–22 months after neonatal encephalopathy, in LMICs.

Methods: We will randomly allocate 408 term or near-term babies (aged ≤ 6 h) with moderate or severe neonatal encephalopathy admitted to public sector neonatal units in LMIC countries (India, Bangladesh or Sri Lanka), to either usual care alone or whole-body cooling with usual care. Babies allocated to the cooling arm will have core body temperature maintained at 33.5 °C using a servo-controlled cooling device for 72 h, followed by re-warming at 0.5 °C per hour. All babies will have detailed infection screening at the time of recruitment and 3 Telsa cerebral magnetic resonance imaging and spectroscopy at 1–2 weeks after birth. Our primary endpoint is death or moderate or severe disability at the age of 18 months.

Discussion: Upon completion, HELIX will be the largest cooling trial in neonatal encephalopathy and will provide a definitive answer regarding the safety and efficacy of cooling therapy for neonatal encephalopathy in LMICs. The trial will also provide important data about the influence of co-existent perinatal infection on the efficacy of hypothermic neuroprotection.

Trial registration: ClinicalTrials.gov, NCT02387385. Registered on 27 February 2015.

Background

Every year, approximately one million babies die in low- and middle-income countries (LMIC) due to neonatal encephalopathy – a condition arising from an unexpected lack of cerebral blood flow and oxygen supply to the fetal brain at the time of birth [1]. Approximately one-third of infants with moderate or severe encephalopathy will die during the newborn period and up to three-quarters of survivors will develop long-term neurodisability [2, 3]. Until recently, there was no effective treatment for this condition and the management was limited to supportive care.

Several high-quality cooling trials have been conducted in high-income countries in the past decade [4–6]. The meta-analyses of these trials have convincingly demonstrated that selective head or whole-body cooling, along with optimal tertiary intensive care, reduces mortality (risk ratio [RR] 0.8; 95% confidence interval [CI] 0.7–0.9; $p = 0.005$) and improves survival with normal

* Correspondence: s.thayyil@imperial.ac.uk
†Equal contributors
[1]Centre for Perinatal Neuroscience, Imperial College London, London, UK
Full list of author information is available at the end of the article

BioMed Central

© The Author(s). 2017 **Open Access** This article is distributed under the terms of the Creative Commons Attribution 4.0 International License (http://creativecommons.org/licenses/by/4.0/), which permits unrestricted use, distribution, and reproduction in any medium, provided you give appropriate credit to the original author(s) and the source, provide a link to the Creative Commons license, and indicate if changes were made. The Creative Commons Public Domain Dedication waiver (http://creativecommons.org/publicdomain/zero/1.0/) applies to the data made available in this article, unless otherwise stated.

neurological outcome (RR 1.5; 95% CI 1.2–1.9; $p < 0.001$) after neonatal encephalopathy in these settings [7, 8]. The protective effect of cooling persists into later childhood [9, 10]. Whole-body cooling is now widely used as a standard therapy for encephalopathy in the UK and other high-income countries [11].

The safety and efficacy data on cooling therapy from high-income cooling trials cannot be extrapolated to LMICs, due to differences in population co-morbidities, particularly co-existent perinatal infection, growth restriction and meconium aspiration syndrome, and lack advanced cardio-respiratory intensive care facilities [12]. Preclinical data suggest that the brain injury is worse and the neuroprotective effect of hypothermia in neonatal encephalopathy is lost in the presence of co-existent infection, particularly with gram-negative organisms [13].

We systematically reviewed the published literature on the safety and efficacy of cooling therapy for neonatal encephalopathy due to hypoxia-ischemia in LMICs [14]. All published studies were small and/or of poor quality. Two studies reported increased mortality with cooling [15, 16]. A meta-analysis of all these trials showed a trend towards reduced mortality; however, this was not statistically significant (RR 0.74; 95% CI 0.4–1.3). More importantly, the CIs were wide and therefore significant benefits or harm could not be excluded. There were no data on long-term neurological follow-up after cooling therapy [14]. Thus, before cooling therapy is widely used as standard care therapy in LMICs, safety and efficacy data from adequately powered clinical trials are required.

Aims

Primary

To examine whether whole-body cooling to 33.5 °C, initiated within 6 h of birth and continued for 72 h, reduces death or moderate or severe neurodisability at 18–22 months after neonatal encephalopathy, in LMICs.

Secondary

- To examine if whole-body cooling reduces mortality at hospital discharge and at 18–22 months after neonatal encephalopathy
- To examine if whole-body cooling reduces moderate or severe neurodisability at 18–22 months in survivors after neonatal encephalopathy
- To examine if whole-body cooling reduces brain injury on magnetic resonance imaging (MRI) and spectroscopy performed during the neonatal period

Methods

This is a multi-country two arm unblinded pragmatic randomised controlled trial of whole-body cooling along with usual supportive care vs. usual care alone. We plan to recruit 408 babies with moderate or severe neonatal encephalopathy from public sector neonatal units in LMICs over a three-year period. We anticipate approximately 1200 babies will be screened for eligibility to achieve the required target.

The treatment duration (cooling therapy) is 72 h; however, the temperature of all recruited babies will be monitored during the first week after birth. Any temperature rise > 37.5 °C will be actively treated, both in the cooling and usual care arms, as fever increases brain injury and adverse outcomes after neonatal encephalopathy. The neurological outcomes will be assessed at 18–22 months of age. The trial duration will be five years, consisting of a staggered start-up period, 36 months of recruitment followed by a further 18 months of follow-up, and a final five months for data analysis and write-up.

Before the start of recruitment, the local clinical staff at the recruiting centres will have an intensive training on all aspects of the trial and research governance. All staff involved in the study will be required to maintain an up-to-date ICH-GCP certification.

Inclusion criteria

To ensure wider applicability of the trial intervention, we will use exclusive clinical criteria for selection of at risk infants. Although blood gas analysis and amplitude integrated electroencephalogram may be performed as a part of the clinical care, they will not form part of the eligibility criteria, as these investigations are not widely available in LMICs. Furthermore, accurate Apgar scores are often not available when babies are born at home or in other hospitals and then transferred to the recruiting centres, and often not collected beyond 5 min after birth. Hence, the inclusion criteria from the high-income country cooling trials are inappropriate for LMICs.

Therefore, our inclusion criteria are primarily based on a structured neurological examination using modified Sarnat staging.

Our preliminary data have shown that the HELIX inclusion criteria will identify most infants at high risk of adverse outcomes, without including infants with milder encephalopathy. To be eligible for recruitment, all three criteria below must be met:

1. age ≤ 6 h, birthweight ≥ 1.8 kg, gestation ≥ 36 weeks (based on reported last menstrual period or ultrasound);
2. need for continued resuscitation at 5 min of age and/or 5-min Apgar score < 6 (for babies born at hospital) or lack of crying by 5 min of age (for babies born at home);

3. Evidence of moderate or severe encephalopathy at < 6 h of age on a structured clinical examination based on modified Sarnat staging (Table 1).

Exclusion criteria

- Absent heart rate at 10 min of age despite adequate resuscitation or imminent death
- Major life-threatening congenital malformations
- Migrant family or parents unable/unlikely to come back for follow-up at 18 months (expected to be less than 5% of eligible population)

Outcome measures

Primary outcome measure

Severe disability was defined as any of the following: a Bayley III [17] cognitive score < 70; a Gross Motor Function Classification System (GMFCS) level of 3–5 [18]; blindness; or profound hearing loss (inability to understand commands despite amplification).

Moderate disability was defined as a Bayley III cognitive score of 70–84 and either a GMFCS level of 2, seizure disorder or a hearing deficit requiring amplification to understand commands.

Secondary outcome measures

Clinical outcomes (before discharge from hospital)

- Mortality from any cause before discharge from hospital
- Major intracranial haemorrhage on cranial ultrasound
- Gastric bleeds (fresh blood > 5 mL from nasogastric tube)
- Persistent hypotension (mean blood pressure < 25 mmHg despite maximum inotropic support)
- Pulmonary haemorrhage (copious bloody secretions with clinical deterioration requiring change(s) in ventilatory management)
- Persistent pulmonary hypertension (severe hypoxemia disproportionate to the severity of lung disease with a significant pre- and post-ductal saturation difference on pulse oximetry)
- Prolonged blood coagulation requiring blood products

Table 1 Modified Sarnat scale for clinical encephalopathy staging

Categories (total 6)	Signs of neonatal encephalopathy (NE) in each category			
	Normal	Mild NE	Moderate NE	Severe NE
1. Level of consciousness				
	Alert, responsive to external stimuli (state dependent, e.g. post feeds)	Hyper-alert, has a stare, jitteriness, high-pitched cry, exaggerated responds to minimal stimuli, inconsolable	Lethargic	Stupor/coma
2. Spontaneous activity				
	Changes position when awake	Normal or decreased	Decreased activity	No activity
3. Posture				
	Predominantly flexed when quiet	Mild flexion of distal joints (fingers, wrist usually)	Moderate flexion of distal joint, complete extension	Decerebrate
4. Tone				
	Strong flexor tone in all extremities + strong flexor hip tone	Normal or slightly increased peripheral tone	Hypotonia (focal or general) or hypertonia	Flaccid<br>Rigid
5. Primitive reflexes (circle only the highest level in each sign; the maximum score is only 1 in any one category)				
Suck	Strong, easily illicit	Weak, poor	Weak but has a bite	Absent
Moro	Complete	Partial response, low threshold to illicit	Incomplete	Absent
6. Autonomic system (circle only the highest level in each sign; the maximum score is only 1 in any one category)				
Pupils	In dark: 2.5–4.5 mm; in light: 1.5–2.5 mm	Mydriasis	Constricted	Deviation/dilated/non-reactive to light
Heart rate	100–160 bpm	Tachycardia (HR > 160)	Bradycardia (HR < 100)	Variable HR
Respiration	Regular respirations	Hyperventilation (RR > 60/min)	Periodic breathing	Apnoea or requires ventilator
Total score				

The level of encephalopathy will be assigned based on which level of signs (moderate or severe) predominates among the six categories. If moderate and severe signs are equally distributed, the designation is then based on the highest level in Category #1: The level of consciousness. If the level of consciousness is equal, then designation of the NE stage is based on the tone (Category #4). An infant who has seizures will be moderate or severe NE, depending on the neurologic exam. Seizure with normal or mild NE or moderate NE on neurologic exam will be 'Moderate NE'. Seizure with severe NE will be 'Severe NE'

- Culture-proven early onset sepsis (isolation/identification of a pathogenic organism from blood and/or cerebrospinal fluid along with clinical evidence of sepsis and elevation of C-reactive protein)
- Necrotising enterocolitis (abdominal distension, increased gastric aspirates and/or blood in stools, together with abdominal X-ray showing bowel oedema, pneumatosis or pneumoperitoneum, i.e. Bell's staging 2 or 3)
- Cardiac arrhythmia requiring therapy
- Severe thrombocytopenia (<25,000)
- Persistent metabolic acidosis lasting over 12 h after birth
- Renal failure (anuria > 48 h with azotaemia)
- Pneumonia (infiltrates on chest X-ray consistent with infection or aspiration)
- Subcutaneous fat necrosis
- Neurological examination at discharge
- Duration of hospitalisation

Neonatal cerebral magnetic resonance biomarkers

- Brain injury score on conventional MRI [19, 20]
- Proton magnetic resonance spectroscopy thalamic lactate/N-acetylaspartate peak area ratio and absolute concentration of N-acetylaspartate [21]
- Whole brain maps of diffusion tensor indices [22]

Neurodisability (18–22 months)

- Mortality
- Severe neurodevelopmental disability (severe disability was defined as any of the following: a Bayley III [17] cognitive score of < 70; a GMFCS level of 3–5 [18]; blindness; or profound hearing loss (inability to understand commands despite amplification)
- Microcephaly (head circumference more than 2 standard deviations below the mean)

Screening and neurological examination

All infants admitted to the neonatal unit with perinatal asphyxia will be screened for eligibility. Out-born babies meeting the inclusion criteria will be eligible for recruitment, irrespective of the temperature at admission to the neonatal unit (Additional file 1). Potentially eligible cases will have a detailed neurological examination by a designated neonatal doctor who is trained and accredited in NICHD neurological examination modified from the Sarnat staging (Table 1) [5]. Briefly, this scoring system assesses six categories of neurological symptoms. The highest score in each category will be recorded. The level of encephalopathy is determined based on the predominant degree of neurological abnormality (moderate or severe) across the six categories. Three out of the total six categories must be moderate or severe for the baby to be eligible.

Infants who have seizures will be considered to have moderate or severe neonatal encephalopathy depending on the neurological examination. Seizures combined with otherwise mild or moderate encephalopathy on neurological examination will be considered as moderate encephalopathy. Seizures combined with severe encephalopathy will be considered as severe encephalopathy. Sedatives or analgesics given before the neurological examination will be recorded in the case report form (CRF).

Treatment assignment and randomisation

As soon as informed parental consent is obtained for an eligible infant, the recruiting clinician will obtain the treatment assignment, which will be either 'usual care with cooling' or 'usual care only', using an Internet-based randomisation system (Sealed Envelope; https://www.sealedenvelope.com). Minimisation will be used to ensure balance between the groups with respect to the severity of encephalopathy at each centre. Further details are given in the statistical analysis plan.

Researchers will not be blinded to the intervention (cooling therapy). However, the neurological outcome evaluation at 18 months will be undertaken by assessors masked to the treatment allocation.

Cooling therapy

Therapeutic hypothermia will be administered using a servo-controlled whole-body cooling device, that has an effective cooling time (percentage of time for which the core body temperature is maintained within the target range of 33 °C to 34 °C) of over 90% [23]. Briefly, this would consist of attaching the mattress to the servo-controlled device, refilling coolant, keeping the baby on the mattress, placing a rectal probe, switching the machine on and selecting the appropriate program. Babies are kept on a radiant warmer with heating turned off and are not clothed, except for nappies. The cooling device will maintain the rectal temperature of the baby within 33 °C to 34 °C and will alarm when temperatures are out of this range (e.g. after displacement of the rectal probe). The clinical team will record rectal temperature hourly in the data collection form. In addition, the temperature data will be downloaded from the cooling device and compared with the manual records to ensure data entry accuracy. After 72 h of cooling, the baby will be automatically re-warmed at 0.5 °C per hour by the cooling device. Following re-warming all babies will have continuous rectal temperature monitoring until 90 h of age, and then every 8 h for the first week after birth.

Babies with a low temperature (rectal temperature < 35.5 °C) on admission to the neonatal unit

It is possible that rapid rewarming (>0.5 °C per hour) may result in increased brain injury and seizures. If these babies are already randomised to usual care and then kept under a servo-controlled radiant warmer set at 36.5 °C, rapid re-warming may occur in less than 1 h, thus worsening the outcome.

Hence, any baby admitted with a low rectal temperature (<35.5 °C) who is recruited to the usual care arm will be slowly rewarmed, at a rate not more than 0.5 °C per hour using the protocol below:

1. set the radiant warmer temperature 0.5 °C higher than the baby's rectal temperature and increase this by 0.5 °C every hour, until the rectal temperature reaches 36.5 °C;
2. if the radiant warmer servo-controlled mode fails, use manual mode keeping the heating output low (e.g. 20%), so that the baby's rectal temperature does not rise by more than 0.5 °C per hour;
3. if no radiant warmer available, keep the baby covered in warm clothes, so that the temperature slowly increases to 36.5 °C over several hours. This may take as long as 6–12 h.

Babies with a core temperature of > 37.5 °C during the first week after birth

All the major neonatal cooling trials to date had up to one-quarter of infants in the usual care having core temperatures > 37.5 °C. Subsequently, analysis of the trial data suggests that core temperatures above 37.5 °C adversely affect brain injury and worsen their outcome [24]. Therefore, any potential benefit seen with cooling may be spuriously related to the overheating of usual care babies. Moreover, a recent trial in adults have shown that 'therapeutic normothermia' is as effective as 'therapeutic hypothermia' following cardiac arrest [25].

Hence, we will use a more aggressive approach for preventing and treating elevated core temperatures in the usual care arm than the previous neonatal cooling trials. Fever may occur at any time in babies in the usual care arm and sometimes during the post-rewarming phase in the cooling arm. Babies allocated to usual care will be nursed on servo-control radiant warmer with a set temperature of 35.5 °C to 36 °C, to prevent any accidental overheating. Any rise in temperature > 37.5 °C, will be aggressively treated by turning off any radiant heater or warmer if in use, using fans, tepid sponging, antipyretics and, if necessary, using the servo-controlled cooling device in a 'normothermia mode'. In addition, any infective cause for fever will be investigated and treated, including lumbar puncture if appropriate.

Criteria for stopping cooling therapy

- Refractory hypotension (mean blood pressure < 25 mmHg) despite optimal inotropic and volume support
- Life-threatening/massive haemorrhage
- Parent or clinician request to stop cooling therapy, for example if the baby requires any surgical procedure during first three days

Supportive care and monitoring

The general management of babies will be standardised at the participating centres and will not permit therapies like steroids, mannitol or other experimental therapies in recruited infants.

Hourly vital signs (oxygen saturation, heart rate, non-invasive blood pressure, rectal temperature) will be recorded in the HELIX CRF in all infants. Additional monitoring will be dictated by the clinical condition and local guidelines.

Infants may also receive intravenous fluids, antibiotics, ventilatory support, inotropes, blood products, sedation, muscle relaxants and anti-convulsants as per the local clinical practice. The babies undergoing cooling therapy will also receive sedation, if they are ventilated or if there is any evidence of stress (for example, shivering, unexplained tachycardia).

Detailed neurological examination (using NICHD criteria) will be performed within 6 h of birth, then at day 3 and at discharge from hospital.

Baseline assessments and data collection

The following data points will be recorded in the CRF:

1. maternal (antenatal) and delivery details including resuscitation details;
2. time and date of birth, time of randomisation and start of cooling;
3. birthweight, gestation, gender and head circumference;
4. hourly rectal temperature profile in all infants for the first 90 h;
5. neurological examination within 6 h, at day 3 and at the time of discharge;
6. full blood count (including platelets, CRP and differential white cell count) within 6 h of birth and between days 4 and 7, as a part of clinical care;
7. Blood culture within 6 h of birth and between days 4 and 7;
8. biochemical series (including blood gas, sugar, urea, creatinine, electrolytes and coagulation profile) as a part of clinical care;
9. cranial ultrasound examination (within 72 h) to examine for major intracranial bleeds as a part of clinical care.

Each anonymised CRF will be scanned and emailed to the HELIX trial manager at Imperial College London for quality checks within 48 h of completion. The signed off CRFs will be entered into the HELIX trial database (Redcap®).

Screening for perinatal infection

Preclinical evidence suggests that co-existent bacterial infections, particularly those due to gram-negative bacteria, may negate the neuroprotective effects of hypothermia. Therefore, we will perform advanced molecular, histological and transcriptomic evaluation in addition to standard automated blood cultures (Bactec), to identify any co-existent perinatal infection (Fig. 1). Whenever possible, bloods will be collected before giving antibiotics. Babies who had antibiotics before the blood collection (for example, babies referred from other hospitals) will be noted separately.

This will include:

- blood for targeted polymerase chain reaction (qPCR) to transcriptomic signatures to detect common bacterial pathogens within 6 h of birth;
- a small section of the umbilical cord (fetal end) for histopathological examination for funisitis. In addition, a section from the placenta will be collected whenever feasible. An experienced perinatal pathologist will report histopathology, masked to the clinical data.

We will use multiplex real time PCR (qPCR) on blood to identify babies with neonatal sepsis, using a panel of primers for both specific detection of common pathogens and generic detection. This will include *Enterobacteriacae*, *Fusobacteria* spps., *Staphylococcus aureus*, coagulase-negative *Staphylococci*, and *Streptococcus* spps including Group A and B *Streptococcus*, *Pneumococcus* and *Peptostreptococcus* spps), qPCR for quantitation of total 16 s rDNA [26]. Our preliminary data suggest the vast majority of pathogens are gram-negative, and unlike in high-income countries, group B streptococci is extremely rare in these settings.

Following RNA extraction from the whole blood collected in an RNA stabilising solution, we will perform next generation sequencing (paired end) and alignment. The sequenced data will be examined for the established signatures of bacterial infections recently reported from Imperial College London [27].

Magnetic resonance imaging and spectroscopy

The treatment effect of cooling therapy on brain injury will be assessed and quantified using MRI and spectroscopy. All recruited infants (surviving beyond 1 week) will have an MR scan at 7–14 days of age. Preparation of the baby for MRI is given in Additional file 2.

All MR scans will be performed on 3 T scanners (Philips, Siemens or GE) using common harmonised sequences developed as a part of the Magnetic Resonance Biomarkers in Neonatal Encephalopathy (MARBLE) study [28]. 3 T MR scanning offers considerable benefits for MR spectroscopy, over 1.5 T imaging.

MR scanners will be calibrated using adult volunteer brain scans using the same sequences.

	STUDY PERIOD												
	Enrolment	Allocation	Post-allocation										Close-out
TIMEPOINT**	$-t_1$	0	Day 1	Day 2	Day 3	Day 4	Day 5	Day 7-14	Hospital discharge	3 m	6 m	12 m	18m to 22 m
ENROLMENT													
Antenatal history	X												
Birth history	X												
Postnatal history	X												
Neurological examination	X												
Eligibility screen	X												
Video recording of the consenting process	X												
Written informed consent	X												
Randomisation		X											
INTERVENTION (Age < 6 hours at start of intervention)													
Whole body cooling			←	—	→								
Usual care			X	X	X								
ASSESSMENTS													
Rectal temperature (1h)		X	X	X	X	X	X						
Blood gas analysis	X		X	X	X								
Full blood count	X					X							
Renal and liver function	X												
Coagulation screen	X												
Infection screen	X					X							
qPCR	X												
Transcriptomics	X												
Cranial ultrasound			X										
Neurological examination			X	X	X	X			X				
MRI and MR spectroscopy								X					
Telephone follow-up										X	X	X	
Neurodevelopmental assessment													X

NB: m=month; qPCR= quantitative polymerise chain reaction; MRI = magnetic resonance imaging

Fig. 1 Schedule of enrolment, intervention, and assessments

Conventional MRI protocol

- T1-weighted – 3D MPRAGE (1-mm isotropic resolution);
- T2-weighted – 2D axial TSE (0.5-mm in-plane resolution, 3-mm slices);
- Diffusion – 2D axial SE-EPI (2-mm isotropic resolution, b = 0.750 s mm^{-2}).

MR spectroscopy protocol

- Surveys pre- and post-spectroscopy for detection of gross motion;
- PRESS (15 × 15 × 15 mm^3, water suppressed, echo time = 288 ms) for metabolite peak area ratios;
- STEAM (15 × 15 × 15 mm^3, water suppressed, dual repetition time, echo time = 20 ms) for relaxation corrected metabolite signals;
- STEAM (15 × 15 × 15 mm^3, water unsuppressed, fixed repetition time, multiple echo times) for internal water referencing to provide metabolite concentrations.

The anonymised MR data will be encrypted and transferred to the Centre for Perinatal Neuroscience by Imperial College London file transfer protocols for analysis and storage. All MR data will be analysed centrally, masked to the allocation or other outcomes. Details of MR sequences are given in Additional file 3.

The raw spectroscopy data will be post-processed using software developed in-house at Imperial College London (Python v2.7) and an in-house implementation of TARQUIN will be used to calculate metabolite concentrations from the STEAM spectra. LCModel will be used to calculate metabolite peak area ratios from the PRESS spectra.

Diffusion imaging data will be processed using the FMRIB Software Library (FSL, v5.0) and registered across individuals using DTI-TK (http://dti-tk.sourceforge.net/pmwiki/pmwiki.php). The resulting maps of diffusion tensor indices will be compared between participants using FSL. Postnatal age and postmenstrual age at scan will be introduced as confounding variables into the general linear model rendering results age-independent.

MR biomarker endpoints

- thalamic N-acetylaspartate concentration determined from STEAM spectroscopy;
- thalamic lactate/N-acetylaspartate metabolite peak area ratio determined from PRESS spectroscopy;
- white matter fractional anisotropy determined from diffusion imaging using tract based spatial statistics;
- brain injury severity score determined from conventional MRI [20].

Follow-up and neurological assessments

The recruiting centres will maintain regular (3–6 months) telephone contact with parents to minimise attrition. The following information will be recorded at each contact:

1. general health status of the baby;
2. any change in home address or telephone number.

Each recruiting centre will have a dedicated and experienced neurodevelopmental paediatrician trained in Bayley Scales of Infant Development (Version III), who will assess the babies aged 18–22 months, masked to the allocation. Inter-observer variability will be addressed and corrected before the start of assessments, by comparing against a gold standard examiner (RS; Pearson's trainer for Bayley Scales of Infant Development (Version III). The Bayley scales will be administered in one of the local languages (mother tongue of the child) – Hindi, Marathi, Kannada, Tamil, Malayalam, Singhalese, Telugu or Bangla.

Detailed medical history, neurological examination and Gross Motor Function Classification System assessment will be also obtained during this visit using a predefined proforma and then entered into the HELIX trial database.

Severe disability will be defined as any one of the following: Bayley III cognitive composite score < 70; Gross Motor Function Classification System level 3–5; hearing impairment requiring hearing aids/cochlear implant; or blindness.

Moderate disability will be defined as cognitive composite score 70–84 and one or more of the following: Gross Motor Function Classification System level II; hearing impairment with no amplification/cochlear implant; or a persistent seizure disorder.

The examiner will feed back the results of the neurodevelopmental outcome tests to the parents, immediately after the assessment. A copy of this report will be provided to the local principal investigator for clinical management. We will also collect information of various morbidities and medical support required during infancy and obtain detailed anthropometry to assess the nutritional status during the 18-month follow-up visit.

Adverse events

All known adverse events relating to neonatal encephalopathy and cooling therapy are described in the parent information leaflet and will be part of obtaining the informed research consent, before the start of cooling therapy.

The following clinical events (1–9) occur due to the underlying disease (neonatal encephalopathy). Cooling trials from high-income countries have shown that cooling therapy reduces/does not increase the incidence of many of these clinical events in encephalopathic babies.

1. Death during neonatal period or during infancy
2. Brain injury observed with MRI
3. Adverse neurodevelopmental outcome at 18 months and at childhood
4. Persistent pulmonary hypertension
5. Metabolic imbalances
6. Cardiac arrhythmia
7. Renal failure
8. Coagulopathy
9. Gastric bleeds

Cooling therapy may increase the risk of the following adverse events noted in the previous randomised controlled trials.

1. Thrombocytopenia and increased need for platelet transfusions
2. Subcutaneous fat necrosis

All adverse events are expected to occur within the cooling period (first 72 h) or within 72 h of re-warming. Adverse reactions occurring subsequently (after one week of life), except subcutaneous fat necrosis, will not be considered as intervention related. Subcutaneous fat necrosis may occur several weeks after the therapy.

If an unexpected serious adverse event (SAE) occurs (i.e. an event not mentioned in the above list), it should be reported to the HELIX trial manager within 24 h, using one of the SAE report forms. The HELIX trial manager will ensure that the independent data monitoring committee (IDMC) and the research ethics committee (REC) are informed accordingly.

Statistical methods

The primary analysis will be a comparison of the infants assigned to usual care plus whole-body cooling with those infants assigned to usual care at randomisation (i.e. intention-to-treat analysis population), regardless of deviation from the protocol or whether they received the allocated intervention. Demographic factors, clinical characteristics and outcomes will be summarised with counts (percentages) for categorical variables, means (standard deviation [SD]) for normally distributed continuous variables or medians (interquartile [IQR] or entire range) for non-normally continuous variables. Further details are given in the statistical analysis plan.

In order to establish both the magnitude and direction of the effects of whole-body cooling intervention, comparative statistical analysis will entail calculating the RR plus 95% CI for the primary outcome. The chi-square test will be used to determine statistical significance, with a 5% significance level used.

Secondary outcomes will be evaluated using a 5% level of statistical significance, with 95% CIs reported, to take account of the number of outcomes analysed. The Chi-square test or Fisher's exact test will be used to analyse categorical outcomes with RRs reported with 95% CIs. The unpaired t-test will be used to analyse normally distributed continuous outcomes, with the mean difference (plus 95% CI) reported. Non-normally distributed continuous outcomes will be transformed to normality or alternatively analysed using the Mann–Whitney test. If the latter approach is used, the median difference (plus 95% CI) between groups will be reported.

Logistic regression will be used to perform an adjusted analysis for the primary outcome to investigate the impact of stratification/known prognostic factors including the stage of neonatal encephalopathy.

Analysis of secondary outcomes will be clearly delineated from the primary analysis in any statistical reports produced. Results will be reported according to the CONSORT statement.

The sample size is based on being able to detect a clinically significant 30% relative risk reduction in death or moderate/severe disability from 50% in the usual care arm to 35% in the intervention (cooled) arm. Using a two-sided 5% significance level and an 80% power, 183 babies per arm are required. Assuming a loss to follow-up rate of around 10%, this comparison requires 204 babies per group, 408 babies in total, to be recruited. In case the adverse outcomes (death and moderate/severe disability) are higher (~65%) in the usual care arm, then this sample size would provide 94% power to detect a 30% relative risk reduction with cooling.

Each CRF (pdf) will be send to the HELIX trial manager within 48 h of discharge/death and all data queries will be resolved in real time by the HELIX research nurse/data entry person. The CRF will be signed off for completion and entered into the HELIX Redcap database by a dedicated data entry person at Imperial College London. The HELIX trial manager will then forward the trial data to an independent statistician, after masking the allocation (as X and Y) for analysis. The trial statistician will analyse the data for IDMC meetings as per a predefined proforma (masked as A and B), at six-monthly intervals or after recruitment of every 50 cases whichever is earlier. The temperature data will be reported to the IDMC as adherence to target temperature, rather than actual temperatures to prevent

unmasking of the IDMC members. The principal investigators will not have access to the trial data before locking, nor will they have any role in the data analysis. The trial CONSORT diagram is given in Fig. 2.

Trial organisation

Independent Data Monitoring Committee (IDMC)

An IDMC will review the study's progress. The IDMC will be independent of the trial organisers. The IDMC will meet every six months or after recruitment of 50 babies, whichever is earlier.

Meetings of the committee will be arranged periodically, as considered appropriate by the Chair.

The IDMC will inform the Trial Steering Committee (TSC) if in their view:

- there is proof beyond reasonable doubt that the data indicate that any part of the protocol under investigation is either clearly indicated or contra-indicated, either for all infants or for a subgroup of trial participants;
- it is evident that no clear outcome will be obtained;
- safety signal.

The primary endpoint will be assessed 18 months after the intervention has been performed. Given the planned period of recruitment, the primary endpoint will not be able to be assessed until majority of patients have been recruited. Thus, it will not be possible to stop the study early based on the study outcomes.

The membership of IDMC is detailed below:

- Professor Abbot Laptook (Chair, Professor for Neonatology, Brown University, USA)
- Professor Shabbar Jaffar (Faculty of Epidemiology and Population Health, London School of Hygiene and Tropical Medicine, London, UK)
- Professor Niranjan Thomas (Professor of Neonatology, Christian Medical College, Vellore, India)
- Dr Aung Soe (Consultant Neonatologist, Medway Hospital NHS Trust, Kent, UK)

The HELIX trial statistician will provide the trial data for IDMC meetings according to a pre-defined reporting format agreed by the IDMC.

Trial Steering Committee

The TSC will provide overall supervision of the study of the Sponsor. Its terms of reference are:

1. to monitor and supervise the progress of the HELIX trial towards its interim and overall objectives;
2. to review at regular intervals relevant information from other sources (e.g. related studies);
3. to consider the recommendations of the IDMC.

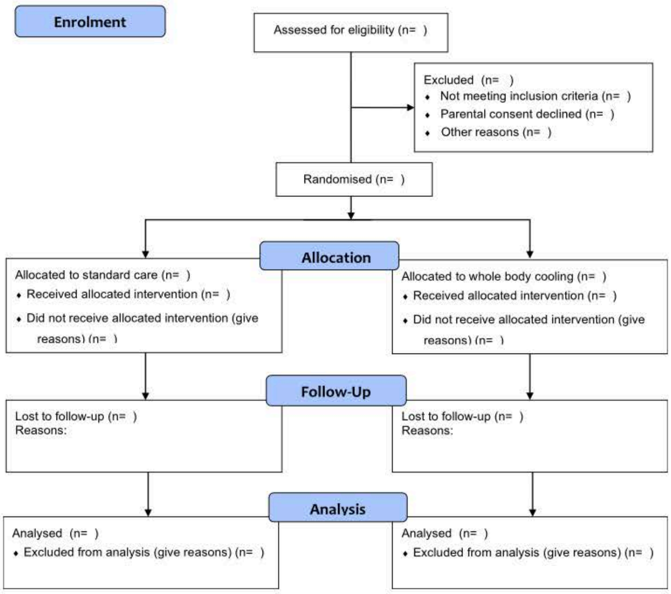

Fig. 2 CONSORT *diagram*

Project Management Group

The Project Management Group will oversee all aspects of the day-to-day running of the study and will consist of the investigators and the HELIX trial staff, based at the HELIX Co-ordinating Centre at Madras Medical College, Chennai, India and the Centre for Perinatal Neuroscience, Imperial College London. The Project Management Group will hold a monthly teleconference of all HELIX investigators for the entire duration of the trial to discuss the data quality and recruitment.

All correspondence with the REC will be retained in the Trial Master File/Investigator Site File. Annual reports will be submitted to the REC in accordance with Imperial College London requirements. It is the Chief Investigator's responsibility to produce the annual reports as required. Each participating site in India will have local research ethics and other regulatory approvals as per the local regulations.

Consent

The clinical team at each centre will explain the study to the parents, provide study information leaflets (in the appropriate local language – Kannada, Hindi, Tamil, Telugu, Marathi, Bengali, Singhalese or Malayalam) and will seek written informed consent. The entire consenting processes (including explanation of the study to parents) will be video-recorded. The digital video recordings will be securely stored at the local centre and at Imperial College London. The attending physician will regularly meet with parents during the intervention period to ensure that they understand the study procedures, throughout the course of hospital stay.

Each participant's right to refuse or withdraw from the study without giving reasons will be respected at all times. A withdrawal form will be filled in and authorisation will be obtained for use of the previously collected data.

The site principal investigator will retain the original of each patient's signed informed consent form. Copies of the information leaflet and consent form will be provided to the parents and kept in the medical records.

Declaration of Helsinki and Good Clinical Practice

The trial will be performed in accordance with the declaration of Helsinki, the conditions and principles of Good Clinical Practice, the protocol and applicable local regulatory requirements and laws. None of the investigators have any financial or competing interests in the trial results.

Data protection and patient confidentiality

All investigators and trial site staff involved in this trial must comply with the requirements of the UK Data Protection Act 1998 and local policy (India, Bangladesh and Sri Lanka) with regards to the collection, storage, processing and disclosure of personal information and will uphold the Act's core principles. The site investigators at each site will ensure that only linked anonymised data are received by the HELIX trial team.

Hard copies of CRFs and consent forms will be stored inside a locked cupboard in a designated HELIX research office at each recruiting centre, under the supervision of the site principal investigator. Only the HELIX research nurse, research doctor, data entry clerk and principal investigator at each site will have access to individual hard copies of the CRFs. Linked anonymised electronic data will be stored in a GCP-compliant secure UK-based server, with daily back-up. The HELIX investigators will have access to the electronic trial data only after the data are locked for the final analysis.

All trial staff must hold evidence of appropriate GCP training or undergo GCP training before undertaking any responsibilities on this trial. This training should be updated every two years or in accordance with the Sponsor's policy.

Sponsorship, financial and insurance

The HELIX feasibility trial and the setup of the HELIX trial was funded by the Bill & Melinda Gates Foundation (OPP1069985). The main HELIX trial is funded as a part of a Weston Garfield Chair Endowment Grant (Imperial College London) to Dr Thayyil. The cooling devices (Tecotherm Neo) are provided by Inspiration Health Care, UK, on loan to the recruiting sites.

The trial is sponsored by Imperial College London. Imperial College London will arrange insurance for negligent harm caused because of protocol design and for non-negligent harm arising through participation in the clinical trial.

None of the funders or sponsors have will have any role in the study design, analysis, interpretation or publication of the results.

Monitoring, audit and inspection

The site principal investigator must make all trial documentation and related records available for the monitoring by the study team and by the Sponsor. All patient data will be handled and treated confidentially.

The study team's monitoring frequency will be determined by an initial risk assessment performed before the start of the trial. A detailed monitoring plan will be generated detailing the frequency and scope of the monitoring for the trial. Throughout the course of the trial, the risk assessment will be reviewed and the monitoring frequency adjusted as necessary.

Protocol compliance and breaches of GCP

Prospective, planned deviations or waivers to the protocol are not allowed under the UK regulations on clinical trials and must not be used. For example, it is not acceptable to enrol a participant if they do not meet one or more eligibility criteria (for example, babies with mild encephalopathy or no encephalopathy) or restrictions specified in the trial protocol.

Protocol deviations, non-compliances or breaches are departures from the approved protocol. They can happen at any time, but are not planned. They must be adequately documented on the relevant forms and reported to the chief investigator and sponsor immediately. Deviations from the protocol which are found to occur repeatedly will not be accepted, will require immediate action and could potentially be classified as serious breaches. Any potential/suspected serious breaches of GCP must be reported immediately to the Sponsor without any delay. The SPIRIT checklist is provided as an Additional file 4.

Discussion

Upon completion, the HELIX trial will provide the most comprehensive data on safety and efficacy of cooling therapy in LMICs. Furthermore, it will provide definitive answers into the interaction of perinatal asphyxia and infection and how this relates to brain injury and neuroprotection in neonatal encephalopathy.

The HELIX trial is unique in several aspects. First, it is a pragmatic trial conducted in the real-life scenario of public sector tertiary neonatal units in LMICs. Cooling therapy will be provided using existing clinical staff in these units and research nurses (if available) will only be involved in collection of the research data. Moreover, HELIX uses exclusive clinical inclusion criteria and examines important clinical outcomes such as death and disability.

The participating centres in HELIX were carefully selected based on the following criteria: burden of encephalopathy (neonatal encephalopathy admission rate > 200 and/or delivery rate > 15,000 per year); availability of tertiary neonatal intensive care including cardiorespiratory support and monitoring; feasibility of long term follow-up; and adequate data quality during the HELIX feasibility trial phase. Advanced intensive care support such as 1:1 nursing care, invasive blood pressure monitoring, nitric oxide and extra-corporeal membrane oxygenator therapy will not be available. Thus, the trial results would be applicable to the vast majority of public sector neonatal units in LMIC. The participating centres have similar guidelines for usual care of encephalopathic babies, although some units admit only in-born babies and others admit only out-born babies. Unlike in high-income countries, withdrawal of life support is not legally permitted in the participating countries.

Substantial inequality in healthcare, both in terms of access and available resources, exists in LMICs. For example, hospitals in the private/cooperate sector (for profit) cater to relatively higher economic strata who can afford to pay for their healthcare and are well resourced. However, these hospitals have lower delivery rates (typically 500–3000 per year) and a very low burden from neonatal encephalopathy. In contrast, publicly funded hospitals (not-for-profit) offer free healthcare to patients from lower economic strata, but are under-resourced and have heavy disease (encephalopathy) burden. The HELIX trial will assess the safety and efficacy of cooling therapy in these socially and economically disadvantaged groups of babies; hence, we will recruit only from publicly funded hospitals (not-for-profit) catering to a low-income population. Thus, the HELIX trial is expected to reduce healthcare inequalities in LMICs.

The HELIX trial results will not be generalisable to settings that lack good neonatal care, as in sub-Saharan Africa. If the HELIX trial demonstrates the safety and efficacy of cooling in middle-income country settings with reasonable intensive care facilities, the next logical step would be to conduct a large pragmatic trial of cooling in sub-Saharan Africa and in rural Indian states, where neonatal intensive care facilities are not available.

Second, given the huge burden of neonatal encephalopathy in LMICs, a modest benefit from cooling therapy on a sub-group of encephalopathic infants, might have a substantial health impact. Hence, several additional state-of-art bacteriological, transcriptomic and advanced neuroimaging investigations will be performed as a part of the research protocol, to examine the interactions of perinatal infection on hypothermic neuro-protection.

We used optimised cross-platform 3 T MR sequences (developed at Imperial College London) so that the data from the three different MR scanner makes (Phillips, Siemens and GE) at the recruiting centres can be pooled together. Before the start of recruitment, we performed extensive harmonisation of the MR scanners and compared the spectroscopy metabolites on the same adult volunteer who travelled to all recruiting different sites. Once completed, HELIX would be the largest trial to use MR spectroscopy biomarkers in neonatal encephalopathy.

Although, none of the advanced investigations (including MRI) would be required in routine clinical practice of cooling therapy, this would provide valuable insights into the underlying disease mechanisms.

Table 2 Recruitment details

No.	Centre name	Open for recruitment	First case recruited	Total cases recruited	MR scanner	Status
1	Indira Gandhi Institute of Child Health, Bangalore, India	15 Aug 2015	16 Aug 2015	63	3 T Siemens Skyra	Recruiting
2	Institute of Child Health, Madras Medical College, Chennai, India	15 Aug 2015	25 Aug 2015	93	3 T Siemens Skyra	Recruiting
3	Lokmanya Tilak Municipal Medical College, Mumbai, India	31 Aug 2015	5 Sept 2015	40	3 T Phillips Achieva	Recruiting
4	Maulana Azad Medical College, New Delhi, India	On hold[a]	NA	0	3 T Siemens Skyra	Lack of bed space in neonatal unit
5	Calicut Medical College, Kerala, India	Withdrawn[b]	NA	0	1.5 T GE	Withdrawn due to lack of 3 T MRI availability
6	Institute of Obstetrics and Gynaecology, Madras Medical College, Chennai, India	1 Jan 2017	4 Jan 2017	18	3 T Siemens Skyra	Recruiting
7	Bangabandhu Sheikh Mujib Medical University, Dhaka, Bangladesh	7 June 2017	12 July 2017	3	3 T Siemens Skyra	Recruiting
8	University of Kelaniya, Sri Lanka	9 May 2017	24 May 2017	5	3 T Siemens Skyra	Recruiting

[a]Due to substantial increase in hospital deliveries in the hospital since 2016, newborn infants requiring neonatal intensive care unit are having to wait in postnatal wards for several hours before admission. This has prevented research recruitment and, hence, the trial is on hold at this centre
[b]This centre has been withdrawn from recruitment to HELIX due to lack of 3 T MR scanner

Publications policy

Ownership of the data arising from this trial resides with the trial team. On completion of the trial, the data will be analysed and tabulated and a final study report prepared. Consort guidelines and checklist will be reviewed by the trial steering committee before generating any publications for the trial to ensure they meet the standards required for submission to high-quality peer-reviewed journals, etc. (http://www.consort-statement.org). Sub-studies including the HELIX trial data will be published only after the publication of the main trial results.

A copy of the study results will be also given to the parents of all recruited babies, if they wish. This will be recorded at the time of recruitment and again during follow-up. The Sponsor and funders will have no role in the study management, analysis and interpretation of data, writing of the report or the decision to submit the report for publication.

Table 3 Amendments to the original protocol

Date	Amendment
20 Apr 2015	Original ethics approval (protocol version 1.1)
14 Jul 2015	Amendment 1: (Protocol version 1.2)[a]<br>(1) Clarification of the statistical analysis plan in the protocol as suggested by the Independent Data Safety Monitoring Committee<br>(2) Making the role of funders explicit in the protocol<br>(3) Including publication plans in the protocol
24 Jun 2016	Amendment 2: (protocol version 3)<br>(1) Use of telephone consent if parents are not present at the time of admission to the neonatal unit<br>(2) Inclusion of centres in Bangladesh and Sri Lanka<br>(3) Sending first birthday cards to the recruited babies
12 Aug 2017	Amendment 3: (Protocol version 3.1)<br>(1) Discrepancies in the categorisation of moderate and severe disability in the protocol corrected

[a]Amendment 1 was obtained before recruitment of the first case (16 August 2015)

Trial progress

The first patient was recruited on 16 August 2015. We have recruited a total of 222 babies at the time of protocol publication (Table 2). The trial is expected to complete recruitment by August 2018.

Additional files

Additional file 1: Screening flowchart. (PDF 196 kb)
Additional file 2: Preparation for magnetic resonance imaging. (PDF 303 kb)
Additional file 3: Magnetic resonance (3 Tesla) protocol. (PDF 821 kb)
Additional file 4: SPIRIT checklist. (PDF 77 kb)

Abbreviations

CRP: C-reactive protein; GMFCS: Gross Motor Function Classification System; IDMC: Independent Data Monitoring Committee; LMIC: Low and middle-income countries; MPRAGE: Magnetization-prepared rapid gradient-echo; MRI: Magnetic resonance imaging; MRS: Magnetic resonance spectroscopy; PRESS: Point-resolved spectroscopy; REC: Research Ethics Committee; SAE: Serious adverse event; SE EPI: Spin-echo echo-planar imaging; STEAM: Stimulated echo acquisition mode; TSC: Trial steering committee; TSE: Turbo spin echo

Acknowledgements

We thank Neil Campbell (Chief Executive Officer, Inspiration Health Care, UK) for providing the cooling machines for the trial.
Helix Trial group
Wayne State University, United States of America: Seetha Shankaran; Imperial College London: Gaurav Atreja, Mani Chandrasekaran, Jethro Herberg, Pete Lally, Josephine Mendoza, Paolo Montaldo, Vania Oliveira, Ravi Swamy, Sudhin Thayyil; Great Ormond Street hospital for Children and University College Hospital, London: Neil Sebire, Nigel Klein, Kshitij Mankad; Bangabandhu Sheikh Mujib Medical University, Dhaka, Bangladesh: Arjun Chandra Dey, Sanjoy Kumer Dey, Mohammed Tariqul Islam, Ismat Jahan, Mohammed Abdul Mannan,

Sadeka Chowdhury Moni, Kamrul Hasan Shabuj, Mohammod Shahidullah; National Institute of Neurosciences & Hospital, Dhaka, Bangladesh: Mohammed Nazrul Islam, Mst. Nazmun Nahar; Maulana Azad Medical College, New Delhi, India: Ashish Jain, Siddarth Ramji; Lokmanya Tilak Municipal Medical College, Sion, Mumbai, India: Swati Manerkar, Jayashree Mondkar, Kapil Dewang, Swapnil Bhiskar; Institute of Child Health, Egmore, Madras Medical College, Chennai, India: Chinnathambi Kamalaratnam, Kumutha Kumaraswami, Sundaram Mangalabharathi, Padmesh Vadekepad, Monica Sebastian; Indira Gandhi Institute of Child health, Bangalore, India: Naveen Benkappa, Prathik Bandya, Usha Kantharajanna, Sowmya Krishnappa, Jagdish Somanna, Niranjan Hunsanhalli Shivanna; Institute of Obstetrics & Gynecology, Madras Medical College, Chennai, India: Arasar Seeralar, Vinayagam Prakash, Mythilli Babu, Mohamed Sajjid; Radiology, Madras Medical College, Chennai, India: Babu Peter Sathyanathan, Kailasanathan Natarajan, Senthil Kumaran; Microbiology, Madras Medical Mission, Chennai, India: Anusha Rohit; Nitte University Centre for Science Education & Research, Mangalore: Indrani Karunasagar; Institute of Maternal and Child Health, Government Medical College Calicut, India: Vijaykumar Madhavan, Mohandas Nair; University of Kelaniya, Sri Lanka: Ranmali Rodrigo, Shaman Rajindrajith, Jithangi Wanigasinghe, Samanmali Sumanasena, Radika Karunaratne, Sanjeewa Munasinghe, Kalpani Chathirangika.

Funding
The trial is funded by Weston Garfield Foundation and the Bill & Melinda Gates Foundation. The funding bodies have no role in the trial design or interpretation of the data. The content is solely the responsibility of the authors and does not necessarily represent the official views of the funding bodies.

Availability of data and materials
Once the main findings of the project have been published, the trial steering committee will review all requests for data before access is granted. If appropriate, we will make the anonymized data and associated documentation available to users under a data-sharing agreement.

Authors' contributions
ST, SR and SS conceived and designed the study, obtained funding and are responsible for all aspects of the trial. VO led the trial management and PL led MRI and spectroscopy. RS, SM and RC are responsible for neurodevelopmental outcome assessments. PaB is responsible for statistical analysis. MC, JM, SMB, NB, AS, MS, KK, CK, VP, PrB, VM, MN, MAM and RR led the site-specific recruitments and carried out the study interventions. PM and JH contributed to the transcriptomic analysis and infection part of the study. All authors read and approved the final manuscript.

Ethics approval and consent to participate
Imperial College Research Ethics Committee (Central Ethics Committee for the HELIX trial) has approved the trial (ICREC Reference 15IC2564; 20 April 2015). The REC approvals at the following recruiting centres have already been obtained: Calicut Medical College, Kerala, India (IRC/2015/Protocol/57; 12 June 2015); Indira Gandhi Institute of Child Health, Bangalore, India (IEC01062015; 1 June 2015); Lokmanya Tilak Municipal Medical College staff and research society (IEC/30/15; 17 June 2015); Madras Medical College Institutional Ethics Committee (IEC08072015; 7 July 2015); Maulana Azad Medical College Institutional Ethics Committee (IEC/MAMC/52/1/2016; 30 March 2016); Bangabandhu Sheikh Mujib Medical University Institutional Review Board (BSMMU/2016/6885; 29 June 2016); and Ethics Review Committee, University of Kelaniya (P/109/03/2017; 3 April 2017) (Table 3). The Health Ministry's Screening Committee (HMSC) of India and the Indian Council of Medical Research (ICMR) have also approved the proposal. All original trial documentation and any subsequent amendments will be approved by the Sponsor (Imperial College London) and by the relevant ethical bodies, before their implementation. Informed written parental consent will be obtained from parents of next kin of recruited babies and the consenting process will be video-recorded.

Consent for publication
Written informed consent was obtained from the parents for publication of their individual details and any accompanying images in this manuscript. The consent form is held by the authors and in the patients' clinical notes and is available for review by the Editor-in-Chief.

Competing interests
The authors declare that they have no competing interests.

Publisher's Note
Springer Nature remains neutral with regard to jurisdictional claims in published maps and institutional affiliations.

Author details
[1]Centre for Perinatal Neuroscience, Imperial College London, London, UK. [2]Stats Consultancy, Amersham, Buckinghamshire, UK. [3]Lokmanya Tilak Municipal Medical College, Sion, Mumbai, India. [4]Institute of Child Health, Egmore, Madras Medical College, Chennai, India. [5]Indira Gandhi Institute of Child health, Bangalore, India. [6]Institute of Obstetrics & Gynecology, Madras Medical College, Chennai, India. [7]Neonatal Medicine, Bangabandhu Sheikh Mujib Medical University, Dhaka, Bangladesh. [8]Paediatric Infectious Diseases, Imperial College London, London, UK. [9]University of Kelaniya, Kelaniya, Sri Lanka. [10]Institute of Maternal and Child Health, Government Medical College Calicut, Calicut, India. [11]Maulana Azad Medical College, New Delhi, India. [12]Neonatal-Perinatal Medicine, Wayne State University, Detroit, MI, USA.

Received: 24 March 2017 Accepted: 29 August 2017
Published online: 18 September 2017

References
1. Lawn JECS, Zupan J, for the Lancet Neonatal Survival Steering Team. 4 million neonatal deaths: When? Where? Why? Lancet. 2005;365:891–900.
2. Pierrat VHN, Liska A, Thomas D, Subtil D, Truffert P, Groupe d'Etudes en Epidemiologie Perinatale. Prevalence, causes, and outcome at 2 years of age of newborn encephalopathy: population based study. Arch Dis Child Fetal Neonatal Ed. 2005;90(3):F257–61.
3. Robertson CM, Perlman M. Follow-up of the term infant after hypoxic-ischemic encephalopathy. Paediatr Child Health. 2006;11(5):278–82.
4. Azzopardi D, Strohm B, Edwards A, Dyet L, Halliday H, Juszczak E, et al. Moderate hypothermia to treat perinatal asphyxial encephalopathy. N Engl J Med. 2009;361(14):1349–58.
5. Shankaran S, Laptook AR, Ehrenkranz RA, Tyson JE, McDonald SA, Donovan EF, et al. Whole-body hypothermia for neonates with hypoxic-ischemic encephalopathy. N Engl J Med. 2005;353(15):1574–84.
6. Gluckman PD, Wyatt JS, Azzopardi D, Ballard R, Edwards AD, Ferriero DM, et al. Selective head cooling with mild systemic hypothermia after neonatal encephalopathy: multicentre randomised trial. Lancet. 2005;365(9460):663–70.
7. Edwards AD, Brocklehurst P, Gunn AJ, Halliday H, Juszczak E, Levene M, et al. Neurological outcomes at 18 months of age after moderate hypothermia for perinatal hypoxic ischaemic encephalopathy: synthesis and meta-analysis of trial data. BMJ. 2010;340:c363.
8. Jacobs SE, Berg M, Hunt R, Tarnow-Mordi WO, Inder TE, Davis PG. Cooling for newborns with hypoxic ischaemic encephalopathy. Cochrane Database Syst Rev. 2013;1:CD003311.
9. Azzopardi D, Strohm B, Marlow N, Brocklehurst P, Deierl A, Eddama O, et al. Effects of hypothermia for perinatal asphyxia on childhood outcomes. N Engl J Med. 2014;371(2):140–9.
10. Shankaran S, Pappas A, McDonald SA, Vohr BR, Hintz SR, Yolton K, et al. Childhood outcomes after hypothermia for neonatal encephalopathy. N Engl J Med. 2012;366(22):2085–92.
11. Azzopardi D, Strohm B, Linsell L, Hobson A, Juszczak E, Kurinczuk JJ, et al. Implementation and conduct of therapeutic hypothermia for perinatal asphyxial encephalopathy in the UK–analysis of national data. PLoS One. 2012;7(6):e38504.
12. Montaldo P, Pauliah SS, Lally PJ, Olson L, Thayyil S. Cooling in a low-resource environment: lost in translation. Semin Fetal Neonatal Med. 2015;20(2):72–9.
13. Osredkar D, Thoresen M, Maes E, Flatebo T, Elstad M, Sabir H. Hypothermia is not neuroprotective after infection-sensitized neonatal hypoxic-ischemic brain injury. Resuscitation. 2014;85(4):567–72.
14. Pauliah SS, Shankaran S, Wade A, Cady EB, Thayyil S. Therapeutic hypothermia for neonatal encephalopathy in low- and middle-income countries: a systematic review and meta-analysis. PLoS One. 2013;8(3):e58834.
15. Thayyil S, Shankaran S, Wade A, Cowan FM, Ayer M, Satheesan K, et al. Whole-body cooling in neonatal encephalopathy using phase changing material. Arch Dis Child Fetal Neonatal Ed. 2013;98(3):F280–1.

16. Robertson NJ, Nakakeeto M, Hagmann C, Cowan FM, Acolet D, Iwata O, et al. Therapeutic hypothermia for birth asphyxia in low-resource settings: a pilot randomised controlled trial. Lancet. 2008;372(9641):801–3.
17. Jary S, Whitelaw A, Walloe L, Thoresen M. Comparison of Bayley-2 and Bayley-3 scores at 18 months in term infants following neonatal encephalopathy and therapeutic hypothermia. Dev Med Child Neurol. 2013;55(11):1053–9.
18. Wood E, Rosenbaum P. The gross motor function classification system for cerebral palsy: a study of reliability and stability over time. Dev Med Child Neurol. 2000;42(5):292–6.
19. Shankaran S, Barnes PD, Hintz SR, Laptook AR, Zaterka-Baxter KM, McDonald SA, et al. Brain injury following trial of hypothermia for neonatal hypoxic-ischaemic encephalopathy. Arch Dis Child Fetal Neonatal Ed. 2012;97(6):F398–404.
20. Shankaran S, McDonald SA, Laptook AR, Hintz SR, Barnes PD, Das A, et al. Neonatal magnetic resonance imaging pattern of brain injury as a biomarker of childhood outcomes following a trial of hypothermia for neonatal hypoxic-ischemic encephalopathy. J Pediatr. 2015;167(5):987–93 e3.
21. Thayyil S, Chandrasekaran M, Taylor A, Bainbridge A, Cady E, Chong K, et al. Cerebral magnetic resonance biomarkers in neonatal encephalopathy: a meta-analysis. Pediatrics. 2010;125(2):e382–95.
22. Lally PJ, Price DL, Pauliah SS, Bainbridge A, Kurien J, Sivasamy N, et al. Neonatal encephalopathic cerebral injury in South India assessed by perinatal magnetic resonance biomarkers and early childhood neurodevelopmental outcome. PLoS One. 2014;9(2):e87874.
23. Robertson NJ, Kendall GS, Thayyil S. Techniques for therapeutic hypothermia during transport and in hospital for perinatal asphyxial encephalopathy. Semin Fetal Neonatal Med. 2010;15(5):276–86.
24. Laptook AR, McDonald SA, Shankaran S, Stephens BE, Vohr BR, Guillet R, et al. Elevated temperature and 6- to 7-year outcome of neonatal encephalopathy. Ann Neurol. 2013;73(4):520–8.
25. Moler FW, Silverstein FS, Holubkov R, Slomine BS, Christensen JR, Nadkarni VM, et al. Therapeutic hypothermia after in-hospital cardiac arrest in children. N Engl J Med. 2017;376(4):318–29.
26. Tann CJ, Nkurunziza P, Nakakeeto M, Oweka J, Kurinczuk JJ, Were J, et al. Prevalence of bloodstream pathogens is higher in neonatal encephalopathy cases vs. controls using a novel panel of real-time PCR assays. PLoS One. 2014;9(5):e97259.
27. Herberg JA, Kaforou M, Wright VJ, Shailes H, Eleftherohorinou H, Hoggart CJ, et al. Diagnostic test accuracy of a 2-transcript host RNA signature for discriminating bacterial vs viral infection in febrile children. JAMA. 2016; 316(8):835–45.
28 Lally PJ, Pauliah S, Montaldo P, Chaban B, Oliveira V, Bainbridge A, Soe A, Pattnayak S, Clarke P, Satodia P, Harigopal S, Abernethy LJ, Turner MA, Huertas-Ceballos A, Shankaran S, Thayyil S. Magnetic Resonance Biomarkers in Neonatal Encephalopathy (MARBLE): a prospective multicountry study. BMJ Open.2015;5(9):e008912.

Submit your next manuscript to BioMed Central and we will help you at every step:

- We accept pre-submission inquiries
- Our selector tool helps you to find the most relevant journal
- We provide round the clock customer support
- Convenient online submission
- Thorough peer review
- Inclusion in PubMed and all major indexing services
- Maximum visibility for your research

Submit your manuscript at
www.biomedcentral.com/submit

BioMed Central

Appendix 1: Protocol revision chronology

20 April 2015	Original ethics approval (protocol Version 1.1)
14 July 2015	Amendment 1: (Protocol version 1.2)*<br><br>1) Clarification of the statistical analysis plan in the protocol as suggested by the Independent Data Safety Monitoring Committee<br>2) Making the role of funders explicit in the protocol<br>3) Including publication plans in the protocol
24.6.2016	Amendment 2: (Protocol version 3)<br><br>1) Use of telephone consent if parents are not present at the time of admission to the neonatal unit<br>2) Inclusion of centres in Bangladesh and Sri Lanka<br>3) Sending first birthday cards to recruited babies
12.8.2017	Amendment 3 (Protocol version 3.1)<br><br>1) Discrepancy in the definition of moderate and severe disability noted in various parts of the protocol due to typographic errors.

*Amendment 1 was obtained prior to recruitment of the first case (16 August 2015), and was made to ensure the protocol is complaint with the SPIRIT guidelines

Statsconsultancy Ltd

Address: 40 Longwood Lane, Amersham, Bucks. HP7 9EN
Email: paul@statsconsultancy.co.uk
Phone: 07905 530446
Web: www.statsconsultancy.co.uk

Statistical Analysis Plan

TRIAL FULL TITLE	Hypothermia for Encephalopathy in Low and Middle-Income Countries (HELIX) trial
SAP VERSION	1.2
SAP VERSION DATE	6th July 2017
TRIAL STATISTICIAN	Paul Bassett
SAP AUTHOR	Paul Bassett

9 Table of Contents

1 Table of Contents 64
2 Abbreviations and Definitions 64
3 Introduction 65
4 Study Objectives and Endpoints 65
4.1 Study Objectives 65
4.2 Endpoints 65
4.2.1 Primary outcome measure 65
4.2.2 Secondary outcome measures 66
5 Study Methods 67
5.1 General Study Design and Plan 67
5.2 Inclusion-Exclusion Criteria 67
5.3 Randomisation and Blinding 67
5.4 Demographic and Baseline Variables 68
5.5 Safety measurements 68
6 Sample Size 69
7 General Considerations 69
7.1 Timing of Analyses 69
7.2 Analysis Populations 69
7.3 Subgroups 69
7.4 Missing Data 69
7.5 Multi-centre Studies 69
8 Summary of Study Data 70
8.1 Subject Disposition 70
Follow-Up 71
Analysis 71
Enrolment 71
Allocation 71
8.2 Descriptive Analysis Methods 72
8.3 Demographic and Baseline Variables 72
9 Efficacy Analyses 72
9.1 Primary Efficacy Analysis 72
9.2 Secondary Efficacy Analyses 72
9.3 Exploratory Efficacy Analyses 73
10 Safety Analyses 73
11 Technical Details 73
12 Future changes to the Analysis Plan 73

10 Abbreviations and Definitions

AE	Adverse Event
CI	Confidence Interval
CRF	Case Report Form
HELIX	Hypothermia for Encephalopathy in Low and Middle-Income Countries
IMP	Investigational Medical Product
ITT	Intention to Treat
LMIC	Low and middle-income countries
RR	Risk Ratio
PP	Per Protocol
SAP	Statistical Analysis Plan

11 Introduction

Every year, approximately one million babies die from ‘neonatal encephalopathy’ in low and middle-income countries (LMIC) – a condition arising from unexpected lack of cerebral blood flow and oxygen supply to the fetal brain at the time of birth– a quarter of these deaths occur in India. Approximately a third of infants with moderate or severe encephalopathy will die during the newborn period, and up to three quarters of survivors develop long-term neurodisability. Until recently there was no effective treatment for this condition, and the management was limited to supportive care.

A number of high quality cooling trials have been conducted in high-income countries in the past decade The meta-analyses of these trials have convincingly demonstrated that selective head or whole body cooling along with optimal tertiary intensive care reduce mortality and improve survival with normal neurological outcome after neonatal encephalopathy in these settings.

Although the burden of neonatal encephalopathy is far higher in low and middle-income countries, the safety and efficacy data on cooling therapy from high income cooling trials cannot be extrapolated to these settings. The HELIX trial will examine the safety and efficacy of cooling therapy in under resourced public sector neonatal units in India, who do not have the above-mentioned facilities for providing optimal tertiary intensive care, alongside cooling therapy.

12 Study Objectives and Endpoints

12.1 Study Objectives

The study will assess the following research questions:

Primary objective:

- To examine whether whole body cooling to 33.50C initiated within 6 hours of birth and continued for 72 hours reduces death or neurodisability at 18 months after neonatal encephalopathy in low and middle-income countries.

Secondary objectives:

- To examine if whole body cooling reduces neonatal mortality (30 days) and mortality at 18 to 22 months after neonatal encephalopathy.
- To examine if whole body cooling reduces moderate or severe neurodisability at 18 to 22 months in survivors after neonatal encephalopathy.

12.2 Endpoints

12.2.1 Primary outcome measure

The primary study outcome is death or moderate or severe neurodisability at 18 to 22 months.

Moderate Disability

- Bayley scales of infant development (Version III) composite cognitive or motor score <1SD (70 to 85)
- Gross motor function classification system (GMFCS) level I and 2
- Impaired vision despite correction
- Active seizures
- Hearing impairment requiring amplification to understand commands

Severe Disability is defined as any of the following:
- Bayley scales of infant development (Version III) composite cognitive or motor score <2SD (<70)
- Gross motor function classification system (GMFCS) level 3 to 5
- Impaired vision despite correction
- Hearing impairment despite amplification

12.2.2 Secondary outcome measures

Short term (before discharge from hospital):
- Mortality from any cause
- Major intracranial haemorrhage (evidence of parenchymal bleed on cranial ultrasound)
- Gastric bleeds (fresh blood > 5 ml from nasogastric tube, mouth or rectum)
- Persistent hypotension (mean blood pressure < 25 mm of Hg despite maximal inotropic support)
- Pulmonary haemorrhage (Copious bloody secretions with clinical deterioration requiring change(s) in ventilatory management)
- Persistent pulmonary hypertension (Severe hypoxemia disproportionate to the severity of lung disease with a significant pre-and post ductal saturation difference on pulse oximetry)
- Prolonged blood coagulation time requiring blood products.
- Culture proven early onset sepsis (isolation of a pathogenic organism from blood or cerebrospinal fluid along with clinical evidence of sepsis and elevation of C-reactive protein)
- Necrotising enterocolitis (defined as abdominal distension, increased gastric aspirates and/or blood in stools together with abdominal X-ray showing bowel oedema, pneumatosis or pneumoperitoneum, i.e. Bell's staging 2 or 3)
- Cardiac arrhythmia (ECG trace suggestive of cardiac arrhythmia (other than bradycardia), disregard duration)
- Severe thrombocytopenia (Platelet count <25 without active bleeding or <50 with active bleeding
- Persistent metabolic acidosis (Blood pH < 7.15 for more than 12 hours, with normal PCO2)
- Renal failure (Anuria lasting more than 48 hours with elevated creatinine)
- Pneumonia (Clinical signs of respiratory distress (tachypnea, intercostal retractions and grunting, need for oxygen supplementation, and/or respiratory supports) and typical chest X-ray findings in the presence of probable sepsis and positive tracheal aspirate culture)
- Subcutaneous fat necrosis (indurated, erythematous nodules and plaques over bony prominences such as the back, arms, buttocks, thighs, or cheeks)
- Neurological examination at discharge.
- Duration of hospitalisation

Long term (18 to 22 months):
- Mortality

- Severe neurodevelopmental disability (any of: (i) Bayley III composite cognitive and motor score <2SD (ii) GMFCS levels III,IV,V; (iii) impaired sensory/communication outcomes: blindness, deafness
- Microcephaly (head circumference more than 2 standard deviations below the mean)

13 Study Methods

13.1 General Study Design and Plan

The study is a two arm parallel group randomised control trial. Patients will be randomised to receive either whole body cooling or standard care. The treatment allocation will be unblinded.

The treatment duration (cooling therapy) is 72 hours, however the temperature of all recruited babies will be monitored during the first week after birth. Any temperature rise over >37.50C will be active treated, both in the cooling and usual care arms, as fever increases the brain injury and adverse outcomes after neonatal encephalopathy.

The neurological outcomes will be assessed between 18 to 22 months of age. The trial duration will be 4 years, consisting of a 4 week start up period, 24 month recruitment period, a 18 month follow-up period, and 5 months for data analysis and write up.

13.2 Inclusion-Exclusion Criteria

<u>Inclusion criteria</u>

All included patients will meet the following three criteria:

1. Age < 6 hours, Birth-weight >1.8kg, Gestation >36 weeks based on available information regarding last menstrual period or ultrasound)
2. Need for continued resuscitation at 5 minutes of age and/or 5 minute Apgar score <6 (for babies born at hospital) or lack of cry by 5 minutes of age (for babies born at home)
3. Evidence of moderate or severe encephalopathy at < 6 hours of age on a structured clinical examination

<u>Exclusion criteria</u>

Patients meeting any of the following will be excluded:

- Absent heart rate at 10 minute of age despite adequate resuscitation.
- Major life threatening congenital malformation.
- Migrant family or parents unable/unlikely to come back for follow up at 18 month.
- Lack of parental consent

13.3 Randomisation and Blinding

Patients will be randomised to ether “usual care with cooling” or “usual care only”, on a 1:1 basis. Minimisation will be used to ensure balance between the two study arms throughout the study. The minimisation factors used were the severity of encephalopathy and centre.

Randomisation will be performed, after obtaining parental consent, using a web based database with a central telephone randomisation back up (Sealed envelope; https://www.sealedenvelope.com).

The intervention (cooling therapy) will not be blinded, with no allocation concealment. However, the neurological outcome evaluation at 18 months will be undertaken by assessors masked to the treatment allocation.

13.4 Demographic and Baseline Variables

The following demographic and baseline characteristics of the study participants will be collected:

- Maternal (antenatal) and delivery details including resuscitation details
- Birth weight, gestation and gender
- Hourly rectal temperature profile in all infants for the first 90 hours.
- NICHD neurological examination within 6 hours and at the time of discharge
- Full blood count (including platelets, CRP and differential white cell count) within six hours after birth, and between day 4 and day 7.
- Blood culture (0.5 ml) within 6 hours of birth, and between day 4 and 7.
- Biochemical series (including blood gas, sugar, urea, creatinine, electrolytes, and coagulation profile).
- Cranial US examination (within 72 hours) to examine for major intracranial bleeds.

13.5 Safety measurements

Safety measurements will consist of the measurement of adverse events. Data on all adverse events experienced will be recorded.

Aside from the outcome measures (section 4.2), a number of other adverse event outcomes are also considered. These are divided into two categories:

Adverse events potentially due to cooling therapy:
- Thrombocytopenia and increased need for platelet transfusions
- Subcutaneous fat necrosis

Adverse events that may be due to hypothermia are:
- Cardiac arrhythmia.
- Life threatening bleeds.
- Major venous thrombosis not related to an infusion line.

All adverse events are expected to occur within the cooling period (first 72 hours) or within 72 hours of re-warming. Adverse reactions occurring subsequently (after 1 week of life), except sub cutaneous fat necrosis, will not be considered as intervention related. Sub cutaneous fat necrosis may occur several weeks after the therapy.

14 Sample Size

The sample size is based on being able to detect a clinically significant 30% relative risk reduction in death or moderate/severe disability from 50% in the usual care arm to 35% in the intervention (cooled) arm.

Using a two-sided 5% significance level and an 80% power, it is calculated 183 babies per arm are required. Assuming a loss to follow-up rate of around 10%, this comparison requires 204 babies per group, 408 babies in total, to be recruited.

If in case, the adverse outcomes (death and moderate/severe disability) are higher (~65%) in the usual care arm, then this sample size would provide 94% power to detect a 30% relative risk reduction with cooling.

15 General Considerations

15.1 Timing of Analyses

A single analysis will take place at the completion of the study, after all data is collected. No interim analyses will be performed.

15.2 Analysis Populations

The primary analysis dataset will analyse patients in the groups to which they were randomised, regardless of deviation from the protocol or whether they received the allocated intervention. In other words, analysing on an intention-to-treat (ITT) basis. An additional Per Protocol (PP) dataset will contain only patients who were treated as per the randomisation procedure.

15.3 Subgroups

It proposed that the analysis will be performed for all patients combined, with no subgroup analyses performed.

15.4 Missing Data

Only observed data will be analysed. Missing data will be assumed to be Missing At Random. No imputation procedures will be employed to deal with missing data.

15.5 Multi-centre Studies

The study will recruit patients from up to 7 different centres. The data from all centres will be combined together for the purposes of analysis.

16 Summary of Study Data

16.1 Subject Disposition

A summary of the number of subjects that reached the various stages of the study will be summarised. Reasons for non-participation and withdrawal will be summarised.

A CONSORT diagram will be produced, such as Figure 1, which will illustrate the flow of patients throughout the study.

Figure 1: Outline CONSORT diagram:

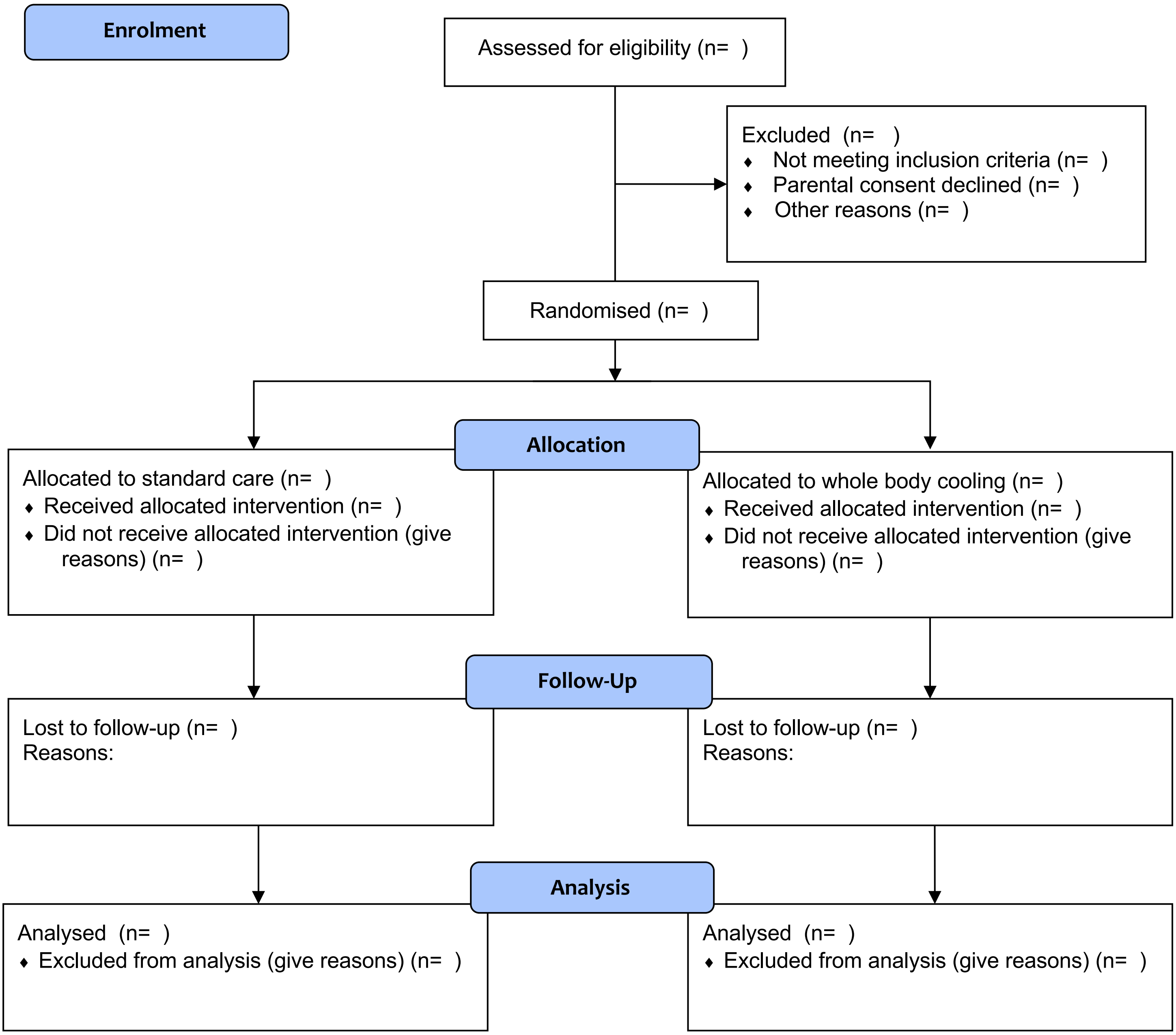

16.2 Descriptive Analysis Methods

Continuous variables will be summarised using the number of (non-missing) datapoints, mean and standard deviation if found to follow a normal distribution. Continuous variables not found to be normally distributed will be summarised by the number of datapoints, median and inter-quartile range. Categorical variables will be summarised by the frequency and percentage (based on the non-missing sample size) of values in each category.

16.3 Demographic and Baseline Variables

A summary of the demographic and baseline variables is given in section 5.4. Each of these measures will be summarised descriptively for the two study arms separately as described. No formal hypothesis tests will be used to compare the two groups for these measures

A single set of summaries will be produced for all centres combined.

17 Efficacy Analyses

17.1 Primary Efficacy Analysis

The primary study outcome is death or moderate or severe neurodisability at 18 to 22 months (as defined more fully in Section 4.2.1). The summary statistics for each trial arm will be produced in accordance with section 16.

The difference in outcome being study arms will be summarised by calculating the risk ratio (RR) for the occurrence of the outcome in the whole body cooling group relative to the occurrence of the outcome in the standard care arm. A corresponding 95% confidence interval (CI) for the RR will be calculated. Additionally the chi-square test will be used to determine statistical significance, with a 5% significance level used.

The primary analysis will be performed using the ITT dataset, whilst a sensitivity analysis will use the PP dataset.

17.2 Secondary Efficacy Analyses

Secondary outcomes will be evaluated using a 5% level of statistical significance, with 95% CIs reported, in order to take account of the number of outcomes analysed.

Categorical secondary outcomes will be compared between study arms using the Chi-square test or Fisher's exact test, depending on the frequency of the outcome. Differences in binary outcomes between arms will be reported as risk ratios, along with corresponding reported with 95% CIs.

The unpaired t-test will be used to analyse normally distributed continuous outcomes, with the mean difference (plus 95% CI) reported. Non-normally distributed continuous outcomes will be transformed to normality and analysed using the unpaired paired t-test as per the normally distributed outcomes. Alternatively non-normally distributed analysed may be using the Mann-Whitney test. If the latter approach is used, the median difference (plus 95% CI) between groups will be reported.

17.3 Exploratory Efficacy Analyses

Additional exploratory analyses will be performed to investigate the impact of stratification/known prognostic factors, including the stage of neonatal encephalopathy, on the primary outcome (death or moderate or severe neurodisability at 18 to 22 months). These analyses will be performed using logistic regression.

18 Safety Analyses

The main safety outcome is the occurrence of adverse events. Adverse events are defined in section 5.5. The number of adverse events will be reported descriptively as outlined in section 8.2

Each adverse event, if any, will be judged to be related to the patients' participation in the study (definite, probable, possible, unlikely). Separate summaries will be produced for AEs related and not related to study participation. The expectedness of the AE will also be reported.

Adverse events which do not occur very frequently will be analysed descriptively only. Where it deemed that there are sufficient occurrences, a formal test of significance will be performed. Either the Chi-square test or Fisher's exact test will be used to test the occurrence of each adverse event variable between study groups.

19 Technical Details

The data analysis will be performed using the statistical software package Stata (version 13.1). Programs recording details of all data manipulation and data analyses will be produced and kept, so that the analyses can be externally inspected and, if necessary, re-run.

20 Future changes to the Analysis Plan

The primary revisions since the previous version of the SAP are:

- Secondary analyses performed at a 5% significance level, rather than 1% as previously suggested
- Additional of a Per Protocol analysis for the primary outcome

Statistical Analysis Plan

TRIAL FULL TITLE	Hypothermia for Encephalopathy in Low and Middle-Income Countries (HELIX) trial
SAP VERSION	1.3
SAP VERSION DATE	13th August 2020
TRIAL STATISTICIAN	Paul Bassett
SAP AUTHOR	Paul Bassett

21 Table of Contents

1 Table of Contents 75
2 Abbreviations and Definitions 75
3 Introduction 76
4 Study Objectives and Endpoints 76
4.1 Study Objectives 76
4.2 Endpoints 76
4.2.1 Primary outcome measure 76
4.2.2 Secondary outcome measures 77
5 Study Methods 78
5.1 General Study Design and Plan 78
5.2 Inclusion-Exclusion Criteria 78
5.3 Randomisation and Blinding 78
5.4 Demographic and Baseline Variables 79
5.5 Safety measurements 79
6 Sample Size 80
7 General Considerations 80
7.1 Timing of Analyses 80
7.2 Analysis Populations 80
7.3 Subgroups 80
7.4 Missing Data 80
7.5 Multi-centre Studies 80
8 Summary of Study Data 81
8.1 Subject Disposition 81
Follow-Up 82
Analysis 82
Enrolment 82
Allocation 82
8.2 Descriptive Analysis Methods 83
8.3 Demographic and Baseline Variables 83
9 Efficacy Analyses 83
9.1 Primary Efficacy Analysis 83
9.2 Secondary Efficacy Analyses 83
9.3 Exploratory Efficacy Analyses 84
10 Safety Analyses 84
11 Technical Details 84
12 Changes from previous versions of the Analysis Plan 85
12.1 Changes from Version 1.1 to 1.2 85
12.2 Changes from Version 1.2 to 1.3 85

22 Abbreviations and Definitions

AE	Adverse Event
CI	Confidence Interval
CRF	Case Report Form
HELIX	Hypothermia for Encephalopathy in Low and Middle-Income Countries
IMP	Investigational Medical Product
ITT	Intention to Treat
LMIC	Low and middle-income countries
RR	Risk Ratio
PP	Per Protocol
SAP	Statistical Analysis Plan

23 Introduction

Every year, approximately one million babies die from 'neonatal encephalopathy' in low and middle-income countries (LMIC) – a condition arising from unexpected lack of cerebral blood flow and oxygen supply to the fetal brain at the time of birth– a quarter of these deaths occur in India. Approximately a third of infants with moderate or severe encephalopathy will die during the newborn period, and up to three quarters of survivors develop long-term neurodisability. Until recently there was no effective treatment for this condition, and the management was limited to supportive care.

A number of high-quality cooling trials have been conducted in high-income countries in the past decade. The meta-analyses of these trials have convincingly demonstrated that selective head or whole-body cooling along with optimal tertiary intensive care reduce mortality and improve survival with normal neurological outcome after neonatal encephalopathy in these settings.

Although the burden of neonatal encephalopathy is far higher in low and middle-income countries, the safety and efficacy data on cooling therapy from high income cooling trials cannot be extrapolated to these settings. The HELIX trial will examine the safety and efficacy of cooling therapy in under resourced public sector neonatal units in India, who do not have the above-mentioned facilities for providing optimal tertiary intensive care, alongside cooling therapy.

24 Study Objectives and Endpoints

24.1 Study Objectives

The study will assess the following research questions:

Primary objective:

- To examine whether whole body cooling to 33.50C initiated within 6 hours of birth and continued for 72 hours reduces death or neurodisability at 18 months after neonatal encephalopathy in low and middle-income countries.

Secondary objectives:

- To examine if whole body cooling reduces neonatal mortality (30 days) and mortality at 18 to 22 months after neonatal encephalopathy.
- To examine if whole body cooling reduces moderate or severe neurodisability at 18 to 22 months in survivors after neonatal encephalopathy.

24.2 Endpoints

24.2.1 <u>Primary outcome measure</u>

The primary study outcome is death or moderate or severe neurodisability at 18 to 22 months.

Moderate Disability is defined as:

- Bayley scales of infant development (Version III) composite cognitive score <1SD (70 to 84) AND ANY of the following:
    - o Gross motor function classification system (GMFCS) level 2
    - o Seizure disorder
    - o Hearing impairment requiring amplification to understand commands

Severe Disability is defined as ANY of the following:
- Bayley scales of infant development (Version III) composite cognitive <2SD (<70)
- Gross motor function classification system (GMFCS) level 3 to 5
- Blindness
- Hearing impairment despite amplification

24.2.2 Secondary outcome measures

Short term (before discharge from hospital):
- Mortality from any cause
- Major intracranial haemorrhage (evidence of parenchymal bleed on cranial ultrasound)
- Gastric bleeds (fresh blood > 5 ml from nasogastric tube, mouth or rectum)
- Persistent hypotension (mean blood pressure < 25 mm of Hg despite maximal inotropic support)
- Pulmonary haemorrhage (Copious bloody secretions with clinical deterioration requiring change(s) in ventilatory management)
- Persistent pulmonary hypertension (Severe hypoxemia disproportionate to the severity of lung disease with a significant pre-and post-ductal saturation difference on pulse oximetry)
- Prolonged blood coagulation time requiring blood products.
- Culture proven early onset sepsis (isolation of a pathogenic organism from blood or cerebrospinal fluid along with clinical evidence of sepsis and elevation of C-reactive protein)
- Necrotising enterocolitis (defined as abdominal distension, increased gastric aspirates and/or blood in stools together with abdominal X-ray showing bowel oedema, pneumatosis or pneumoperitoneum, i.e. Bell's staging 2 or 3)
- Cardiac arrhythmia (ECG trace suggestive of cardiac arrhythmia (other than bradycardia), disregard duration)
- Severe thrombocytopenia (Platelet count <25 without active bleeding or <50 with active bleeding
- Persistent metabolic acidosis (Blood pH < 7.15 for more than 12 hours, with normal PCO2)
- Renal failure (Anuria lasting more than 48 hours with elevated creatinine)
- Pneumonia (Clinical signs of respiratory distress (tachypnea, intercostal retractions and grunting, need for oxygen supplementation, and/or respiratory supports) and typical chest X-ray findings in the presence of probable sepsis and positive tracheal aspirate culture)
- Subcutaneous fat necrosis (indurated, erythematous nodules and plaques over bony prominences such as the back, arms, buttocks, thighs, or cheeks)
- Neurological examination at discharge.
- Duration of hospitalisation

Long term (18 to 22 months):
- Mortality

- Severe neurodevelopmental disability (any of: (i) Bayley III composite cognitive score <2SD (ii) GMFCS levels III, IV, V (iii) impaired sensory/communication outcomes: blindness, deafness
- Microcephaly (head circumference more than 2 standard deviations below the mean)

25 Study Methods

25.1 General Study Design and Plan

The study is a two-arm parallel group randomised control trial. Patients will be randomised to receive either whole body cooling or standard care. The treatment allocation will be unblinded.

The treatment duration (cooling therapy) is 72 hours, however the temperature of all recruited babies will be monitored during the first week after birth. Any temperature rise over >37.50C will be active treated, both in the cooling and usual care arms, as fever increases the brain injury and adverse outcomes after neonatal encephalopathy.

The neurological outcomes will be assessed between 18 to 22 months of age. The trial duration will be 4 years, consisting of a 4 week start up period, 24-month recruitment period, an 18-month follow-up period, and 5 months for data analysis and write up.

25.2 Inclusion-Exclusion Criteria

<u>Inclusion criteria</u>

All included patients will meet the following three criteria:

4. Age < 6 hours, Birth-weight >1.8kg, Gestation >36 weeks based on available information regarding last menstrual period or ultrasound)
5. Need for continued resuscitation at 5 minutes of age and/or 5-minute Apgar score <6 (for babies born at hospital) or lack of cry by 5 minutes of age (for babies born at home)
6. Evidence of moderate or severe encephalopathy at < 6 hours of age on a structured clinical examination

<u>Exclusion criteria</u>

Patients meeting any of the following will be excluded:

- Absent heart rate at 10 minutes of age despite adequate resuscitation.
- Major life-threatening congenital malformation.
- Migrant family or parents unable/unlikely to come back for follow up at 18 months
- Lack of parental consent

25.3 Randomisation and Blinding

Patients will be randomised to ether “usual care with cooling” or “usual care only”, on a 1:1 basis. Minimisation will be used to ensure balance between the two study arms throughout the study. The minimisation factors used were the severity of encephalopathy and centre.

Randomisation will be performed, after obtaining parental consent, using a web-based database with a central telephone randomisation back up (Sealed envelope; https://www.sealedenvelope.com).

The intervention (cooling therapy) will not be blinded, with no allocation concealment. However, the neurological outcome evaluation at 18 months will be undertaken by assessors masked to the treatment allocation.

25.4 Demographic and Baseline Variables

The following demographic and baseline characteristics of the study participants will be collected:

- Maternal (antenatal) and delivery details including resuscitation details
- Birth weight, gestation and gender
- Hourly rectal temperature profile in all infants for the first 90 hours.
- NICHD neurological examination within 6 hours and at the time of discharge
- Full blood count (including platelets, CRP and differential white cell count) within six hours after birth, and between day 4 and day 7.
- Blood culture (0.5 ml) within 6 hours of birth, and between day 4 and 7.
- Biochemical series (including blood gas, sugar, urea, creatinine, electrolytes, and coagulation profile).
- Cranial US examination (within 72 hours) to examine for major intracranial bleeds.

25.5 Safety measurements

Safety measurements will consist of the measurement of adverse events. Data on all adverse events experienced will be recorded.

Aside from the outcome measures (section 4.2), a number of other adverse event outcomes are also considered. These are divided into two categories:

Adverse events potentially due to cooling therapy:
- Thrombocytopenia and increased need for platelet transfusions
- Subcutaneous fat necrosis

Adverse events that may be due to hypothermia are:
- Cardiac arrhythmia.
- Life threatening bleeds.
- Major venous thrombosis not related to an infusion line.

All adverse events are expected to occur within the cooling period (first 72 hours) or within 72 hours of re-warming. Adverse reactions occurring subsequently (after 1 week of life), except sub cutaneous fat necrosis, will not be considered as intervention related. Sub cutaneous fat necrosis may occur several weeks after the therapy.

26 Sample Size

The sample size is based on being able to detect a clinically significant 30% relative risk reduction in death or moderate/severe disability from 50% in the usual care arm to 35% in the intervention (cooled) arm.

Using a two-sided 5% significance level and an 80% power, it is calculated 183 babies per arm are required. Assuming a loss to follow-up rate of around 10%, this comparison requires 204 babies per group, 408 babies in total, to be recruited.

If in case, the adverse outcomes (death and moderate/severe disability) are higher (~65%) in the usual care arm, then this sample size would provide 94% power to detect a 30% relative risk reduction with cooling.

27 General Considerations

27.1 Timing of Analyses

A single analysis will take place at the completion of the study, after all data is collected. No interim analyses will be performed.

27.2 Analysis Populations

The primary analysis will analyse patients in the groups to which they were randomised, regardless of deviation from the protocol or whether they received the allocated intervention. In other words, analysing on an intention-to-treat (ITT) basis.

An additional Per Protocol (PP) dataset will contain only patients who were treated as per the randomisation procedure.

27.3 Subgroups

It proposed that the analysis will be performed for all patients combined, with no subgroup analyses performed.

27.4 Missing Data

Only observed data will be analysed. Missing data will be assumed to be Missing At Random. No imputation procedures will be employed to deal with missing data.

27.5 Multi-centre Studies

The study will recruit patients from up to 7 different centres. The data from all centres will be combined together for the purposes of analysis.

28 Summary of Study Data

28.1 Subject Disposition

A summary of the number of subjects that reached the various stages of the study will be summarised. Reasons for non-participation and withdrawal will be summarised.

A CONSORT diagram will be produced, such as Figure 1, which will illustrate the flow of patients throughout the study.

Figure 1: Outline CONSORT diagram:

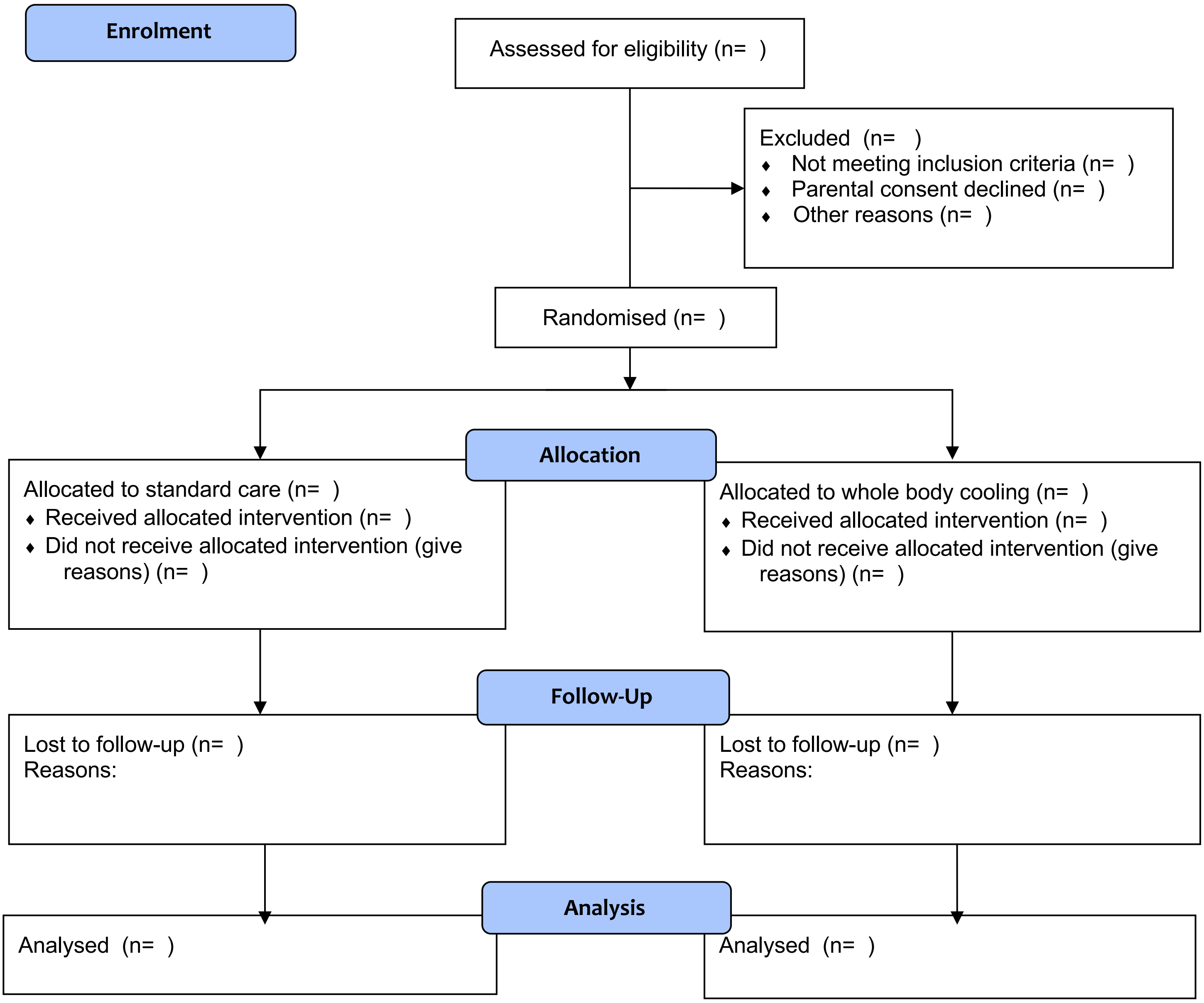

28.2 Descriptive Analysis Methods

Continuous variables will be summarised using the number of (non-missing) datapoints, mean and standard deviation if found to follow a normal distribution. Continuous variables not found to be normally distributed will be summarised by the number of datapoints, median and inter-quartile range. Categorical variables will be summarised by the frequency and percentage (based on the non-missing sample size) of values in each category.

28.3 Demographic and Baseline Variables

A summary of the demographic and baseline variables is given in section 5.4. Each of these measures will be summarised descriptively for the two study arms separately as described. No formal hypothesis tests will be used to compare the two groups for these measures

A single set of summaries will be produced for all centres combined.

29 Efficacy Analyses

29.1 Primary Efficacy Analysis

The primary study outcome is death or moderate or severe neurodisability at 18 to 22 months (as defined more fully in Section 4.2.1). The summary statistics for each trial arm will be produced in accordance with section 8.

The difference in outcome being study arms will be summarised by calculating the risk ratio (RR) for the occurrence of the outcome in the whole-body cooling group relative to the occurrence of the outcome in the standard care arm. A corresponding 95% confidence interval (CI) for the RR will be calculated. Additionally, the chi-square test will be used to determine statistical significance, with a two-sided 5% significance level used.

The primary analysis will be performed using the ITT dataset, whilst an additional analysis will use the PP dataset.

A further sensitivity analysis will be performed, in which the treatment differences will be evaluated after adjusting for both centre and the level of encephalopathy. This analysis will be performed using logistic regression.

29.2 Secondary Efficacy Analyses

Categorical secondary outcomes will be compared between study arms using the Chi-square test or Fisher's exact test, depending on the frequency of the outcome. Differences in binary outcomes between arms will be reported as risk ratios, along with corresponding reported with 95% CIs.

The unpaired t-test will be used to analyse normally distributed continuous outcomes, with the mean difference (plus 95% CI) reported. Non-normally distributed continuous outcomes will be transformed to normality and analysed using the unpaired paired t-test as per the normally distributed outcomes. Alternatively, non-normally distributed analysed may be using the Mann-Whitney test. If the latter approach is used, the median difference (plus 95% CI) between groups will be reported.

All secondary outcomes will be evaluated using a 5% level of statistical significance.

29.3 Exploratory Efficacy Analyses

Additional exploratory analyses will be performed to investigate the impact of stratification/known prognostic factors, including the stage of neonatal encephalopathy, on the primary outcome (death or moderate or severe neurodisability at 18 to 22 months). These analyses will be performed using logistic regression.

30 Safety Analyses

The main safety outcome is the occurrence of adverse events. Adverse events are defined in section 5.5. The number of adverse events will be reported descriptively as outlined in section 8.2

Each adverse event, if any, will be judged to be related to the patients' participation in the study (definite, probable, possible, unlikely). Separate summaries will be produced for AEs related and not related to study participation. The expectedness of the AE will also be reported.

Adverse events which do not occur very frequently will be analysed descriptively only. Where it deemed that there are sufficient occurrences, a formal test of significance will be performed. Either the Chi-square test or Fisher's exact test will be used to test the occurrence of each adverse event variable between study groups.

31 Technical Details

The data analysis will be performed using the statistical software package Stata (version 15.1). Programs recording details of all data manipulation and data analyses will be produced and kept, so that the analyses can be externally inspected and, if necessary, re-run.

32 Changes from previous versions of the Analysis Plan

32.1 Changes from Version 1.1 to 1.2

The primary revisions between versions of the SAP were:

- Secondary analyses performed at a 5% significance level, rather than 1% as previously suggested
- Addition of a Per Protocol analysis for the primary outcome

32.2 Changes from Version 1.2 to 1.3

Changes between these versions were mostly cosmetic, providing some further information on the study outcomes

HELIX Trial:

Hypothermia for Encephalopathy in Low and Middle-Income Countries (HELIX) trial

A phase III, un-blinded pragmatic randomised controlled trial of whole body cooling versus usual care, in babies with neonatal encephalopathy, admitted to low resourced neonatal units in India.

Clinical trials Number: NCT02387385

Independent Data Monitoring Committee Charter

Version 1.0, 12 April 2015

(developed using on MRC Clinical Trials Unit template IDMC Charter version 2.01, 13-Mar-2006; from DAMOCLES IDMC Charter template v1, Feb 2005)

Authorised by:

Name:	Dr Sudhin Thayyil	Role:	Chief Investigator
Signature:		Date:	12 April 2015

32.3 IDMC CHARTER FOR HELIX TRIAL

**32.4 CONTENT**<br>32.5 Guidance	**32.6 CHARTER DETAILS**
32.6.11. INTRODUCTION	32.6.2
Name of trial	A phase III, un-blinded pragmatic randomised controlled trial of whole body cooling versus usual care, in babies with neonatal encephalopathy, admitted to low resourced neonatal units in India.<br><br>HELIX Trial – Hypothermia for Encephalopathy in Low and Middle-Income Countries trial
Objectives of trial, including interventions being investigated	To examine whether whole body cooling to 33.5^{0}C initiated within 6 hours of birth and continued for 72 hours reduces death or neurodisability at 18 months after neonatal encephalopathy in low and middle-income countries.<br>A study summary diagram is included in Figure 1.
Outline of scope of charter	The purpose of this document is to describe the membership, terms of reference, roles, responsibilities, authority, decision-making and relationships of the independent IDMC for the this trial, including the timing of meetings, methods of providing information to and from the IDMC, frequency and format of meetings and statistical issues.
32.6.32. ROLES AND RESPONSIBILITIES	32.6.4
A broad statement of the aims of the committee	To safeguard the interests of trial participants, monitor the main outcome measures including safety and efficacy, and monitor the overall conduct of the trial.
Terms of reference	***32.6.4.1.1 The IDMC should receive and review information on the progress and accruing data of this trial and provide advice on the conduct of the trial to the Trial Steering Committee (TSC).***<br>The IDMC should inform the PI and Chair of the TSC if, in their view the results are likely to convince a broad range of clinicians, including those supporting the trial and the general clinical community, that, on balance, one trial arm is clearly indicated or contraindicated for all participants or a particular category of participants, and there was a reasonable expectation that this new evidence would materially influence patient management.
Specific roles of IDMC	Interim review of the trial's progress including updated figures on recruitment, data quality, adherence to protocol treatment and follow-up, and main outcomes and safety data. Specifically, these roles include to:<br>• monitor evidence for treatment differences in the main efficacy outcome measures<br>• monitor evidence for treatment harm (e.g. SAEs and SARs, deaths)<br>• assess the impact and relevance of external evidence<br>• decide whether to recommend that the trial continues to recruit participants or whether recruitment should be terminated either for everyone or for some treatment groups and/or some participant subgroups<br>• decide whether trial follow-up should be stopped earlier

32.4 CONTENT<br>32.5 Guidance	32.6 CHARTER DETAILS
	• assess data quality, including completeness (and by so doing encourage collection of high quality data)<br>• maintain confidentiality of all trial information that is not in the public domain<br>• monitor recruitment figures and losses to follow-up<br>• monitor compliance with the protocol by participants and investigators<br>• consider the ethical implications of any recommendations made by the IDMC<br>• monitor planned sample size assumptions, preferably with regards to (i) a priori assumptions about the control arm outcome and/or (ii) emerging differences in clinically relevant subgroups, rather than on emerging, unblinded differences between treatment groups, overall<br>• suggest additional data analyses if necessary<br>• advise on protocol modifications proposed by investigators or sponsors (e.g. to inclusion criteria, trial endpoints, or sample size)<br>• monitor compliance with previous IDMC recommendations
32.6.53. BEFORE OR EARLY IN THE TRIAL	32.6.6
Whether the IDMC will have input into the protocol	All potential IDMC members should have sight of the protocol before agreeing to join the committee. Before recruitment begins the trial will have undergone review by the funder/sponsor (e.g. peer review for public sector trials), scrutiny by other trial committees and a research ethics committee (REC). Therefore, if a potential IDMC member has reservations about the trial (e.g. the protocol or the logistics) they should report these to the CI and may decide not to accept the invitation to join or the invitation will be withdrawn by the trial team. IDMC members should be independent1 and constructively critical of the ongoing trial, but also supportive of aims and methods of the trial.
Whether the IDMC will meet before the start of the trial	The first meeting will be convened at the earliest opportunity, to discuss the protocol, the trial, the analysis plan, future meetings, and to have the opportunity to clarify any aspects with the Chief Investigator (CI) and Trial Management Group (TMG). The IDMC should meet within one year of recruitment commencing.
Whether members of the IDMC will have a contract	IDMC members will not formally sign a contract but should formally register their assent to join the group by confirming (1) that they agree to be on the IDMC and (2) that they agree with the contents of this Charter. Any competing interests should be declared at the same time. Members should complete and return the form in Annexe 1.
32.6.74. COMPOSITION	32.6.8
Membership and size of the IDMC	The members of the IDMC for this trial are:<br>(1) Prof Abbot Laptook – Chair/Neonatologist<br>(2) Prof Shabbar Jaffar – Epidemiologist/trialist<br>(3) Prof Niranjan Thomas – Neonatologist

[1] Independence is defined in the table in Annexe 1

32.4 CONTENT<br>32.5 Guidance	32.6 CHARTER DETAILS
	The members should be independent of the trial (should not be involved with the trial in any other way or have any competing interest(s) that could impact on the trial). Any competing interests, both real and potential, should be declared. A short competing interest form should be completed and returned by the IDMC members to the trial coordinating centre (Annexe 1).
The Chair, how they are chosen and the Chair's role. (Likewise, if relevant, the vice-Chairman)	The Chair will have previous experience of serving on IDMCs, experience of Chairing meetings and will be able to facilitate and summarise discussions. The Chair will be nominated and agreed by the TMG. The Chair is expected to facilitate and summarise discussions.
The responsibilities of the IDMC statistician/s	The IDMC membership will include an experienced clinical trialist to provide independent statistical expertise, especially with regards to interpretation of accumulating data and guidance through the report.
The responsibilities of the IDMC Clinician/s	To advise on safety and wellbeing of trial participants, and severity and significance of AEs and SAEs
The responsibilities of the trial statistician	The trial statistician will have overall responsibility for the production of the report to the IDMC and will participate in IDMC meetings, guiding the IDMC through the report, participating in IDMC discussions.
The responsibilities of the trial manager	The trial team will help the trial statistician to produce the report to the IDMC. The Trial Manager may attend open sessions of the meeting.
The responsibilities of the Chief Investigator and other members of the Trial Management Group (TMG)	The CI should be invited and should be available, to attend open sessions of the IDMC meeting. The other TMG members will not usually be expected to attend but can attend open sessions when necessary.
32.6.95. RELATIONSHIPS	32.6.10
Relationships with Chief Investigators, other trial committees (e.g. Trial Steering Committee (TSC) or Executive Committee), sponsor and regulatory bodies	The responsibilities of each trial group are detailed in the protocol.
Clarification of whether the IDMC is advisory (make recommendations) or executive (make decisions)	The IDMC is advisory to the CI and the TSC.
Payments to IDMC members	Members will be reimbursed for reasonable travel costs and accommodation where required. No other payments or rewards are given.
The need for IDMC members to disclose information about any competing interests	Competing interests should be disclosed. These are not restricted to financial matters – involvement in other trials or intellectual investment could be relevant. Although members may well be able to act objectively despite such connections, complete disclosure enhances credibility. (See Annexe 1)<br>IDMC members should not use interim results to inform trading in pharmaceutical shares, and careful consideration should be given to trading in stock of companies with competing products.

32.4 CONTENT<br>32.5 Guidance	32.6 CHARTER DETAILS
32.6.11 6. ORGANISATION OF MEETINGS	32.6.12
Expected frequency of IDMC meetings	A quorate meeting will include at least three IDMC members including the chairperson. The first meeting will be convened at the earliest opportunity. Subsequent reviews will be held 2-3 times a year or more frequently as requested by the IDMC.<br><br>In exceptional circumstances it may be necessary to convene an unscheduled meeting of the IDMC. This could be if there were concerns about the frequency or severity of adverse events being reported
Whether meetings will be face-to-face or by teleconference	The IDMC meetings may be face-to-face or by teleconference calls. Parts of the meeting may be open at the discretion of the IDMC. The first meeting will be an open session.
How IDMC meetings will be organised, especially regarding open and closed sessions, including who will be present in each session	A mixture of open and closed sessions will be held. Only IDMC members and others whom they specifically invite, e.g. the trial statistician/senior scientist are present in closed sessions. In open sessions, all those attending the closed session may be joined by the CI(s), other members of the trials unit team and sometimes also representatives of the sponsor, funder, or regulator, as relevant.<br><br>The format of the meetings will be based on the following structure:<br><br>1. Open session: Introduction and any "open" parts of the report<br><br>2. Closed session: IDMC discussion of "closed" parts of the report and, if necessary, the trial statistician will attend only part of these discussions<br><br>3. Open session: Discussion with other attendees on any matters arising from the closed session.<br><br>4. Closed session: extra closed session (if necessary)
32.6.13 7. TRIAL DOCUMENTATION AND PROCEDURES TO ENSURE CONFIDENTIALITY AND PROPER COMMUNICATION	
Intended content of material to be available in open sessions	Data reviewed will include accrual and retention rates by site, adverse events, withdrawals and therapeutic outcomes. Adverse events based on pooled data will be presented and total numbers of events for the primary outcome measure and other outcome measures may be presented, at the discretion of the IDMC.<br><br>At any time, the IDMC may choose to add to the list of collated data for the reports, but the following should be included on an individual patient listing level:<br><br>Study ID<br>Date of recruitment<br>Trial arm<br>Date of serious adverse event<br>Type of serious adverse event<br>Outcome<br>Date of outcome<br><br>In addition, serious adverse event forms sent to the IDMC chair will include a short clinical description of the event.
Intended content of material to be available in closed sessions	In addition to all the material available in the open session, the closed session material will include efficacy and safety data by treatment group (see next point below about blinding).

**32.4 CONTENT**<br>32.5 Guidance	**32.6 CHARTER DETAILS**
Whether or not the IDMC will be blinded to the treatment allocation	The IDMC will be kept blind to the study arms and unblinded on request. The two study arms will be labelled A and B.
The people who will see the accumulating data and interim analysis	The accumulating data and interim analysis by randomised group (as labelled above) will be seen by the IDMC members and trial statistician.
Responsibility for identifying and circulating external evidence (e.g. from other trials/ systematic reviews)	Identification and circulation of external evidence (e.g. from other trials/ systematic reviews) is not the responsibility of the IDMC members. The CI and the TMG will collate any such information for presentation in an open session.
To whom the IDMC will communicate the decisions/ recommendations that are reached	The IDMC reports its recommendations in writing to the CI. It is the CI's responsibility to forward to the TSC and the trial statistician.<br>If the trial is to continue largely unchanged it is useful for the report from the IDMC to include a summary paragraph suitable for trial promotion purposes ie to be circulated to trial sites.<br>In its communications, the IDMC should be careful not to relay any unnecessary information to the TSC: the TSC membership has independent members but also representatives from the trial team including the CI. The IDMC should take care to protect the CI from interim trial data where possible.
Whether reports to the IDMC be available before the meeting or only at/during the meeting	The IDMC should receive the report at least 1 week and preferably at least 2 weeks before any meetings.
What will happen to the confidential papers after the meeting	The IDMC members should store the papers safely after each meeting so they may check the next report against them. After the trial is reported, the IDMC members should destroy all interim reports. A copy of all of the reports will be archived at Imperial College London at the end of the study. Fresh copies of previous reports may be circulated (by email) with the newest report before each meeting.
32.6.14 8. DECISION MAKING	32.6.15
What decisions/recommendations will be open to the IDMC	Possible recommendations from the IDMC include:<br>• No action needed, i.e. continue the study according to the protocol<br>• Discontinue the study (with provisions for orderly discontinuation in accordance with good medical practice) due, for example, to clear benefit or harm of a treatment or external evidence. (This should generally involve a recommendation to unblind the TSC to this data)<br>• Extending recruitment to increase the sample size (based on actual control arm event rates being lower than predicted rather than on emerging differences)<br>• Proposing or commenting on proposed protocol changes<br>• Commenting on Statistical Analysis Plan
The role of formal statistical methods, specifically which methods will be used and whether they will be used as guidelines or rules	Formal statistical methods are more generally used as "stopping" guidelines rather than absolute rules. This is because they generally only consider one dimension of the trial. Reasons should be recorded for disregarding a stopping guideline. The statistical guidelines for the trial are described in outline in the protocol and in detail in the Statistical Analysis Plan.

32.4 CONTENT<br>32.5 Guidance	32.6 CHARTER DETAILS
How decisions or recommendations will be reached within the IDMC	Interpretation of safety data will require both clinical and statistical experts reviewing the data in concert. A number of considerations for interpretation of these data can be stated and these include:<br><br>1. Whether the results could be explained by possible differences in the baseline variables between the groups;<br>2. Whether the ascertainment of outcomes could be biased;<br>3. Whether the results are consistent across different variables;<br>4. Whether the particular risk is outweighed by assessment of the benefits of the intervention;<br>5. Whether the results could be due to concomitant therapy and not due to the intervention;<br>6. Whether it is likely that the current trends could be reversed if the trial were to be continued unmodified;<br>7. Whether and how much additional precision could be obtained by continuing the trial under the present protocol; and,<br>8. Whether there would be significant loss in the overall assessment of the validity of the trial by the medical community by discontinuation or change in the protocol.<br><br>In making recommendations, the IDMC must take into account other prevailing information relevant to the study, in particular, emerging reliable data from low and middle-income countries.<br><br>The Chair is to summarise discussions and encourage consensus; it is usually best for the Chair to give their own opinion last.<br><br>Every effort should be made for the IDMC to reach a unanimous decision. If the IDMC cannot achieve this, a vote may be taken, although details of the vote should not be routinely included in the report to the TSC as these may inappropriately convey information about the state of the trial data.<br><br>It is important that the implications (e.g. ethical, statistical, practical, financial) for the trial be considered before any recommendation is made.
When the IDMC is quorate for decision-making	Efforts should be made to ensure that all members can attend. The TMG will try to ensure that a date is chosen to enable this. Members who cannot attend in person should be encouraged to attend by teleconference. If, at short notice, any IDMC members cannot attend at all then the IDMC may still meet if at least one statistician and one clinician, including the Chair (unless otherwise agreed), will be present. If the IDMC is considering recommending major action after such a meeting the IDMC Chair should communicate with the absent members as soon after the meeting as possible to check they agree. If they do not, a further meeting should be arranged with the full IDMC.
Can IDMC members who cannot attend the meeting input	If the report is circulated before the meeting, IDMC members who will not be able to attend the meeting may pass comments to the IDMC Chair for consideration during the discussions.
What happens to members who do not attend meetings	If a member does not attend a meeting, it should be ensured that the member is available for the next meeting. If a member does not attend the following meeting, they should be asked if they wish to remain part of the IDMC.
32.6.16 9. <u>REPORTING</u>	32.6.17
To whom will the IDMC report their recommendations/decisions, and in what form	The IDMC may request additional analyses before they make any suggestions that the study be modified. The recommendation of the IDMC following each meeting should be sent to the CI, usually within 2 weeks of the meeting. It is his responsibility to forward the

32.4 CONTENT<br>32.5 Guidance	32.6 CHARTER DETAILS
	recommendation to the chair of the TSC. A copy of this will be stored at Imperial College London. A copy will also be sent to the sponsor.
Whether minutes of the meeting be made and, if so, by whom and where they will be kept	Separate records will be required for open and closed sessions with minutes made by the appropriate attending member of the trial team. This will usually be the Trial Manager for the open session and the Chair or other IDMC member for the closed session. The IDMC Chair should sign off any minutes or notes.
What will be done if there is disagreement between the IDMC and the body to which it reports	If the IDMC has serious problems or concerns with the TSC decision a meeting of these groups should be held. The information to be shown would depend upon the action proposed and the IDMC's concerns. Depending on the reason for the disagreement confidential data would often have to be revealed to all those attending such a meeting. The meeting would be Chaired by an external expert who is not directly involved with the trial.
32.6.18 10. AFTER THE TRIAL	32.6.19
Publication of results	Depending on the results of the trial and the ease of their interpretation, at the end of the trial there may be a meeting to allow the IDMC to discuss the final data with the writing committee to give advice about data interpretation.<br>The main trial results will be published in a correct and timely manner.
The information about the IDMC that will be included in published trial reports	IDMC members will be named and their affiliations listed in the main report, unless they explicitly request otherwise. A brief summary of the timings and conclusions of IDMC meetings should be included in the body of this paper.
Whether the IDMC will have the opportunity to approve publications, especially with respect to reporting of any IDMC recommendation regarding termination of a trial	The IDMC will be given the opportunity to read and comment on publications before submission.
Any constraints on IDMC members divulging information about their deliberations after the trial has been published	12 months after the primary trial results have been published, or when permission is agreed with the overseeing committee.

11. Contact details

Chief Investigator

Sudhin Thayyil
Weston Reader and Honorary Consultant Neonatologist
Director, Centre for Perinatal Neuroscience
Department of Paediatrics
Imperial College London
Du Cane Road
London W12 0HS

IDMC

Professor Abbot Laptook (Chair of IDMC)

32.4 CONTENT<br>32.5 Guidance	32.6 CHARTER DETAILS
Professor for Neonatology<br>Brown University<br>United States of America<br><br>**Professor Shabbar Jaffar**<br>Faculty of Epidemiology and Population Health<br>London School of Hygiene and Tropical Medicine<br>London, United Kingdom<br><br>**Professor Niranjan Thomas**<br>Professor of Neonatology<br>Christian Medical College<br>Vellore, India<br><br>**Dr Aung Soe**<br>Clinical Director and Consultant Neonatologist<br>Medway NHS Hospital<br>Kent, UK	

Abbreviations and glossary

AE	Adverse event
AR	Adverse reaction
CF	Consent form
CI	Chief Investigator
CRF	Case Report Form
ERC	Endpoint Review Committee
IB	Investigator’s Brochure
IDMC	Independent Data Monitoring Committee
PI	Principal Investigator
PIS	Patient information Sheet
QL	Quality of life
SAE	Serious adverse event
SOP	Standard operating procedures
SPC	Summary of product characteristics
SSA	Site specific assessment
SUSAR	Suspected unexpected serious adverse reaction
TMG	Trial Management Group
TSC	Trial Steering Committee
UAR	Unexpected adverse reaction

32.7 Figure 1: Diagram summarizing the HELIX trial

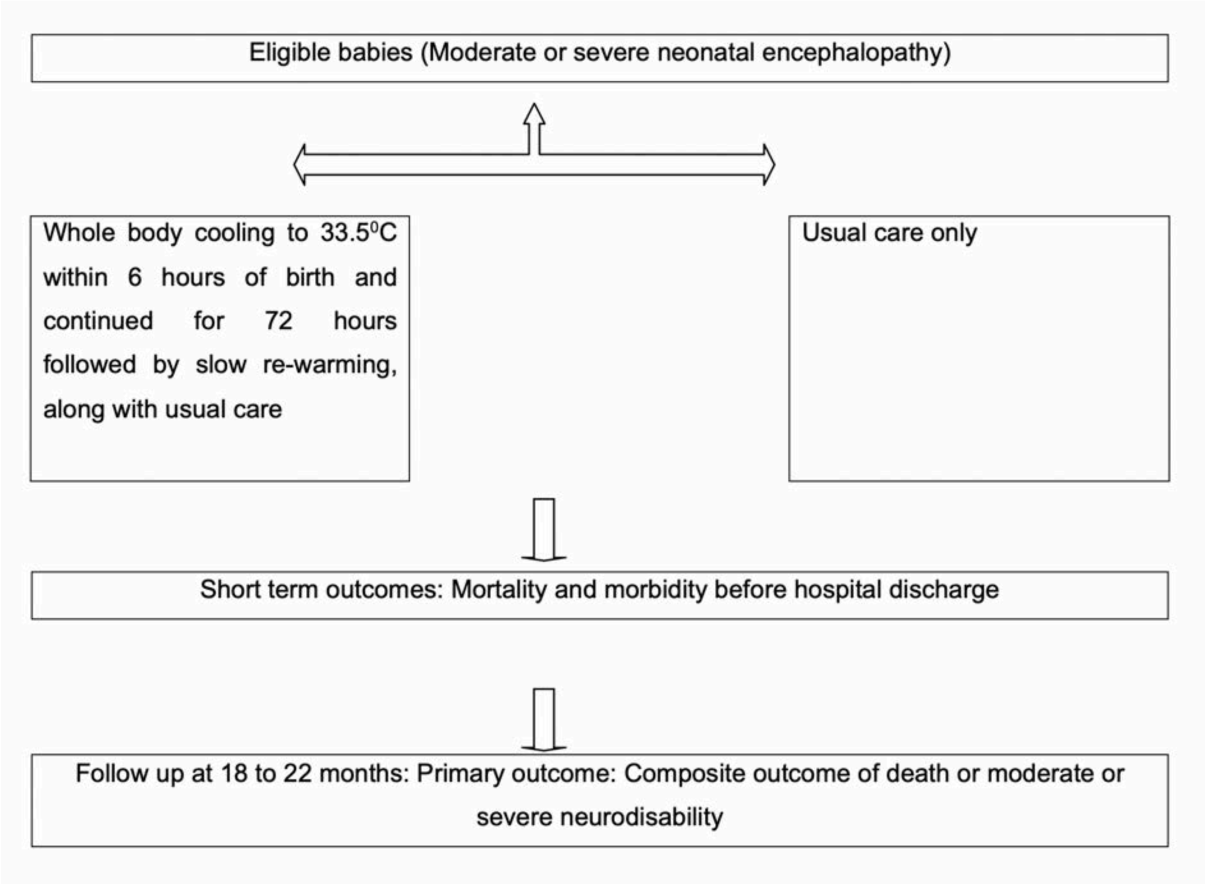

32.8 Annexe 1: Agreement and potential competing interests form

33 HELIX Trial: Agreement to join the Independent Data Monitoring Committee and disclosure of potential competing interests

Please complete the following document and return to the HELIX Trial Manager.

(please initial box to agree)

☐	I have read and understood the IDMC Charter version 0.1, dated 10/02/2015
☐	I agree to join the Independent Data Monitoring Committee for this trial
☐	I agree to treat all sensitive trial data and discussions confidentially

The avoidance of any perception that members of a IDMC may be biased in some fashion is important for the credibility of the decisions made by the IDMC and for the integrity of the trial.

Possible competing interest should be disclosed via the HELIX trial manager. In many cases simple disclosure up front should be sufficient. Otherwise, the (potential) IDMC member should remove the conflict or stop participating in the IDMC. **Table 1** lists potential competing interests.

☐ **No,** I have no competing interests to declare
☐ **Yes,** I have competing interests to declare (please detail below)

Please provide details of any competing interests:

____

____

____

Name: ___________________________

Signed: __________________________ Date: ______________

33.1

33.1.1.1.1 Table 1: Potential competing interests

- Stock ownership in any commercial companies involved
- Stock transaction in any commercial company involved (if previously holding stock)
- Consulting arrangements with the Sponsor (including CI for other Imperial College London trials)
- Frequent speaking engagements on behalf of the intervention
- Career tied up in a product or technique assessed by trial
- Hands-on participation in the trial
- Involvement in the running of the trial
- Emotional involvement in the trial
- Intellectual conflict e.g. strong prior belief in the trial's experimental arm
- Involvement in regulatory issues relevant to the trial procedures
- Investment (financial or intellectual) or career tied up in competing products
- Involvement in the publication in the form of authorship

33.2 Annexe 2: Suggested report from IDMC to TSC where no recommendations are being made

[Insert date]

To: Chair of Trial Steering Committee
Via: TSC Facilitator

Dear *[Chair of Trial Steering Committee]*

The Independent Data Monitoring Committee (IDMC) for the *[insert trial name]* trial met on *[meeting date]* to review its progress and interim accumulating data. *[List members] attended* the meeting and reviewed the report.

The IDMC should like to congratulate the investigators and trial team on the running of the trial and its recruitment, data quality and follow-up. The trial question remains important and, on the basis of the data reviewed at this stage, we recommend continuation of the trial according to the current version of the protocol *[specify protocol version number and date]* with no changes.

We shall next review the progress and data *[provide approximate timing]*

Yours sincerely,

[Name of meeting Chair]
34 Chair of Independent Data Monitoring Committee

On behalf of the IDMC (all members listed below)

IDMC members:
(1) [*Insert name and role*]
(2) [*Insert name and role*]

34.1.1.1.1 (3) [Insert name and role]

34.1.1.1.2

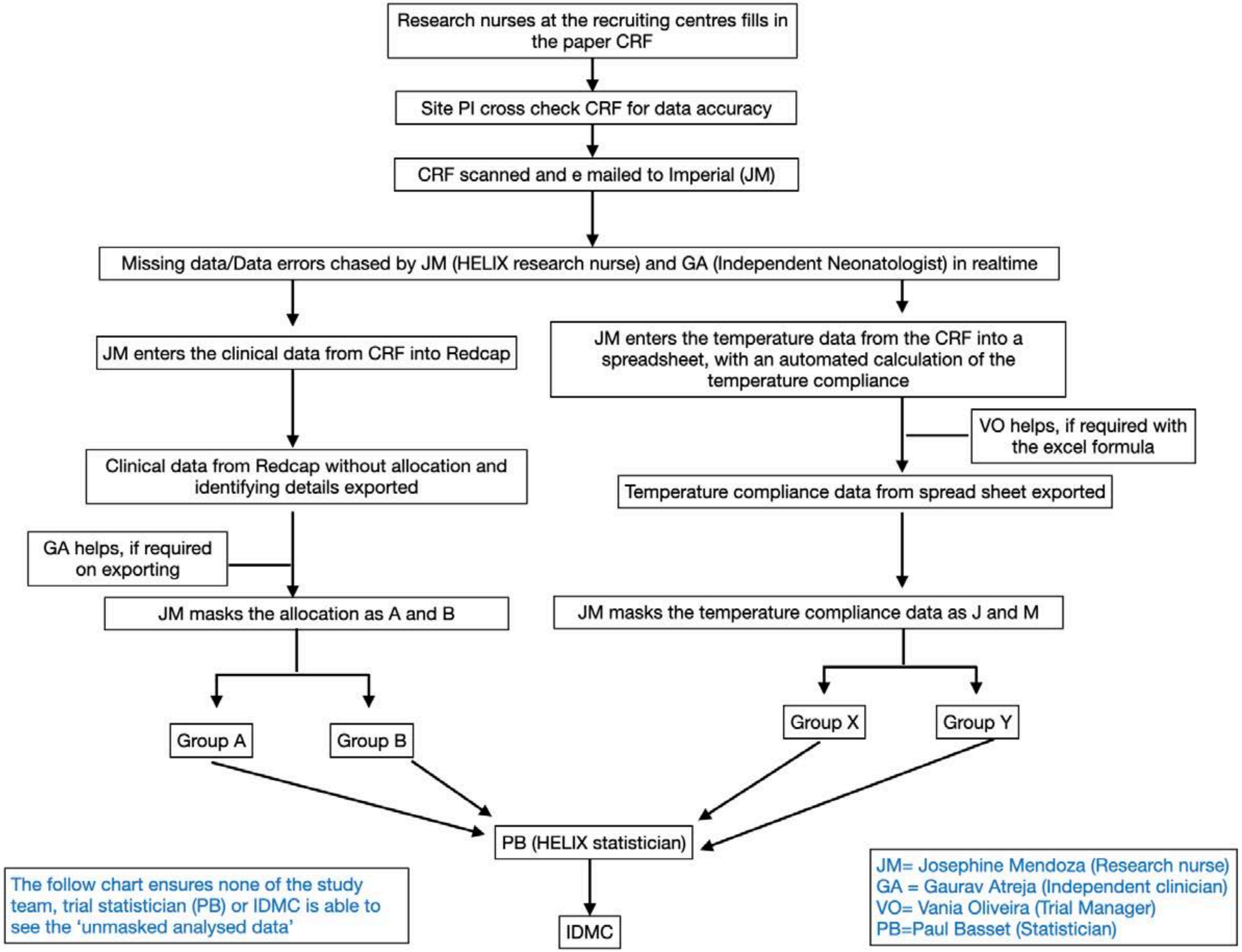
Research nurses at the recruiting centres fills in the paper CRF
Site PI cross check CRF for data accuracy
CRF scanned and e mailed to Imperial (JM)
Missing data/Data errors chased by JM (HELIX research nurse) and GA (Independent Neonatologist) in realtime
JM enters the clinical data from CRF into Redcap
JM enters the temperature data from the CRF into a spreadsheet, with an automated calculation of the temperature compliance
VO helps, if required with the excel formula
Clinical data from Redcap without allocation and identifying details exported
Temperature compliance data from spread sheet exported
GA helps, if required on exporting
JM masks the allocation as A and B
JM masks the temperature compliance data as J and M
Group A
Group B
Group X
Group Y
PB (HELIX statistician)
IDMC
The follow chart ensures none of the study team, trial statistician (PB) or IDMC is able to see the 'unmasked analysed data'
JM= Josephine Mendoza (Research nurse)
GA = Gaurav Atreja (Independent clinician)
VO= Vania Oliveira (Trial Manager)
PB=Paul Basset (Statistician)

Annexe 3: Summarise changes of IDMC from previous version

Version 1.0
This is version 1.0 of the IDMC charter for this trial. There are no changes to be reported.
