# Supplementary material for: Whole-Body Hypothermia, Cerebral Magnetic Resonance Biomarkers, and Outcomes in Neonates With Moderate or Severe Hypoxic-Ischemic Encephalopathy Born at Tertiary Care Centers vs Other Facilities: A Nested Study Within a Randomized Clinical Trial
Source: JAMA Netw Open. 2023 May 8;6(5):e2312152. doi: 10.1001/jamanetworkopen.2023.12152 (PMC10167567; doi:10.1001/jamanetworkopen.2023.12152)
Supplement: Supplement 2. — eTable 1. Baseline Characteristics of Inborn and Outborn Neonates eTable 2. Clinical Characteristics of Neonates With and Without MR Imaging (MRI) eFigure 1. Flowchart eFigure 2. Mean (SD) Rectal Temperature of Inborn (Top Panel) and Outborn (Bottom Panel) Neonates eFigure 3. Treatment Effects of Whole-Body Hypothermia on Whole-Brain White Matter Fractional Anisotropy Quantified by Tract-Based Spatial Statistics Among Inborn (A) and Outborn (B) Neonates [file jamanetwopen-e2312152-s002.pdf]

Supplementary Online Content

Thayyil S, Montaldo P, Krishnan V, et al. Whole-body hypothermia, cerebral magnetic resonance biomarkers, and outcomes in neonates with moderate or severe hypoxic-ischemic encephalopathy born at tertiary care centers vs other facilities: a nested study within a randomized clinical trial. *JAMA Netw Open*. 2023;6(5):e2312152. doi:10.1001/jamanetworkopen.2023.12152

eTable 1. Baseline Characteristics of Inborn and Outborn Neonates

eTable 2. Clinical Characteristics of Neonates With and Without MR Imaging (MRI)

eFigure 1. Flowchart

eFigure 2. Mean (SD) Rectal Temperature of Inborn (Top Panel) and Outborn (Bottom Panel) Neonates

eFigure 3. Treatment Effects of Whole-Body Hypothermia on Whole-Brain White Matter Fractional Anisotropy Quantified by Tract-Based Spatial Statistics Among Inborn (A) and Outborn (B) Neonates

This supplemental material has been provided by the authors to give readers additional information about their work.

© 2023 Thayyil S et al. *JAMA Network Open*.

eTable 1. Baseline Characteristics of Inborn and Outborn Neonates

	N	Inborn (n=123)	N	Outborn (n=285)	P-value
Maternal age, year	117	25.7 (5.0)	278	23.7 (4.2)	0.001
Booked pregnancies*	123	122 (99.2%)	278	264 (94.9%)	0.02
Primigravida	123	67 (54.5%)	284	168 (59.2%)	0.38
Diabetes	123	1 (0.8%)	285	1 (0.4%)	1.00
Hypertension	123	8 (6.5%)	285	5 (1.7%)	0.02
Thyroid disorders	123	0	285	1 (0.4%)	1.00
Complications of delivery±	122	43 (35.2%)	267	35 (13.1%)	0.01
Maternal pyrexia	121	5 (4.1%)	267	3 (1.1%)	0.02
Rupture of membranes >24h	121	0	262	4 (1.5%)	0.06
Meconium-stained amniotic fluid	123	47 (38.2%)	269	63 (23.4%)	0.0001
Reduced fetal movements	114	13 (11.4%)	221	11 (4.9%)	0.0001
Fetal heart rate decelerations	116	16 (13.8%)	208	7 (3.4%)	0.0001
Funisitis	121	21 (17.3%)	245	45 (18.4%)	0.01
**Perinatal sentinel events±**	123	20 (16.3%)	285	23 (8.1%)	0.02
Cord prolapse	123	4 (3.3%)	285	6 (2.1%)	0.50
Cord around neck	123	2 (1.6%)	285	6 (2.1%)	1.00
Prolonged second stage	123	7 (5.7%)	285	4 (1.4%)	0.05
Obstructed labor	123	2 (1.6%)	285	3 (1.1%)	0.64
Shoulder dystocia	123	0	285	1 (0.4%)	1.00
Antepartum hemorrhage	123	5* (4.0%)	285	3** (1.1%)	0.37
**Mode of delivery**					
Instrumental delivery	123	18 (14.6%)	283	22 (7.8%)	0.01
Pre-labor caesarean delivery	123	3 (2.4%)	283	0	
In-labor caesarean delivery	123	32 (26.0%)	283	48 (16.9%)	

© 2023 Thayyil S et al. *JAMA Network Open*.

Spontaneous vaginal delivery	123	70 (56.9%)	283	213 (75.3%)	
**Condition at birth**					
Cord blood pH at delivery	33	6.92 (0.18)	13	7.04 (0.30)	0.73
Apgar score at 5 min, median [interquartile range]	122	4 [3-5]	150	4 [2-5]	0.21
Apgar score at 10 min, median [interquartile range]	98	6 [5-7]	34	5 [4-7]	0.09
Endotracheal ventilation at birth	123	97 (78.9%)	278	81 (29.1%)	0.001
**Infant size and condition**					
Birth weight, grams	123	2815 (460)	285	2924 (448)	0.02
Birth weight < 2 standard deviations	123	28 (22.8%)	285	36 (12.6%)	0.13
Head circumference, cm	122	34.0 (1.7)	284	34.2 (1.3)	0.21
Head circumference < 2 SD from the mean	122	6 (4.9%)	284	8 (2.8%)	0.47
Age of neonate (Gestation, weeks)	123	38.5 (1.5)	284	38.7 (1.2)	0.18
Age at admission to the NICU at the cooling centre, minutes	123	30 [15-50]	285	180 [123-240]	0.001
Males	123	80 (65.0%)	285	187 (65.6%)	0.91
Moderate encephalopathy	123	94 (76.4%)	285	234 (82.1%)	0.22
Severe encephalopathy		29 (23.6%)		51 (17.9%)	
Rectal temperature at randomisation	120	35.9 (1.1)	269	36.0 (0.9)	0.36
Clinical seizures at admission	123	75 (61.0%)	285	224 (78.6%)	<0.001
MR imaging performed	123	82 (66.7%)	285	202 (70.9%)	0.41

Data are number of patients (%), mean (SD), or median [IQR]; *2 placenta previa, 3 abruptions; **3 abruption; ±Not mutually exclusive.

© 2023 Thayyil S et al. *JAMA Network Open*.

eTable 2. Clinical Characteristics of Neonates With and Without MR Imaging (MRI)

	**MRI performed (n=284)**	**MRI not performed (n=124)**	**P Value**
Birth weight, grams	2918 (428)	2832 (507)	0.08
Head circumference, cm	34.1 (1.5)	34.5 (1.5)	0.006
Head circumference < 2 SD	10 (3.5%)	4 (3.2%)	0.099
Age of neonate (Gestation, weeks)	38.7 (1.3)	38.6 (1.2)	0.46
Age at admission to the NICU, minutes, median [IQR]	139 [60-231]	140 [56-227]	0.98
Cord blood pH at delivery	6.9 (0.2)	6.8 (0.3)	0.06
Apgar score at 5 min, median [IQR]	5 [4-6]	4 [3-5]	0.004
Apgar score at 10 min, median [IQR]	6 [5-7]	5 [4-7]	0.04
Intubation at birth	106 (37.3%)	72 (58.1%)	<0.001
Antepartum hemorrhage	4 (1.4%)	4 (3.2%)	0.25
**Place of birth**			
Inborn babies	82 (28.9%)	41 (33.1%)	0.41
Outborn babies	202 (71.1%)	83 (66.9%)	
**Severity of encephalopathy at randomisation**			
Moderate encephalopathy	254 (89.4%)	74 (59.7%)	<0.001
Severe encephalopathy	30 (10.6%)	50 (40.3%)	
**Neonatal morbidity**			
Gastric bleeding	30 (10.6%)	66 (53.2%)	<0.001
Pulmonary hemorrhage	15 (5.3%)	55 (44.3%)	<0.001
Prolonged coagulation	56 (19.7%)	75 (60.5%)	<0.001

© 2023 Thayyil S et al. *JAMA Network Open*.

Culture proven early onset sepsis	17 (6.0%)	5 (4.0%)	0.24
Severe thrombocytopenia	22 (7.7%)	26 (21.0%)	<0.001
Persistent metabolic acidosis	9 (3.2%)	61 (49.2%)	<0.001
Persistent pulmonary hypertension	12 (4.2%)	28 (22.6%)	<0.001
**Outcomes at 18 to 22 months**			
Death up until 18 months	25 (8.8%)	122 (98.4%)	<0.001
Death, moderate or severe disability	70 (24.6%)	122 (98.4%)	<0.001

Data are number of patients (%), mean (SD), or median [IQR]

© 2023 Thayyil S et al. *JAMA Network Open*.

eFigure 1. Flowchart

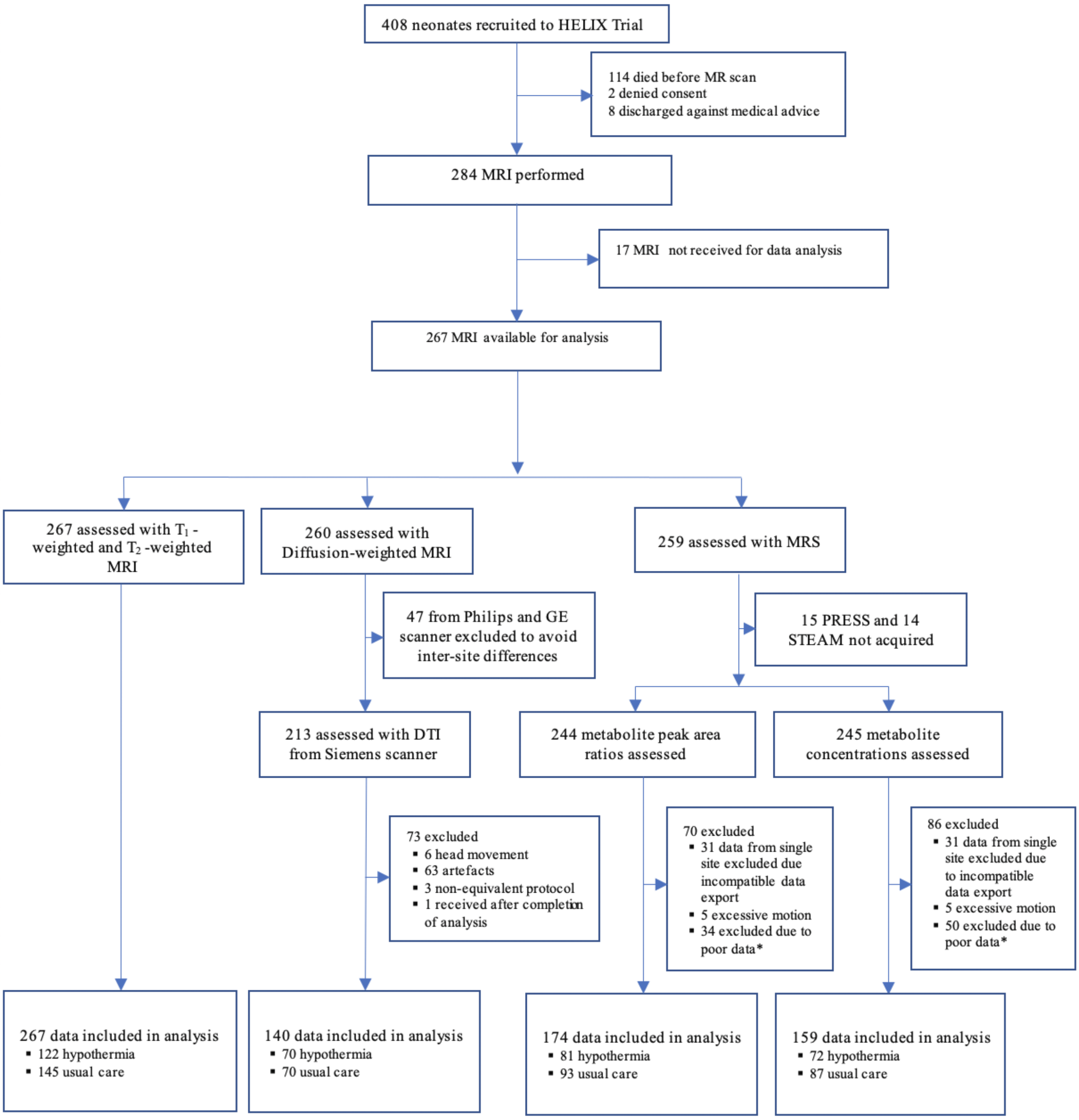

Thayyil et al. *Lancet Global Health*. 2021
DOI:https://doi.org/10.1016/S2214-109X(21)00264-3

© 2023 Thayyil S et al. *JAMA Network Open*.

eFigure 2. Mean (SD) Rectal Temperature of Inborn (Top Panel) and Outborn (Bottom Panel) Neonates

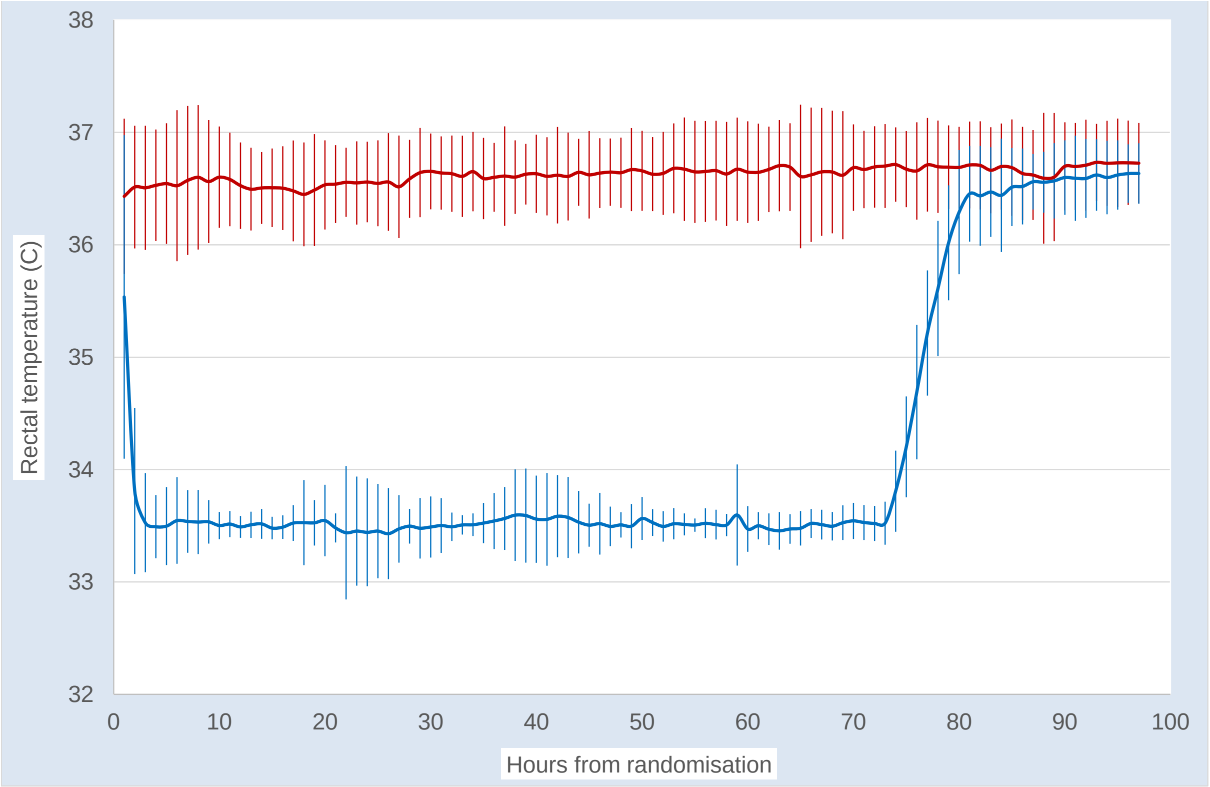

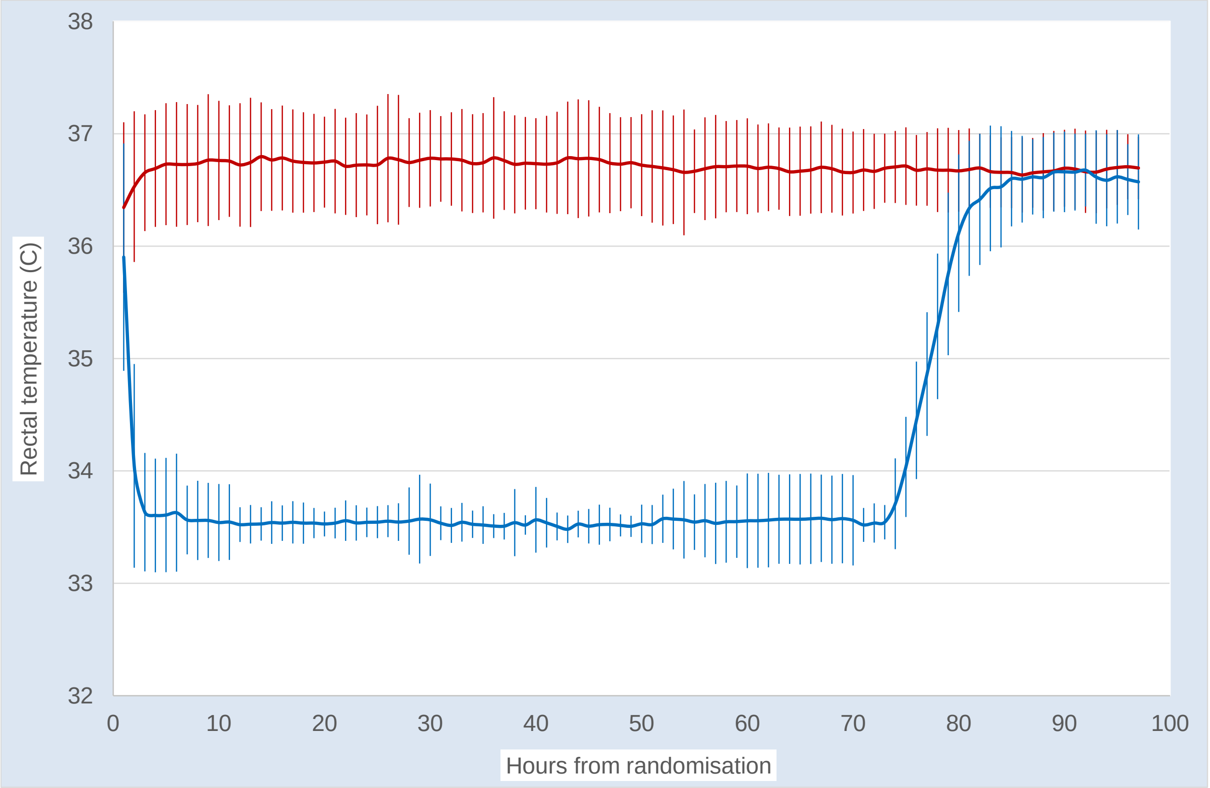

© 2023 Thayyil S et al. *JAMA Network Open*.

eFigure 3. Treatment Effects of Whole-Body Hypothermia on Whole-Brain White Matter Fractional Anisotropy Quantified by Tract-Based Spatial Statistics Among Inborn (A) and Outborn (B) Neonates

Mean fractional anisotropy tract skeleton with threshold range of 0.15 (lower) and 1.0 (upper) over regions showed no statistically significant groupwise differences in fractional anisotropy between control or hypothermia babies in either inborn (A) or outborn (B) groups. Statistically significant (p<0.05) regions would be colored in red and yellow.

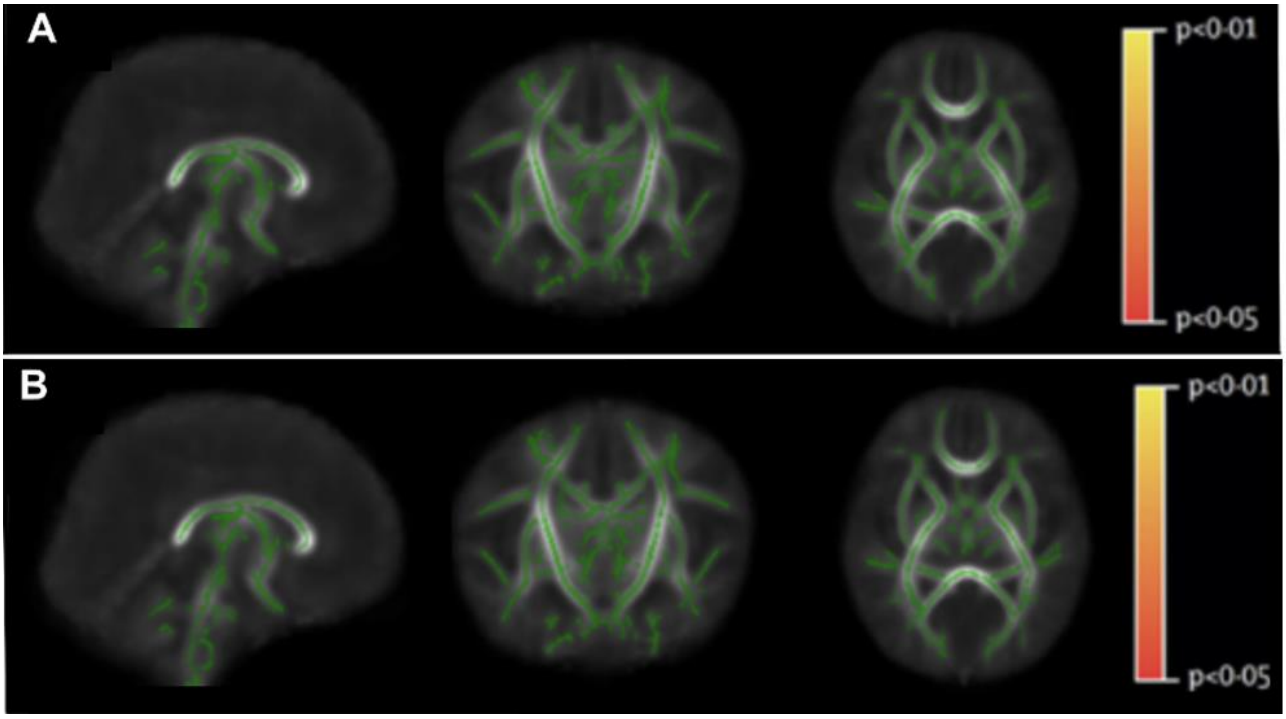

© 2023 Thayyil S et al. *JAMA Network Open*.
