# Supplementary material for: Whole-Body Hypothermia, Cerebral Magnetic Resonance Biomarkers, and Outcomes in Neonates With Moderate or Severe Hypoxic-Ischemic Encephalopathy Born at Tertiary Care Centers vs Other Facilities: A Nested Study Within a Randomized Clinical Trial
Source: JAMA Netw Open. 2023 May 8;6(5):e2312152. doi: 10.1001/jamanetworkopen.2023.12152 (PMC10167567; doi:10.1001/jamanetworkopen.2023.12152)
Supplement: Supplement 3. — Data Sharing Statement [file jamanetwopen-e2312152-s003.pdf]

Data Sharing Statement

Thayyil. Whole-Body Hypothermia, Cerebral Magnetic Resonance Biomarkers, and Outcomes in Neonates With Moderate or Severe Hypoxic-Ischemic Encephalopathy Born at Tertiary Care Centers vs Other Facilities. *JAMA Netw Open*. Published May 08, 2023. doi:10.1001/jamanetworkopen.2023.12152

Data
Data available: No

Additional Information
Explanation for why data not available: Additional secondary papers are currently being written up. Once these are reported the data will be made publicly available
